# Multi-resolution Correlative Ultrastructural and Chemical Analysis of Carious Enamel by Scanning Microscopy and Tomographic Imaging

**DOI:** 10.1021/acsami.3c08031

**Published:** 2023-07-31

**Authors:** Cyril Besnard, Ali Marie, Sisini Sasidharan, Petr Buček, Jessica M. Walker, Julia E. Parker, Matthew C. Spink, Robert A. Harper, Shashidhara Marathe, Kaz Wanelik, Thomas E.J. Moxham, Enrico Salvati, Konstantin Ignatyev, Michał M. Kłosowski, Richard M. Shelton, Gabriel Landini, Alexander M. Korsunsky

**Affiliations:** † MBLEM, Department of Engineering Science, University of Oxford, Parks Road, Oxford, Oxfordshire OX1 3PJ, U.K.; ‡ TESCAN-UK Ltd., Wellbrook Court, Girton, Cambridge CB3 0NA, U.K.; § Diamond Light Source Ltd., Didcot, Oxfordshire OX11 0DE, U.K.; ∥ School of Dentistry, University of Birmingham, 5 Mill Pool Way, Edgbaston, Birmingham, West Midlands B5 7EG, U.K.; ⊥ Research Complex at Harwell, Harwell Campus, Didcot OX11 0FA, U.K.

**Keywords:** human carious enamel, synchrotron spectroscopy, nano-X-ray fluorescence spectroscopy, nanodiffraction, ptychography, FIB-SEM, tomography

## Abstract

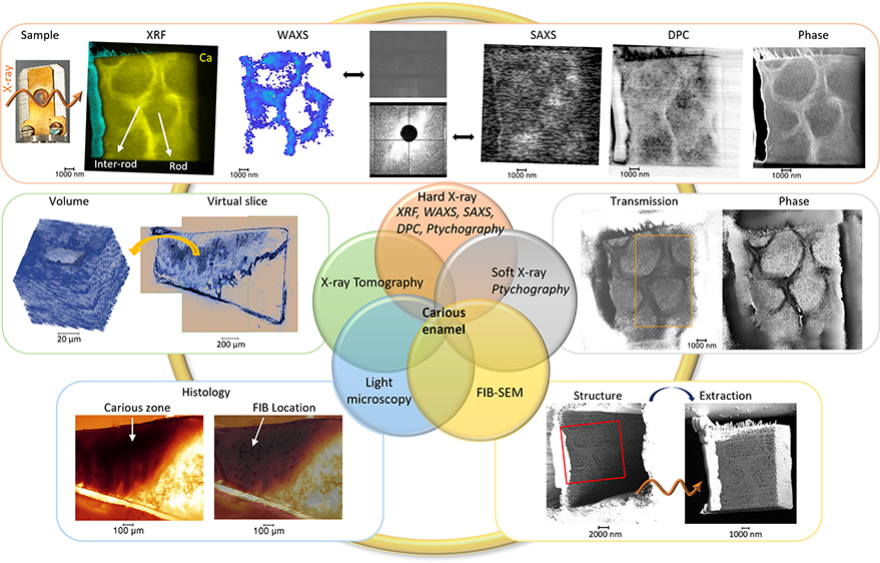

Caries, a major global disease associated with dental enamel demineralization, remains insufficiently understood to devise effective prevention or minimally invasive treatment. Understanding the ultrastructural changes in enamel is hampered by a lack of nanoscale characterization of the chemical spatial distributions within the dental tissue. This leads to the requirement to develop techniques based on various characterization methods. The purpose of the present study is to demonstrate the strength of analytic methods using a correlative technique on a single sample of human dental enamel as a specific case study to test the accuracy of techniques to compare regions in enamel. The science of the different techniques is integrated to genuinely study the enamel. The hierarchical structures within carious tissue were mapped using the combination of focused ion beam scanning electron microscopy with synchrotron X-ray tomography. The chemical changes were studied using scanning X-ray fluorescence (XRF) and X-ray wide-angle and small-angle scattering using a beam size below 80 nm for ångström and nanometer length scales. The analysis of XRF intensity gradients revealed subtle variations of Ca intensity in carious samples in comparison with those of normal mature enamel. In addition, the pathways for enamel rod demineralization were studied using X-ray ptychography. The results show the chemical and structural modification in carious enamel with differing locations. These results reinforce the need for multi-modal approaches to nanoscale analysis in complex hierarchically structured materials to interpret the changes of materials. The approach establishes a meticulous correlative characterization platform for the analysis of biomineralized tissues at the nanoscale, which adds confidence in the interpretation of the results and time-saving imaging techniques. The protocol demonstrated here using the dental tissue sample can be applied to other samples for statistical study and the investigation of nanoscale structural changes. The information gathered from the combination of methods could not be obtained with traditional individual techniques.

## Highlights

1. Ptychography using hard X-rays and soft X-rays provides complementary imaging of carious human dental enamel.
2. A hard X-ray nanoprobe (beam size down to ∼50 nm^2^) in the spectroscopic (XRF) and scattering (SAXS-WAXS) rastering mode allows for ultrastructural mapping of carious lesions in human dental enamel.
3. Differential phase contrast (DPC) imaging in combination with prior FIB-SEM and X-ray tomography reveals the details of HAp nanocrystal demineralization within enamel rods.

## Introduction

Human enamel is an acellular tissue consisting of a fascinating 3D hierarchical structure of hydroxyapatite (HAp) nanocrystals with different orientations organized into rods with the addition of inter-rod substance, sheath region, and proteins.^1−6^ This unique structure gives the enamel remarkable mechanical properties.^7−10^ However, its relatively low resistance to acidic dissolution leads to tissue loss such as caries. Caries, a major oral disease,^4,11^ progresses via the chemical action of the acids produced by acidogenic bacteria within a dental plaque biofilm,^12^ with alternating processes of demineralization and remineralization in the oral environment that differ from artificial lesions due to acid etching. This leads to demineralization with chemical modification, structural alteration, and the reduction in the mechanical stiffness and strength of enamel.^13−17^ To assess the sequence of changes in this linked chain, it is necessary to analyze the physicochemical enamel composition variations at the rod (micrometer) and crystallite (nanometer) scales.

The investigation of pathological tissues and artificial demineralization is crucial for understanding the disease process and developing new treatments such as remineralization procedures and artificial enamel as reported by Deyhle et al. and Zhao et al. studies.^17−19^ To develop materials for clinical applications the mimicking properties of natural enamel, it is necessary to characterize the structure and chemical composition of tissues in healthy and disease conditions down to the nanometer scale. At this scale, the complex organization of the crystallites determines the properties of enamel. The present study employs multi-modal correlative analysis of carious enamel via ptychographic and tomographic imaging and nanoscale chemical and crystallographic mapping. The different readouts obtained from the same sample lead to reach information in the characterization of the materials^4,17,20^ to capture various information at the micron level and below the sub-micron level.

Even though various analytical techniques have been used to study enamel carious tooth (both chemical and structural), nanoscale characterization of specific zones in the tooth (e.g., carious, non-carious, and surface zone, which have been reported^4^) has not been reported extensively on the same samples using thicker focused ion beam (FIB)-lamellae in comparison to transmission electron microscopy (TEM). Many of the recent nanoresolution studies were carried out for healthy enamel, artificial demineralization, and caries on thin samples.^21−23^ However, caries demineralization leads to complex and profound modifications that require deep analysis via the direct correlation between different relevant techniques (including tomography and electron microscopy) and covering large volume.^4^

Synchrotron X-ray facilities provide X-ray beams and techniques suitable for the nanoscale characterization of materials, benefiting from the advantages of access to soft and hard X-rays, tunable energy, fine-focusing capabilities, and measurement of different modes of interaction between beams and matter that give rise to a variety of individual and combined techniques for imaging, spectroscopy analysis, and scattering.

In most cases, the spatial resolution of X-ray studies of dental enamel reported previously was relatively coarse as compared with the typical enamel crystallite size (∼20–170 nm).^22,24^ With the development of nanoprobe techniques, at a synchrotron facility, the present authors deemed appropriate to apply these methods for the study of carious enamel (see Supporting Information (SI) Note 1 for nanoprobe analysis) as an extension of our recent work using nano X-ray fluorescence (XRF) spectroscopy, differential phase contrast (DPC) imaging, and ptychography.^16^

According to the literature reports, large volume X-ray tomographic imaging was carried out on various zones of carious and artificially demineralized enamel down to a voxel size of 325 nm,^4,14,25,26^ while dentine, another dental tissue was studied with voxel sizes down to 51 nm,^27^ including by ptychography-tomography with a reconstructed resolution of 158 nm.^28^ Recently, 2D ptychographic imaging of carious enamel has been reported covering only the carious region.^16^ Imaging studies have been combined with XRF, and even higher atomic resolution microscopy was carried out using atom probe tomography (APT).^23^ The crystallographic structure and texture variations have also been visualized by diffraction (X-ray diffraction (XRD) and wide-angle X-ray scattering (WAXS)) down to 500 and 250 nm beam size for enamel and 250 nm for cementum (tissue in the tooth), and the extraction of chemical information by combination with XRF has also been reported.^3,29−31^ Small-angle X-ray scattering (SAXS) of enamel using spot sizes on the sample down to 20 × 5 μm^2^ allowed obtaining reciprocal space information about the nanostructure in the carious region.^32^

The present study reports the nanostructural characterization of rods and inter-rods within lifted out FIB-lamellae from various locations of carious enamel. The organization of crystallites was visualized with ptychography, crystal lattice orientation was mapped,^33−35^ and calcium (Ca) distribution mapping based on Ca Kα fluorescence intensity from XRF was carried out. An important aspect of the study was the precise correlation (overlap) between images obtained by various techniques, demonstrating the capabilities of correlative nanoprobe characterization. Furthermore, enamel FIB-lamellae were prepared and imaged using FIB-scanning electron microscopy (SEM) and larger regions were imaged with synchrotron X-ray tomography. To gain complete information about the micro- and nanoscale structure of caries lesions, FIB-lamellae from different locations within lesions and of different thicknesses were screened (∼4.4 to 0.6 μm). This required strategies in the sample preparation, and there was also an importance to keep the sample from the analyses carried and avoid further sample modification during the preparation of the samples, e.g., slice and view using focused ion beam scanning electron microscopy (FIB-SEM)^4^ and staining.^10^ A full summary of the analysis is reported in the Graphical Abstract and Supporting Information (SI) including results (SI) and materials and methods (SI2).

## Results and Discussion

### Microscopy—Tomography—Locations for Nanocharacterization

**Figure 1:**
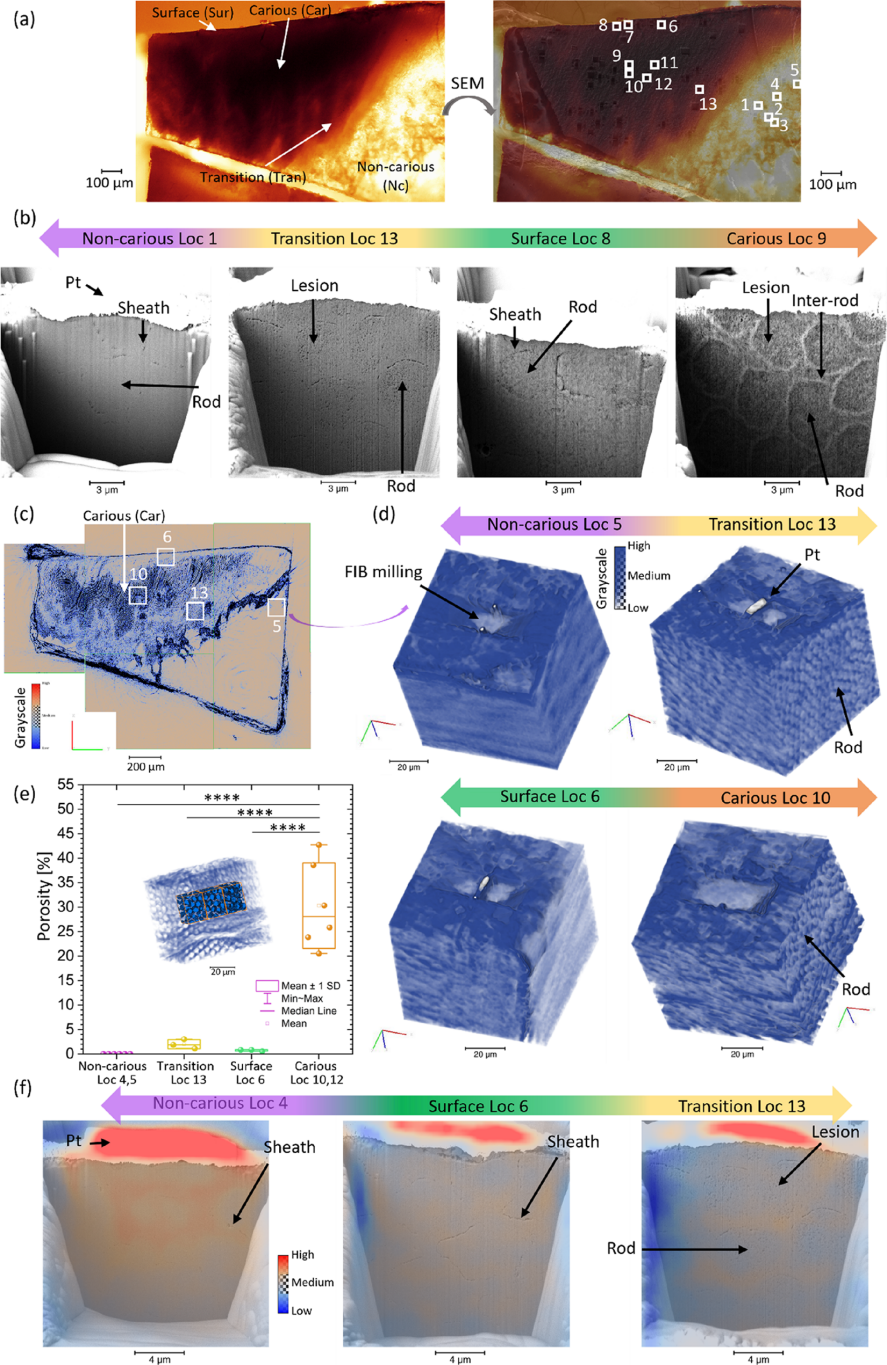
2D and 3D structural imaging analysis of several locations in carious enamel. (a) Light microscopy image of the carious sample and the superimposition of the light microscopy image with the SEM image and the highlight of the locations analyzed. (b) Backscattered electron imaging (BSi) of Loc 1, 13, 8, and 9 and (c) synchrotron tomography analysis of the region analyzed illustrated with a reconstructed virtual slice (extracted from the analysis of the synchrotron tomography data at the sample-distance, which was carried out in our previous study^4^) with an overall representation of the carious and non-carious enamel (data with a voxel size of 325 nm). (d) 3D representation of the synchrotron data (3D median filtered applied on the dataset) of Loc 5, 6, 10, and 13 with the visualization of rod shape in the carious region (dataset with a volume of 240 × 240 × 240 pixels). For Loc 13 and 6, the platinum (Pt) region can be seen, which is an artifact of FIB preparation (refer to the Methods Section); the locations are highlighted in (c). (e) Analysis of the porosity in the locations from various regions in the enamel, Loc 4, 5, 13, 6, 10, and 12 in the region of interest (three regions of 70 × 52 × 70 pixels from a large region of 240 × 240 × 240 pixels, voxel size of 0.325 μm) based on the methods previously described^4^ and 3D rendering of Loc 10 with the highlight of the regions of interest showing the segmented regions. A one-way ANOVA test with a post hoc Tukey’s test was carried out. **** represents *p* ≤ 0.0001. (f) Correlative imaging analysis of the virtual slice of the tomography (median filtered) and SEM image (BSi) of the cross sections of Loc 4, 6, and 13. More variations in the contrast of tomography were found in the carious region with the observation of the rods and lesion. For Loc 10, the correlative analysis was previously detailed in ref (4). Additional 3D rendering of Loc 4 and Loc 12 are detailed in SI-Figure S4. The location numbers marked in (a) were used in this paper. (modified with permission from ref (14). Copyright 2021 Elsevier Ltd.)

The FIB-lamellae from the carious enamel were selected to represent distinct locations of the carious process and the surface region as well as normal (pristine) enamel locations. The overview is presented in Figure 1a with the superposition of light and electron microscopy images and listed in SI2-Table S1. The differences in the structure (including the porosity more important in the carious region than in the non-carious region) in the subsurface regions at these locations were confirmed with FIB-SEM imaging after FIB milling of trenches using the methods previously described.^4^ This allowed for a judicious selection of FIB-lamellae for further characterization, Figure 1b and SI-Figures S1,2. In the carious region Loc 9, the structure of rods and inter-rods was clearly visible due to the effect of demineralization (Figure 1b). Porosity was found to be inhomogeneously distributed in the carious region. These SEM results were compared with the non-carious region Loc 1, showing significant differences in the structure with a lower porosity, and the sheath region, which has been previously described in enamel,^4^ could be distinguished. In the surface zone Loc 8, a less demineralized structure in comparison to the rest of the carious enamel was seen, in agreement with the previous description and quantification,^4^ as is evident from additional SEM images shown in SI-Figure S1. It was confirmed that both sides of the cross sections displayed these features, as can be seen from additional SEM images in SI-Figure S3a. Similar results were obtained for all locations. Bilateral observation (both sides) allowed a better understanding of the trajectory of the rods based on SEM imaging, as illustrated by the superimposition of the segmented region of the rod shape from the two sides (SI-Figure S3). In comparison with SEi, BSi showed an important bright region on the top of the cross section with a signal coming from Pt. The BSi could be used for contrast analysis with the higher atomic number appeared brighter^36,37^ and also had a reduction of charging in comparison to SEi.^38^

To obtain a better understanding of enamel internal structure over a larger size of volume (e.g., 815.425 × 815.425 × 685.425 μm^3^), volumetric images were acquired by absorption contrast synchrotron tomography at different sample-to-detector distances to produce a variation of contrast (see SI2-Figure S3). In the tomography data, the enamel structure was more prominent in the carious region in comparison to the non-carious and surface zones (more details in ref (4)), Figure 1c,d. Significant differences in the porosity analysis were found between the carious region and the other regions with higher porosity observed in the carious region (Figure 1e). Tomographic reconstructions were correlated with the SEM images of the cross sections, Figure 1c–e, providing microscale details of the rods and inter-rods regions. The visualization of these regions was carried out without the need for FIB milling in comparison to the FIB-lamellae for nanoprobes, and it also revealed the internal details of thick FIB-lamellae with a voxel size of 325 nm. For different locations (carious, non-carious, and surface region) within the sample, further elemental and crystallographic characterization was performed using XRF and diffraction scattering analysis (WAXS and/or SAXS), and the real space structure was imaged with ptychography and differential phase contrast (DPC) imaging on FIB-lamellae. We were able to correlate data from scattering and fluorescence with nanoresolution down to ∼50 nm.

### Chemical Analysis—X-ray Fluorescence (XRF) Spectroscopy

To characterize the chemistry at different parts of the enamel (carious, surface enamel, transition, and non-carious, Figure 1) and across different locations in the FIB-lamella (rods, inter-rods, and sheath, regions highlighted in Figure 1b), elemental mapping was carried out to determine the distribution of the elements and variations occurring in these regions. A clear localization and visualization of the FIB-lamellae prior to fine mapping were done based on the analysis of the elements calcium (Ca), platinum (Pt) (remaining on the FIB-lamella from the FIB process), and copper (Cu) (from the grid) (SI-Figure S5). Smaller regions of interest were then investigated with a beam size of ∼150 nm^2^ and 50 nm^2^ to cover various regions of the enamel (unfocus and focus respectively).

**Figure 2:**
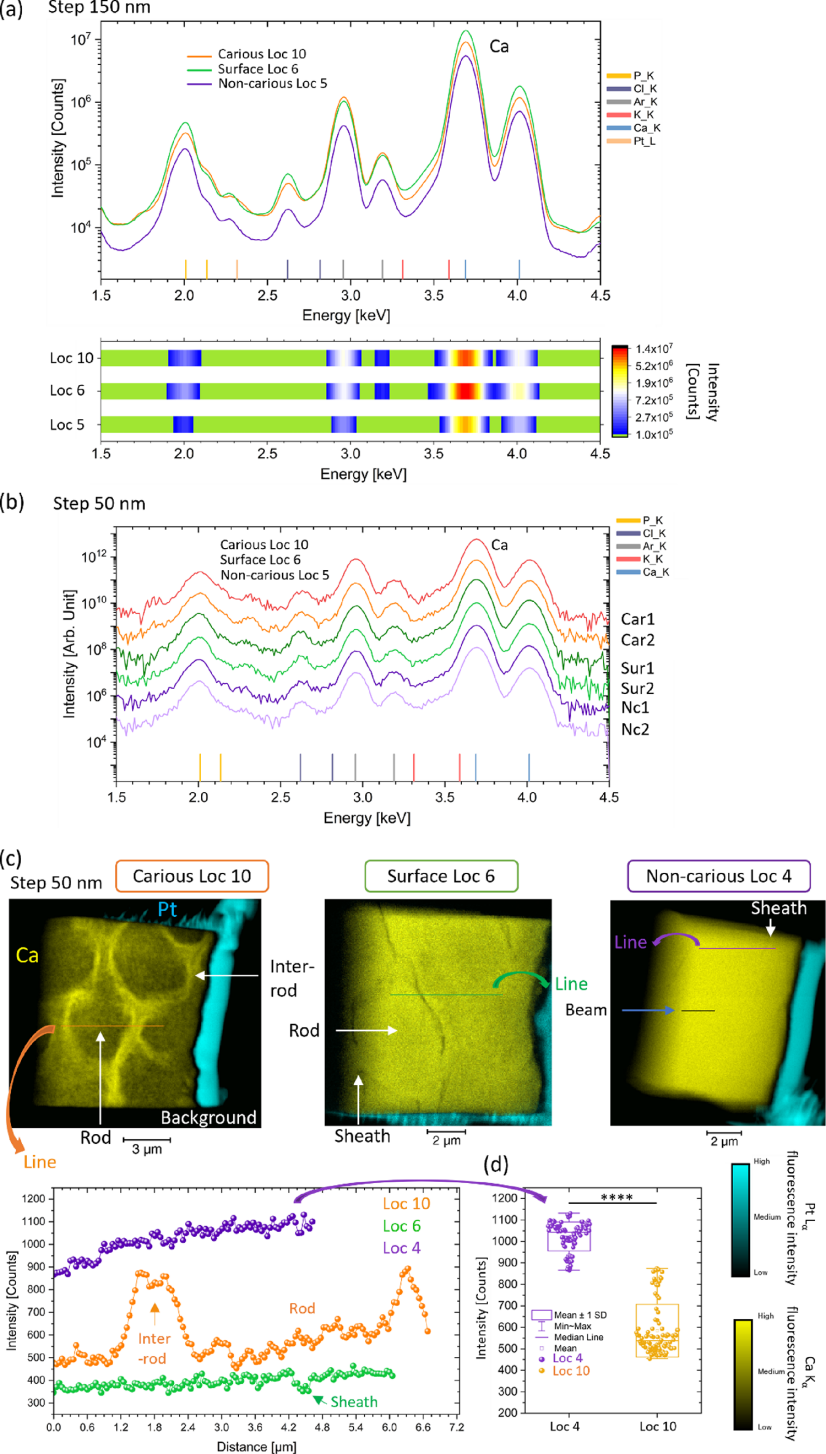
XRF analysis with the spectrum and map from enamel in various locations of the sample. (a) Spectrum of the sum of the intensity of the fluorescence signal of all the pixels in the region of interest of Loc 10, Loc 6, and Loc 5 using 18 keV X-rays (see SI-Figure S6 for the locations of these regions of interest on the FIB-lamellae and additional details for the step of 150 nm in SI-Figure S7), a step of 150 nm (unfocus). (b) Localized XRF analysis with the plot of the spectra from a pixel acquired in rods and inter-rods in Loc 10, in rods and around sheath in Loc 6, and in rods and inter-rods in Loc 5 (for the non-carious enamel lamella, the determination of the rods and inter-rods was based on the SEM images and DPC image), see SI-Figure S8 for the pixel location (“Car_1” referred to as one pixel for the carious region, “Sur” for surface, and “Nc” for non-carious), spectra acquired at 12 keV, and map with a step of 50 nm (DPC mapping also acquired). (c) XRF map of Loc 10, 6, and 4, which directly highlighted the differences in Ca Kα fluorescence intensity (in yellow) in the carious region with the two other regions, surface zone and non-carious enamel, Pt Lα fluorescence intensity is also shown (in cyan), which delimited the FIB-lamella, data acquired at 12 keV with a beam size ∼50 nm^2^. A plot of the profile of lines extracted from the three locations, line profile analysis with a step of 50 nm (focus). In the non-carious location, a line on the sample can be seen and was related to the beam. The different intensity scales in the plots are explained from different beam sizes used, regions acquired, beam energies, and acquisition times: 5 s for (a, b) and 0.015 s for (c). (d) Analysis of the Ca Kα fluorescence intensity of Loc 4 and 10 from the line profile in (c), from a distance of 4.56 μm (beam size of 55 × 45 nm^2^, exposure of 0.015 ms, and 12 keV). A two-sample *t*-test was carried out. **** represents *p* ≤ 0.0001.

In the non-carious region, Loc 5, XRF detected the main elements of enamel, e.g., Ca, as expected for the composition of enamel (HAp being Ca_10_(PO_4_)_6_(OH)_2_^39^), plus additional elements including argon (Ar) with a signal coming from the air (Figure 2a). On the XRF map of Ca (integrated Ca Kα fluorescence signal), a variation in the intensity of the signal was found at Loc 4 (SI-Figure S9) (line scan extracted from the map), but the difference in intensity was not significant enough to be able to determine the high-resolution details of the enamel structure as seen previously on the SEM (i.e., rod and inter-rod). This was also confirmed at Loc 1, 2, and 5 (SI-Figures S6, S7, and S9 with a summary of the XRF maps acquired at a step of 150 nm, unfocus). XRF mapping analysis closer to the crystallite dimension was carried out with a beam size ∼50 nm^2^. The spectrum analysis in the region of Loc 5 showed the main elements of enamel, and no significant differences in the peaks were found (Figure 2b). Map analyses were carried out for Loc 1, 2, 3, 4, and 5, and a similar observation to the previous resolution was found, seen with a lack of clarity in the identification of the enamel structure. However, the sheath could be noticed in a few regions (Figure 1c, SI-Figures S8 and S10).

The surface region with Loc 6 revealed similar features to the non-carious regions, with the main elements present and with similar spectra from rods and inter-rod regions (Figure 2a,b). From the XRF map, the sheath showed less intensity in Ca, which corresponds to features visible in the SEM image. However, except in the sheath region, no important variation in Ca fluorescence intensity was found in rods and inter-rods (SI-Figures S6 and S8 for the 150 nm step size and SI-Figures S7 and S9 at a step size of 50 nm).

In the carious region (Loc 10), the spectrum of the sum of intensity showed Ca, P, and additional elements as seen for the non-carious region, Pb (Figure 2a,b) (additional details in SI-Figures S6, S7,and S9 for 150 nm and SI-Figures S8 and S11 for the 50 nm step size). The adopted step size of 50 nm during focus analysis enabled localized spectral analysis to be carried out. XRF spectra were plotted along a line going through inter-rod boundaries in Loc 10 (SI-Figure S11). Variation of Ca Kα signal intensity was found with clear differences in intensity going from rod to inter-rod and a decrease of intensity between two boundary regions (covering a few pixels, see the map in SI-Figure S11), but no significant differences in the elements present were found. The localized analysis combined with the XRF map analysis of Loc 10 (in contrast to the non-carious region) revealed a clear variation in the Ca Kα intensity in different regions (Figure 2c,d) (SI-Figures S6, S7,and S9 for the 150 nm step size). Clusters of high intensity of Ca were found in the boundary of the inter-rod region in comparison to the rods (Loc 10, SI-Figure S6). These features were in line with the structure observed in the previously acquired SEM images^4^ (SI-Figures S2 and S12), where the brighter regions in SEM indicated the higher intensity of Ca. Significant differences in Ca intensity were found between the carious and non-carious (Figure 1d) with lower intensity in the carious region suggested from the demineralization. To confirm the XRF findings, other FIB-lamellae of carious enamel (Loc 9, 11, and 12) were probed with XRF and similar results were found (SI-Figure S9). This suggested that at least in part of the demineralized structure, there is anisotropy in the release of elements from the crystalline HAp structure during the dissolution of the sample. This could be also due to reprecipitation (considering carious has demineralization and remineralization processes), and therefore, more analysis will be required to elucidate this. With the line scan analysis, the rod and inter-rod regions were visualized in terms of their dimensions (SI-Figure S9b).

Although the XRF Ca distribution in the carious enamel is different from the non-carious enamel, some variations within the lesion were also seen. For example, for Loc 13, located in the transition region, the variation of Ca Kα intensity was less significant than the carious region and was suggested to originate from a decrease in demineralized structures (SI-Figure S9). Furthermore, owing to the fine step used when in focus (50 nm), local variations in the Ca Kα intensity were visualized in maps of rods and inter-rods, summarized in Figure 2c and SI-Figures S8 and S11 for Loc 10, and other locations in SI-Figure S10, using a lateral and vertical step size of 50 nm.

The importance of the localization of the region analyzed in the enamel was reinforced with the study of the surface zone, Loc 6, where less variation in Ca Kα intensity was found in comparison to the carious lesion (Figure 1c and SI-Figure S10 with a 50 nm step). The surface region contained a region with low Ca Kα intensity, which was assigned to the sheath (Figure 2c). In comparison to the non-carious region, the sheath was more apparent in the surface region and there was more variation of intensity, which could be due to partial demineralization, similar to what was detected as porosities in SEM images (Figure 1b and SI-Figure S1). These results were also seen in Loc 7 and 8 in the surface region.

These results reinforced the results of the SEM and tomography dataset, demonstrating a structure that was less demineralized in the non-carious and surface regions than the carious region. To the best of the authors’ knowledge, this was the highest XRF resolution analysis of several regions in enamel. It extended our previous work and a previous study on another dental tissue cementum analyzed with a beam diameter of 250 nm (both diffraction mapping and XRF).^30^ However, a higher resolution has been reported in other materials, such as bone with a beam size of 32.3 × 30.5 nm^2^.^40^ To go down to few nm, scanning transmission electron microscopy with electron-dispersive spectroscopy or electron energy loss spectroscopy can be used but would require thin FIB-lamella^4^ and thus less material analyzed than the data shown described here.

On the other hand, low-resolution analyses are useful for the characterization of larger scale features as well as for covering large tissue regions. In a preliminary analysis, XRF and X-ray absorption near edge structure (XANES) at the Ca K-edge based on the dichroism of the enamel were carried out with a resolution of 2 μm, which led to the characterization of the Hunter–Schreger bands, which are wavy structures observed in the enamel (SI-Figure S13).^21,41^ The acquired signals in these bands were different than the surrounding regions with variations in intensity and highlighted that a lower resolution, 2 μm, in this study was also needed for a larger view of the sample, also reported on normal enamel.^42^

### X-ray Scattering—Diffraction

**Figure 3:**
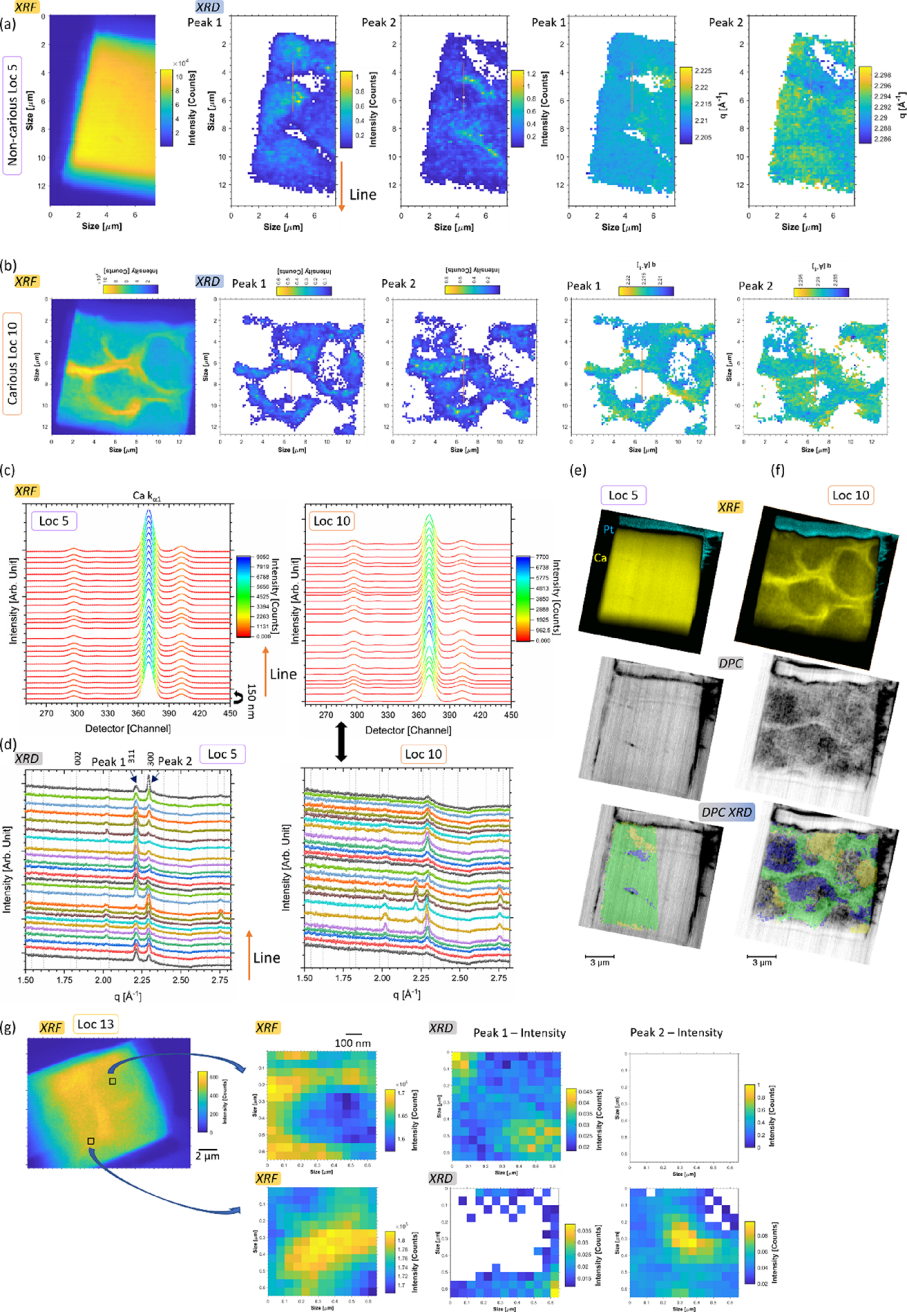
XRF/WAXS and correlative analysis of Loc 5 and Loc 10. (a) Map of the intensity of the Ca Kα fluorescence peak of Loc 5 in the non-carious region with the WAXS analysis and corresponding map of intensity and *q* range for the (311) and (300) peaks (peaks 1 and 2, respectively), step size of 150 nm (unfocus), 5 s exposure, and 18 keV energy. (b) Similar to (a) but the figure shows the analysis of Loc 10 in the carious region. In both locations, the analyses were modal XRF and WAXS 1D plots with line profiles passing through rod and inter-rod plotted in (c) for Loc 5 and (d) for Loc 10 (in the XRF plot, one channel is 10 eV). (e, f) Correlative analysis of the spectroscopy, structure analysis with DPC (beam size ∼50 nm^2^, more details in the section of DPC), and the map from the WAXS analysis of the intensity of peaks 1 and 2. Superimposition of the map of the intensity of the peaks 1 and 2 with the DPC image and the combination image of the WAXS analysis (in yellow only peak 1, blue peak 2, and green both peaks) superimposed on the DPC image (the details of the analysis are summarized in SI-Figure S14). This was carried out for (e) Loc 5 and (f) Loc 10. (g) Analysis of Loc 13: XRF map of Ca Kα fluorescence intensity (step of 50 nm and exposure of 0.015 ms) and analysis of regions of interest with the map of XRF Ca Kα intensity, WAXS with peaks 1 and 2 intensity, done for regions in rods and near inter-rods, 5 s exposure, a step of 50 nm, and 18 keV energy. For the diffraction analysis in (a, b), a beam size of 40 × 57 nm^2^ in focus prior to moving the stage by 75 μm for the analyses, and in (g), a beam size of 71 × 62 nm^2^.

To probe changes in the crystal structure, wide-angle X-ray scattering (WAXS) was performed. On the diffraction map (Figure 3a) of Loc 5 (non-carious), which was acquired simultaneously with the XRF map using a beam size of approximately of 150 nm^2^, clear modifications in the X-ray diffraction (XRD) patterns (after integration of the WAXS pattern) were found, as revealed in a line profile of the mapped data from rods and inter-rods (Figure 3a,c,d), which showed the difference in HAp structure from the intensity of the diffraction peaks and the variation of texture at a resolution of 150 nm. Clusters of regions of low scattering intensity were found on the sample, revealing a modification of the enamel structure, which was not detectable in the chemical XRF analysis. The diffraction patterns and the analysis of the two peaks (311) and (300) of HAp^43^ (referred to as peak 1 and peak 2 in this manuscript) revealed variations in the intensity of the peaks at different locations (additional details of the location of the peaks in SI-Figure S14). Overall, the (002) peak showed limited intensity in the patterns of the performed analysis, and this could be due either to a specific demineralization and/or to the orientation of the FIB-lamellae (from the SEM images when the rods were visualized, orientation was more with the X-ray beam along the rods) as well as damages from the FIB milling. Further studies will be required to explore the changes that FIB milling could make to the structure of the enamel, both structural and chemical, and this is specific to FIB milling and was delineated within this study.

Similarly, XRD and XRF mapping was carried out on another non-carious region, Loc 4, with also a variation of peak intensity and the visualization of texture on the sample in various locations (SI-Figure S15).

In carious enamel, the diffraction patterns revealed a less intense signal, as visualized by the colormap in Figure 3b. The detected signal came mainly from the inter-rod and less demineralized regions in the enamel as well as some regions in the rods. The shape of the rods could be visualized from the diffraction data and the changes in intensity from the analysis of the two peaks (Figure 3b,d). The peak (311) was found more often and more intense in the inter-rod region, and the signal was also found in the non-carious region (Figure 3a). In the carious sample, low intensity for the peak (311) was observed in several locations in comparison to normal enamel, which suggests that the crystal plane (311) was affected by the demineralization. Low-resolution maps were acquired at other locations: the surface region and the transition zone. The results were correlated to the DPC imaging by the superimposition of the different imaging modes, which revealed clear correlations (at the rod and inter-rod level) between the variation of peak intensities in the diffraction data and XRF analysis (Figure 3e,f and SI-Figures S14–S17). Analysis was performed using a ∼50 nm^2^ beam size in the regions of interest of Loc 4, 6, and 13 (non-carious, surface, and carious zone). This targeted the rods and inter-rods in each location (for the non-carious region, the SEM image was used as a reference for the features), and variations in the intensity of the diffraction peaks were detected. Figure 3e,f summarizes the results of the WAXS analysis with the correlation of fluorescence and WAXS maps for Loc 13. Local variation in the structure was visualized down to ∼50 nm^2^ beam size with lower intensity of peak 2 in rods than inter-rods, and local variation in the map was also found, seen with the cluster on this map (Figure 3g and SI-Figure S17). It was also found that in the diffraction patterns, organization of the crystal lattice changed as a function of location and there was a clear decrease in the intensity of the (300) peak in the inter-rod material. Based on the XRF and the chemical analysis of the sample, a direct correlation with the phase was possible. In the carious sample, peaks were less intense than those in the non-carious regions; this could be related to the amorphization of the sample caused by the demineralization, as previously reported^44^ and will require additional studies to confirm.

**Figure 4:**
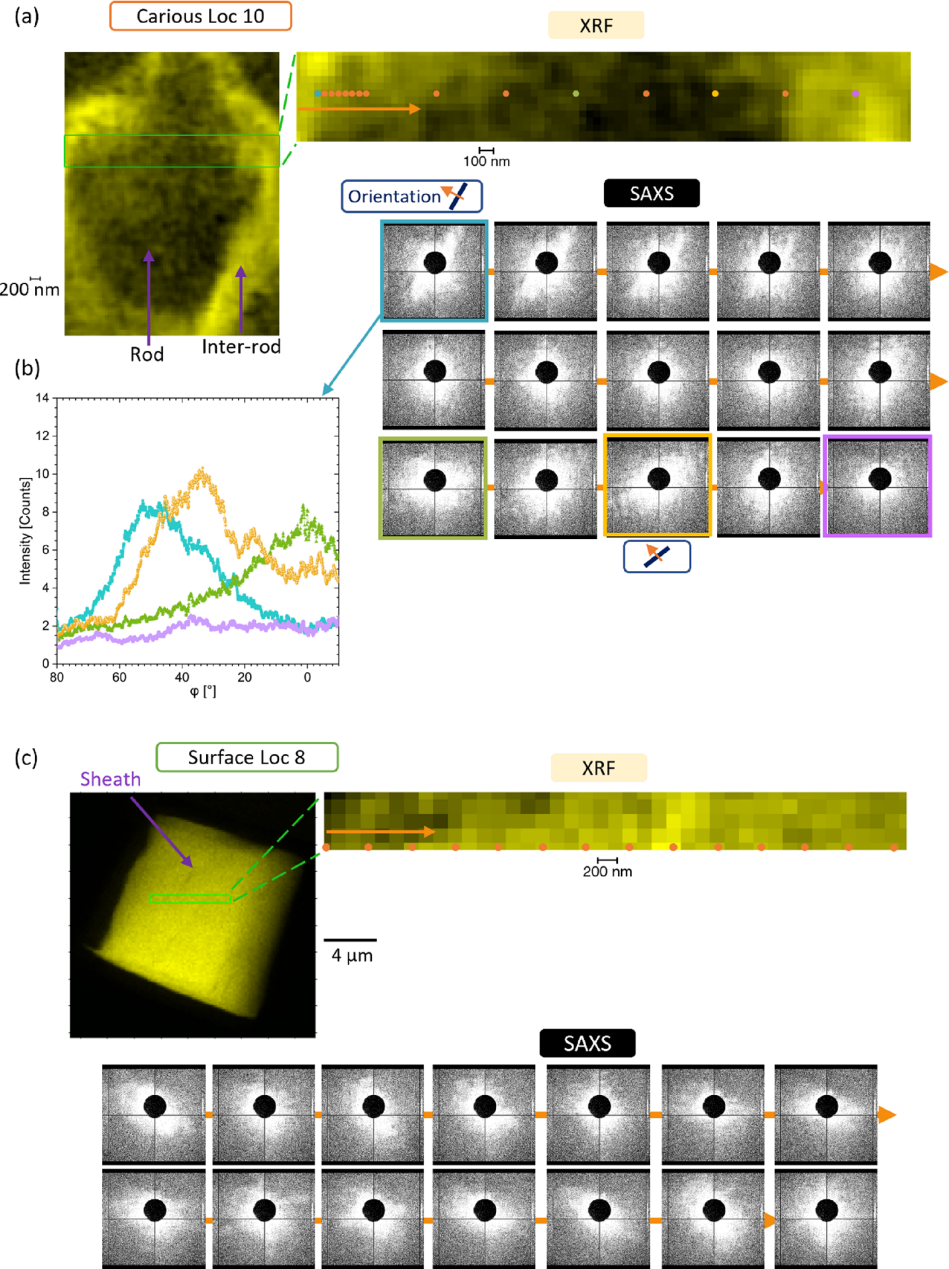
XRF/SAXS analysis of carious and surface regions. (a) XRF and SAXS analysis of Loc 10 with a step of 50 nm. XRF map of Ca with a step of 50 nm and exposure of 5 s and a region of interest (highlighted) extracted with the Ca intensity map plotted and extracted SAXS pattern from various pixels (color dots), going through a rod and inter-rod. Orientation of the scattering signal is highlighted in a few patterns (confirmed in another region, SI-Figure S18). The evolution of the SAXS patterns with evidence of coherent scattering is illustrated in Movie S1. Beam size of 55 × 45 nm^2^ for the analysis. (b) Plot of the SAXS intensity as a function of azimuthal angle φ from the four locations described in (a). (c) XRF and SAXS analysis of Loc 8 in the surface region. Ca XRF map acquired with a step of 150 nm, exposure of 0.015 ms, region of interest highlighted. The region of interest with the acquisition of XRF and SAXS, map of Ca acquired with a step of 150 nm, exposure of 5 s, simultaneously with SAXS (beam size of 55 × 45 nm^2^ in focus before moving the stage by 75 μm for the analysis). Extraction of SAXS patterns from various pixels showing less scattering than the carious sample. All of these measurements were done without a flight tube at 12 keV.

In addition, SAXS analysis was carried out to analyze the orientation effect and the local variation of scattering in rods and inter-rods. Some variations in the SAXS patterns were observed in the enamel structure, particularly in the carious regions, and these were correlated to the location on the FIB-lamella based on the XRF analysis. Figure 4 shows the extracted patterns from a region of interest in Loc 10 (carious region) acquired with a 50 nm step size. Variation in the orientation of the scattering pattern was seen going from one border of the rods to another as well as the details of the variation of alignment in the locations (Figure 4a);^45^ additional details for this location are shown in SI-Figure S18 and Movie 1. The SAXS scattering pattern was found to be different in terms of intensity in neighboring pixels, confirming the importance of this resolution to detect localized variations in contrast to previous SAXS analyses on carious samples,^17,46−48^ although the lower resolution can provide details of region as a first screening of a sample, which then needs a higher resolution for further characterization. The sum of intensities of the SAXS pattern from the overall pattern also highlighted variations in the enamel in the carious region (SI-Figure S19). For the non-carious region Loc 4, the scattering was less wide in the locations analyzed using a step of 50 nm (SI-Figure S20). Similar observations were made with a step of 150 nm in Loc 5 (non-carious, SI-Figure S20). The presence of wide scattering in the carious region was also detected in Loc 9 and 12 and in a thick sample Loc 11 (SI-Figures S21 and 22) as well as in the transition location Loc 13 (SI-Figure S22) and in the surface region (Figure 4b and SI-Figures S23 and S24). The scattering in these regions was suggested to arise from the partial demineralization of the structure visualized with the SEM in comparison to non-carious regions. The analysis showed that the unfocused and focused datasets with steps of 150 and 50 nm, respectively, were necessary to characterize enamel because of its multiscale structure and the complex organization down to the crystallite scale. The dimensions analyzed with SAXS in the enamel were covering the structure from a length scale of ∼15 to 60 nm in real space (equivalent to a scattering vector *q* of 0.04 to 0.01 Å^–1^, SI2-Figure S5), which brought another level of scale in comparison to WAXS. This complements the details visualized with SEM from topography and morphology and in addition led to details from the full thickness of the sample.^49^ One of the developments in the SAXS analysis is to carry out SAXS 3D reciprocal-space, tensor tomography to extract statistical information from SAXS patterns, e.g., main orientation.^50,51^ Although the methods have been used for various materials, there is limited information on enamel.^52−54^ Prior to the 3D analysis that requires long duration of analysis, it was required to understand what was occurring in 2D. Previous works on 3D SAXS were carried out at micron resolution; here, it is shown that SAXS was possible to use with a nanobeam size for 2D mapping and it could be extended to 3D analysis with dedicated equipment. This was a step further to previous SAXS analyses on human teeth covering larger regions but without nanobeam size and allowed not only a comparison from large carious and non-carious regions but from rods and inter-rod regions.

The previously analyzed samples on the Cu grid were in a dry condition. Based on the demonstration of the feasibility of the analysis at the nanoscale of enamel and differences of structure and chemistry between non-carious to carious regions, there was a motivation to analyze these changes *in situ*. Thus, a preliminary *in situ* experiment was carried out in a flow cell in I14 (DLS) (SI-Figure S25a); this presented preliminary results highlighting the additional exploration of the sample environment and beam effects, required to implement this technique more thoroughly. After an initial scanning of the sample in the cell, the overall condition of the sample did not significantly change according to the analysis of the Ca intensity. However, after multiple scans in water, the X-ray beam led to considerable degradation of the sample, as seen in SI-Figures S25 and S26 with the formation of a “trench” where the region of interest was scanned (important decrease in intensity, dark region). The damage was clearly identifiable with a localized decrease in intensity in the region scans, which created unwanted additional contributions of the alteration of the structure prior to acid contribution. During the circulation of acidic solution in the flow cell to demineralize the enamel, the same significant damage upon long exposure to the beam was observed. However, in comparison to the map from the sample circulated with just water (control), a decreased change in Ca intensity on the sample was found, suggesting that the degradation observed was contributed by the demineralization of the enamel due to the acid (SI-Figures S25b and S26). The decrease in Ca intensity with time during acid exposure was found non-linear from the regions analyzed and also location-dependent, highlighting the importance of local analysis (SI-Figure S26). A significant decrease in the Ca intensity was noticed with time (SI-Figure S26d). It is expected that radiolysis by the interaction of the liquid with the beam and thus with the sample played a role,^55^ observed using X-ray on the corrosion in metal and also reported in electron microscopy.^56,57^ More analyses will be required to study this phenomenon and avoid damages that are not related to the solution–sample interaction. DPC analysis of the sample with the iterations was also reported, and damages to the sample were observed with time (SI-Figure S28) as well as differences in structure seen with ptychography (SI-Figure S28b). The remainder of the sample is shown in SI-Figure S28c with SEM analysis confirming the severe degradation of the sample.

In dry conditions, exposure to the beam was checked on other FIB-lamellae placed on the Cu grid. Repetition of scans was carried out locally, and no significant damage was observed in the Ca intensity (SI-Figures S29 and S30 for Loc 4 and Loc 2). DPC images were also reconstructed (SI-Figure S31). From the DPC images, changes in the structure were found on the FIB-lamella in the locations analyzed, as shown in the fifth iteration of beam exposure (SI-Figure S31). SAXS analysis was also carried out, and the SAXS patterns did not show major differences in scattering from the first to the fifth iteration (SI-Figure S30). SEM analysis was carried out, and modification of the structure was seen after the experiment (SI-Figures S32 and S33). This highlighted the importance to consider the influence of the X-ray beam on the sample after several exposures. Without liquid, damage could be identified after several repeated scans, which confirmed the importance to study further the X-ray interaction with the sample, to understand further the mechanism that occurs these in dry and wet conditions (not only radiolysis contributed to the damages as in the dry condition, some damages were seen). For example, beam-related damage could lead to amorphization of the sample and changes in the lattice and will require more investigation to delineate with the current study. SI-Figures S34 and 35 summarize each location analyzed before and after X-ray analysis with modifications found in the structure of the enamel.

### Structure Analysis—Soft and Hard X-ray Ptychography and DPC

**Figure 5:**
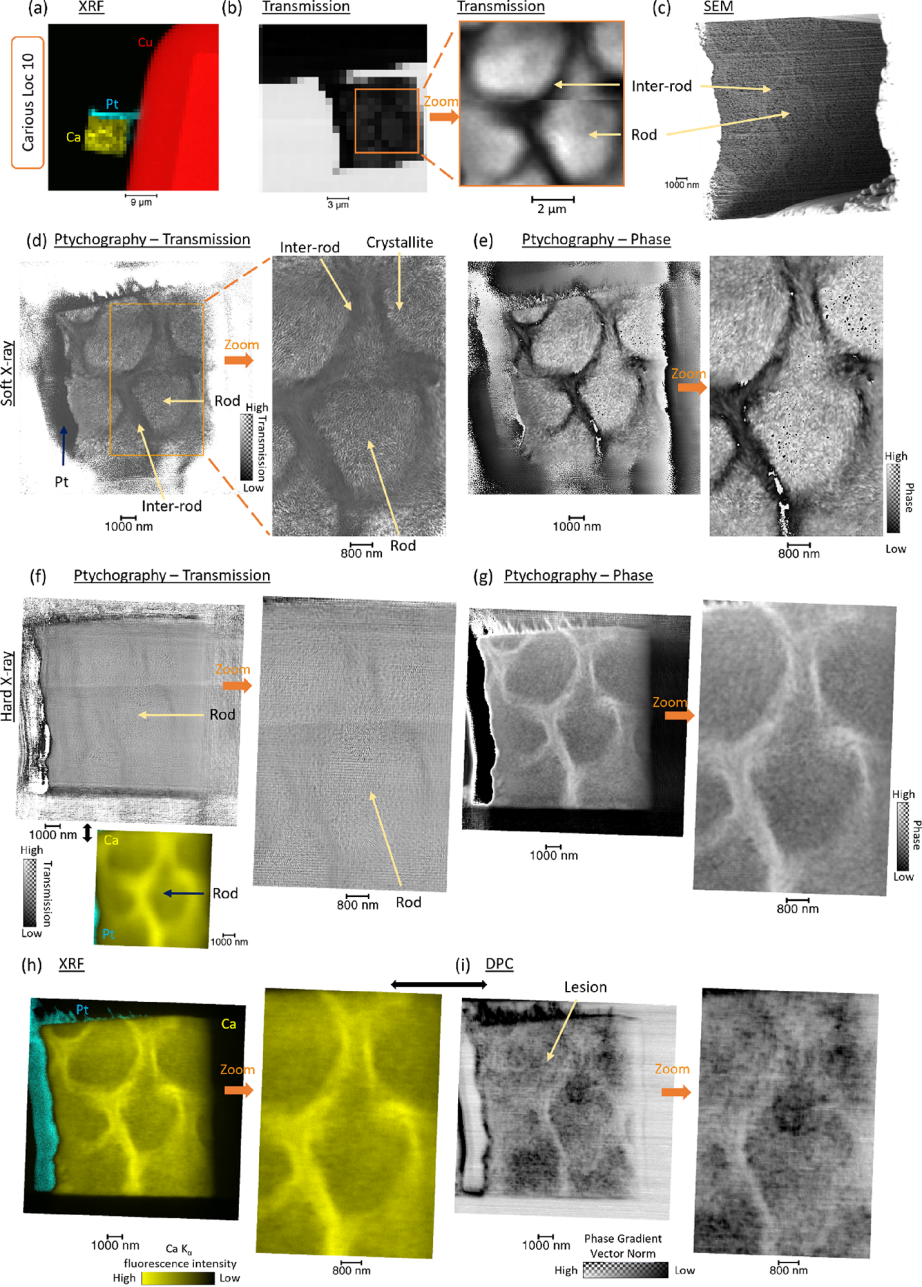
Nanocharacterization of Loc 10 in the carious region with ptychography, DPC, and XRF. (a) Hard X-ray XRF map at a low resolution (step 1000 nm) of Loc 10 with the details of Ca, Pt, and Cu fluorescence intensity. (b) STXM analysis and the sum of the intensity of the diffraction patterns from each location of the maps acquired with visualization of the rods and inter-rods in I08-1 (DLS, U.K.). (c) SEM image of the Loc 10, adapted from ref (4). (d) Ptychography analysis in I08 (DLS) with the transmission image and the details of the microstructure but also the identification of nanofeatures, the 2D ptychography reconstruction was performed with a pixel size of 8 nm, zoomed in ROI highlighted (e) corresponding to the phase recovered with zoom in. (f) Ptychography with the transmission image using hard X-rays in I14 (DLS, U.K.) and the simultaneous XRF data of Ca and Pt fluorescence intensity, the 2D ptychography reconstruction was performed with a pixel size of 23 nm (g) the corresponding phase. (h) XRF of Ca and Pt fluorescence intensity and DPC in (i) were simultaneously acquired in I14 (DLS, U.K.) with a beam size of ∼50 nm^2^. The correlation of the XRF and ptychography was illustrated with the superimposition of images from both mode and profile of lines extracted. The changes in the structure of rods and inter-rods follow the variations seen with the XRF data (SI-Figure S10).

To visualize the crystallite organization, soft X-ray transmission ptychography was carried out in I08-1 (J08) (DLS) (coherent lens-less mode); see the Methods Section for the experimental details. Loc 10 (carious region) was initially analyzed at a low resolution covering the Cu grid and sample. From the transmission details of the diffraction patterns (sum of the intensity of each pattern), the rod shape was identified and correlated to the XRF dataset and SEM image of the same FIB-lamella (Figure 5a–c). Ptychography was performed on the FIB-lamella in the same region where the scanning transmission X-ray microscope (STXM) overview was done. The sum of the diffraction patterns from transmission showed rod and inter-rod structures with localized variations of intensity. However, there was a lack of internal nanostructure (Figure 5b). The ptychography results are summarized in Figure 5d. The image revealed important details in enamel with structural variation between rods and inter-rods (Figure 5d,e), adding details on other dental tissue compared from a previous study on a dentine (resolution 158 nm^28^). The scale of the nanostructure observed was in line with the dimension of the crystallites. The pathway of the nanostructures was assessed with a clear distinction between the core of the rods and the inter-rods. The features seen were correlated with the imaging mode. From the SEM, the rod and inter-rod regions matched, with more details in the inter-rod region revealed with ptychography with the distinction of the pathway of the crystal in the samples. In terms of chemistry, the intense region of Ca found with XRF matched at the nanoscale with the regions reconstructed with ptychography, being on the boundary of the rods (SI-Figure S36 with the addition of SEM images). In the analysis of the data from both modalities, significant differences were found between rod and inter-rod regions, the Ca intensity was lower in the rods, and for the transmission, the standard deviation of the data in the rods was higher, as suggested from the variation of orientations (SI-Figure S36b). The correlative analysis added information that could not be obtained by using only one technique. It can also be seen that the core was less dense than the inter-rod, which correlated with the SEM image and the changes in composition. This was also correlated with the SAXS data and variation of orientation. The phase reconstructed also highlighted variations in the structure of the FIB-lamella in rod and inter-rod regions (Figure 5e).

The inhomogeneity in the distribution of the crystallites in enamel, in rods and inter-rods, has been previously reported.^4,21,58^ However, these studies either analyzed a few nm at the surface (requiring a fine polishing step) or required thin samples (∼100 nm). Here, the technique applied covers a large dimension, around 2 μm, leads to transmission and phase details, and can be potentially extended to 3D to reveal the 3D rod pathways and the internal porosity of carious enamel. This technique can be correlated to the dichroism technique, which has revealed the orientation information as well as the XANES spectra.^59^

The findings in Figure 5 suggested that the crystallite structure was reconstructed with details about the transmission and phase, and the method can be potentially transferred to other materials and can be extended to 3D ptychography^28^ to reveal the 3D hidden organization of the crystallites and preferential demineralization regions.

Although the resolution obtained was low, APT and TEM still provided better resolution but the volume and thickness of the sample commonly analyzed are smaller.^60−62^ From our knowledge of the studies on human enamel and our previous work,^16^ ptychography has emerged as an important technique in the characterization of materials with a hierarchical structure down to the nanoscale. In other samples that were analyzed, less scattering and transmission were noticed, which led to limited reconstruction (SI-Figure S37). The other FIB-lamellae were in the non-carious region (thickness ∼0.5 μm lower than Loc 10) and transition enamel (thickness 2 μm higher than Loc 10) with less demineralization than Loc 10. It was suggested that it was better to have a thin sample or with demineralized regions, as seen in the carious FIB-lamella, for the visualization of details in enamel by ptychography.

The soft X-ray ptychography results were correlated with those attained using hard X-ray ptychography (performed on beamline I14 at DLS). Loc 10 was characterized by an energy of 8 keV. The transmission data revealed the crystallite structure but with limitations compared to soft X-ray analysis (Figure 5e). The possibility to observe the structure was also confirmed in another carious location Loc 9. The reconstructed phase showed the rods, following the XRF data results. For the thicker FIB-lamella in the carious and transition zones (Loc 11 and 13), there was a weak variation of signal and observation of the structure, which was not observed using the soft X-ray. Non-carious enamel with smaller thicknesses (down to 0.56 μm for Loc 2, SI2-Table S1) were also studied, and the sheath region could be visualized without a clear distinction of the crystallites. For the surface enamel, the sheath region was detectable. The results are summarized in SI-Figures S38 and S39. Ptychography was carried out simultaneously with XRF (using a larger beam sizer in comparison to WAXS or SAXS) using the flexibility of the beamline. It was important to consider that these were 2D results and were influenced by the sample thickness, but they provided a first step before 3D ptychography. However, these techniques lack the resolution that can be obtained with TEM, but they have not been fully optimized yet and have other advantages (ability to measure thicker samples and larger fields of view and offers the possibility for 3D measurements and *in situ* analysis). The analysis/results are complementary. Preliminary TEM analysis carried out on a FIB-lamella from a carious region was able to identify crystallites, with the demineralization noticed within the core region of the crystallites. In addition to the crystallite shapes and demineralized region, the lattice was seen in some locations, providing a level of detail likely to provide additional insights into caries (SI-Figure S40). However, similar to the adverse effects caused by repeated exposure to X-rays, sample damage was observed (SI-Figure S40c), as previously reported.^63^

In addition to ptychography, differential phase contrast (DPC) imaging was also carried out with a step of 50 nm simultaneously to the XRF in I14 (DLS) with the benefit of co-registration with a similar resolution for both techniques. Different phase contrast retrieval data correlated well with the SEM images with the observation of sheath region for instance in the surface region, a differentiation in the structure in rods and inter-rods. By comparing carious and non-carious regions, differences were found, with the observation of the rods and inter-rods in the carious FIB-lamellae (SI-Figures S10, S12, and S41) and higher standard deviation in the data from the carious region (SI-Figure S41a). In the carious region, significant differences were found between rods and inter-rods with higher values in inter-rods (SI-Figure S41b). Local variations of contrast in correlation with the demineralized regions were seen on SEM and were also visualized with XRF. The DPC technique reveals the internal structures of enamel, which are hidden by the redeposition occurring during the FIB milling. The DPC imaging technique provided the rapid high-resolution images of the sample with XRF images being acquired simultaneously, (Figure 5g,f and SI-Figures S10 and S12–S16) and highlighted the location of the sheath region.

The analyses contributed to the correlative method in the analysis of enamel and are summarized in SI-Table S1. The method could also be transferred to dentine^17^ and be extended to provide an analysis in 3D, which is required in the analysis of enamel; as mentioned by Boyde, “the main problem in studying enamel is that its structure must be seen in three dimensions”.^64^ It can also allow the visualization of cracks or pores in the material from the variation of refractive index in the structure, revealing details in soft tissue that transmission imaging could not detect.^65^ Using the correlative analysis, the results from the diffractions mapping could be correlated with DPC (SI-Figures S14–S17), providing structure and phase details at nanoscale resolution. Global correlative analyses were carried out, and they are summarized in SI-Figure S12. As the analysis that was carried out generated 2D images, further analyses are required to overcome the effects of averaging the data over the thickness of the sample. As reported previously, the mechanical properties of enamel are excellent, and based on the analyses reported, correlations with the mechanical properties can be suggested for further studies, which can provided insights into the interpretations of the mechanical results.^66^

## Conclusions

In summary, we proposed a thorough approach for applying correlative chemical and imaging modalities to analyze (carious) enamel at nanoscales from the preparation of samples to the analysis of the acquired data. Using this approach, soft X-ray ptychography was successfully applied to visualize the organization of the crystallites, which revealed (subject to sample thickness and structure) the inhomogeneity of the crystallite pathways. We also showed that DPC can be applied to perform a structural analysis and the visualization of the sheath and pores based on the variation of phase contrast of the structures.

We also demonstrated the variation of the elemental composition, diffraction, and scattering pattern down to a step and beam size of around 55–72 nm^2^ using XRF, combined with WAXS and SAXS, respectively. Using XRF, Ca Kα fluorescence intensity variation was found higher in the carious region than in other locations. In the inter-rods of the carious region, a boundary region with higher Ca Kα intensity was seen concurring with the SEM analysis, showing the requirement of nanoresolution to probe these changes and preferential directions. Here, we show the feasibility of chemical analysis of enamel in various regions with maps using a beam size of ∼50 nm^2^. Such an approach provided localized details of the composition in enamel and the differences seen in carious enamel down to the rod and inter-rod level and correlated with SEM imaging. In addition, the flexibility of the beamline analyses leads to the possibility of acquiring diffraction data together with other imaging modalities, which were investigated here.

The crystallography changes from rods to inter-rods with variations of peak intensity were inferred owing to the high-resolution and localized acquired X-ray diffraction patterns. The variation in the crystal lattice can be extended to measure nanostrain, size, and further texture analysis, which adds details to other nanoscale techniques such as APT, polarization-dependent imaging contrast.

From SAXS, variation of the scattering could be identified, and changes in the alignment were detected in the carious and surface enamel in comparison to the non-carious sample. During the nanoprobe analysis, the material degradation under X-ray exposure was detected and required further analysis.

The proposed nanoscale correlative platform provides wealth of information that can be used to inform and inspire further enamel studies, including, for example, a large number of samples for pathological samples, 3D analyses, and *in situ* investigations. This technical platform is also expected to be informative for studying materials other than enamel in medical applications and to continue the development of correlative technique.

## Data Availability

Data collected and interpreted in this study is maintained by the authors and can be made available upon request.

## Supporting Information Available

Details of the SAXS pattern in several locations of Loc 10; XRF map of Ca intensity with the locations of the SAXS pattern shown; additional details on Loc 10 in Figure 4 (Movie S1) (MPG)
Additional experimental details, results, including several figures of experimental results supporting the manuscript and a table summarizing the techniques (PDF)
Additional experimental details, materials, and methods, including figures and photography of the experiment setups (PDF)

## Supplementary Material

am3c08031_si_001.mpg
