# Supplementary material for: Multi-resolution Correlative Ultrastructural and Chemical Analysis of Carious Enamel by Scanning Microscopy and Tomographic Imaging
Source: ACS Appl Mater Interfaces. 2023 Jul 31;15(31):37259–73. doi: 10.1021/acsami.3c08031 (PMC10416148; doi:10.1021/acsami.3c08031)
Supplement: Supplementary file 2 — am3c08031_si_002.pdf [file am3c08031_si_002.pdf]

Multi-resolution Correlative Ultrastructural and Chemical Analysis of Carious Enamel by Scanning Microscopy and Tomographic Imaging

Authors

Cyril Besnard*[a], Ali Marie[a], Sisini Sasidharan[a,1], Petr Buček[b], Jessica M. Walker[c], Julia E. Parker[c], Matthew C. Spink[c], Robert A. Harper[d], Shashidhara Marathe[c], Kaz Wanelik[c], Thomas E.J. Moxham[a,c], Enrico Salvati[a,2], Konstantin Ignatyev[c], Michał M. Kłosowski[e], Richard M. Shelton[d], Gabriel Landini[d], Alexander M. Korsunsky[a*]

[a] MBLEM, Department of Engineering Science, University of Oxford, Parks Road, Oxford, Oxfordshire, OX1 3PJ, U.K.

[b] TESCAN-UK Ltd., Wellbrook Court, Girton, Cambridge CB3 0NA, U.K.

[c] Diamond Light Source Ltd., Didcot, Oxfordshire, OX11 0DE, U.K.

[d] School of Dentistry, University of Birmingham, 5 Mill Pool Way, Edgbaston, Birmingham, West Midlands, B5 7EG, U.K.

[e] Research Complex at Harwell, Harwell Campus, OX11 0FA, U.K.

[1] Present address: Department of Materials, Imperial College London, SW7 London, U.K.

[2] Present address: Polytechnic Department of Engineering and Architecture, University of Udine, 33100 Udine UD, Italy

Email addresses:

cyril.besnard@eng.ox.ac.uk, ali.marie@eng.ox.ac.uk, sisini.sasidharan@eng.ox.ac.uk, petr.bucek@tescan.com, jessica.walker@diamond.ac.uk, julia.parker@diamond.ac.uk, matthew.spink@diamond.ac.uk, R.A.Harper@bham.ac.uk, shashidhara.marathe@diamond.ac.uk, kaz.wanelik@diamond.ac.uk, thomas.moxham@eng.ox.ac.uk, enrico.salvati@eng.ox.ac.uk, konstantin.ignatyev@diamond.ac.uk, michal.klosowski@rc-harwell.ac.uk, R.M.Shelton@bham.ac.uk, G.Landini@bham.ac.uk, alexander.korsunsky@eng.ox.ac.uk

* ***Corresponding authors***: Cyril Besnard, cyril.besnard@eng.ox.ac.uk

Alexander M. Korsunsky, alexander.korsunsky@eng.ox.ac.uk

Supporting Information (SI)

Table of Contents

Figures

Supporting Fig. S1.	pp. 3-4	Supporting Fig. S21.	pp. 37-38
Supporting Fig. S2.	pp. 5-6	Supporting Fig. S22.	pp. 39-40
Supporting Fig. S3.	pp. 7-8	Supporting Fig. S23.	pp. 41-42
Supporting Fig. S4.	p. 8	Supporting Fig. S24.	p. 42
Supporting Fig. S5.	pp. 9-10	Supporting Fig. S25.	pp. 43-44
Supporting Fig. S6.	p. 11	Supporting Fig. S26.	pp. 45-46
Supporting Fig. S7.	pp. 12-13	Supporting Fig. S27.	pp. 47-48
Supporting Fig. S8.	pp. 14-15	Supporting Fig. S28.	pp. 49-50
Supporting Fig. S9.	pp. 16-17	Supporting Fig. S29.	pp. 51-52
Supporting Fig. S10.	pp. 18-19	Supporting Fig. S30.	pp. 53-54
Supporting Fig. S11.	p. 20	Supporting Fig. S31.	pp. 55-56
Supporting Fig. S12.	p. 21	Supporting Fig. S32.	pp. 57-58
Supporting Fig. S13.	pp. 22-23	Supporting Fig. S33.	pp. 59-60
Supporting Fig. S14.	p. 24	Supporting Fig. S34.	pp. 61-62
Supporting Fig. S15.	pp. 25-26	Supporting Fig. S35.	p. 62
Supporting Fig. S16.	pp. 27-28	Supporting Fig. S36.	pp. 63-64
Supporting Fig. S17.	pp. 29-30	Supporting Fig. S37.	p. 65
Supporting Fig. S18.	pp. 31-32	Supporting Fig. S38.	pp. 66-67
Supporting Fig. S19.	pp. 33-34	Supporting Fig. S39.	pp. 68-69
Supporting Fig. S20.	pp. 35-36	Supporting Fig. S40.	pp. 70-71
		Supporting Fig. S41.	p. 72

Table

SI-Table S1. pp. 73-74

Movie

Movie S1. p. 74

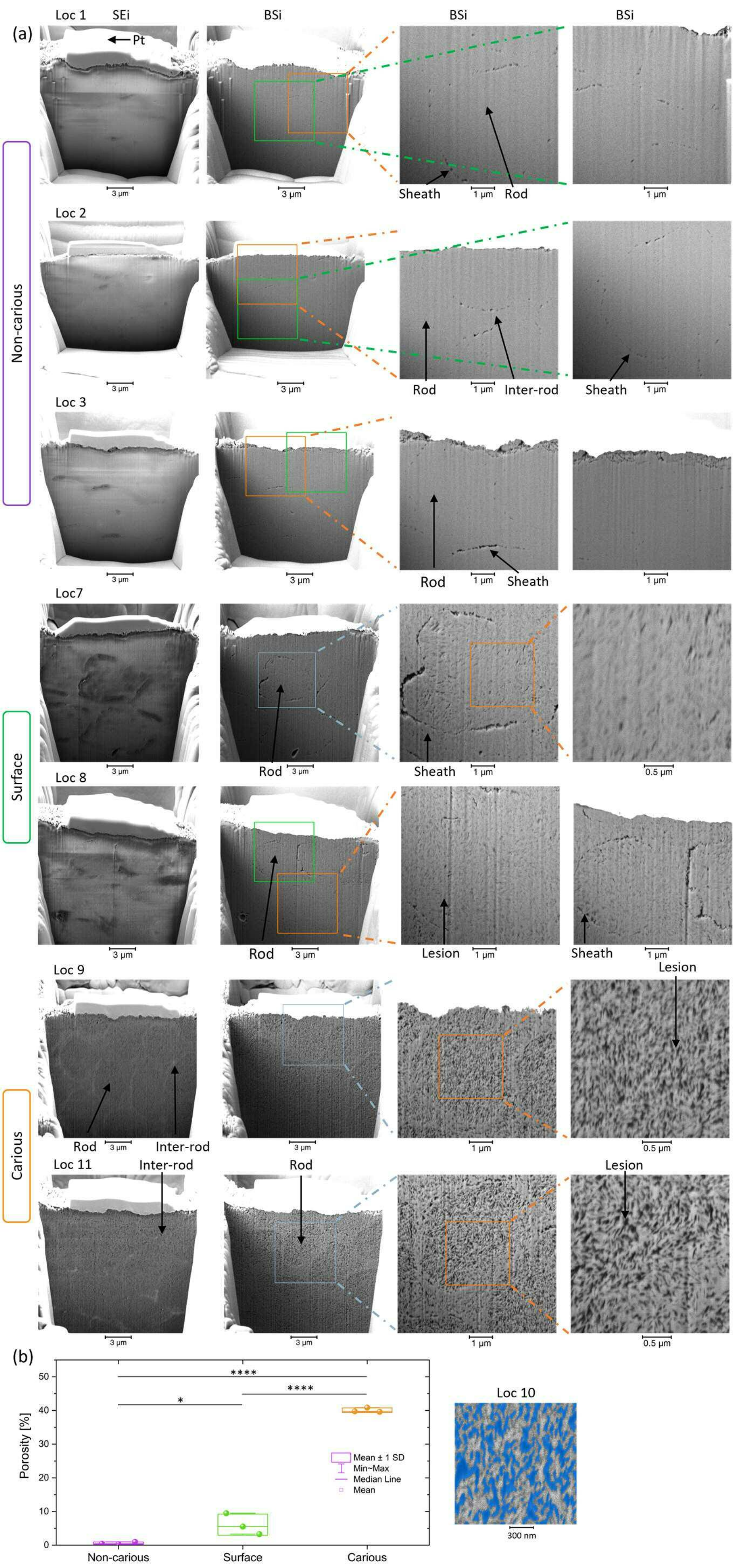

(a)
Loc 1
SEi
BSi
BSi
BSi
Pt
Sheath
Rod
Loc 2
Rod
Inter-rod
Sheath
Loc 3
Rod
Sheath
Loc7
Rod
Sheath
Loc 8
Rod
Lesion
Sheath
Lesion
Loc 9
Rod
Inter-rod
Loc 11
Inter-rod
Rod
Lesion
Non-carious
Surface
Carious
3 μm
1 μm
0.5 μm
(b)
Porosity [%]
50
40
30
20
10
0
****
****
*
Mean ± 1 SD
Min~Max
Median Line
Mean
Non-carious
Surface
Carious
Loc 10
300 nm

SI-Fig S1. Summary of the scanning electron microscopy (SEM) images of several locations after thinning the cross-sections using a focused ion beam (FIB). Secondary and backscattered electron images (SEi and BSi) of locations Loc 1,2,3,7,8,9 and 11 with zooming in regions of the cross-section highlighted differences in the porosity as a function of location (carious, surface zone and non-carious region). Clear alteration of the structure visualised in the carious region. For the images of the other locations analysed in the manuscript, see[1]. For the locations of the milling carried out on the enamel sample, see Figure 1. Platinum (Pt) highlighted on the surface. (b) Statistical analysis of the porosity in the regions of enamel (230 × 230 pixels for Loc 2,1,3,8,7,6,11,9,10), one-way ANOVA test with post hoc Tukey's test was carried out. * represents $p < 0.05$ and **** represents $p \leq 0.0001$. Image of the segmented data of the Loc 10.

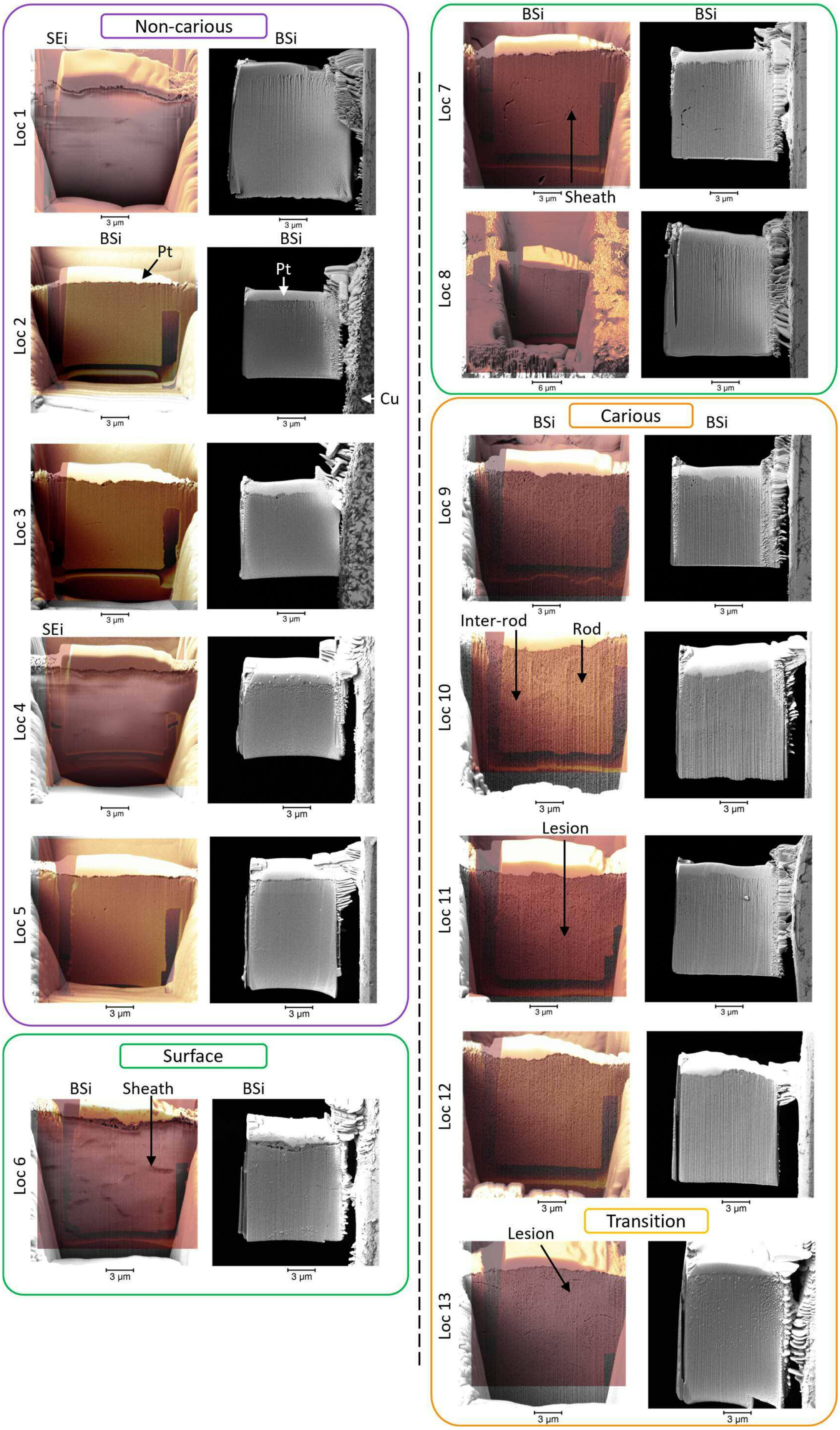

Non-carious
SEi
BSi
Loc 1
3 µm
BSi
Pt
Loc 2
Cu
Loc 3
SEi
Loc 4
Loc 5
Surface
BSi
Sheath
Loc 6
Loc 7
Loc 8
6 µm
Carious
Loc 9
Inter-rod
Rod
Loc 10
Lesion
Loc 11
Loc 12
Transition
Loc 13

SI-Fig. S2. SEM images of the cross-sections of the locations analysed before and after lifted out the FIB-lamella. Superimposition of the BSi of the cross-sections after FIB thinning preparation and the BSi after undercut, and BSi of the FIB-lamellae after lifted out and attached to the copper (Cu) grid. Differences in the structure of the enamel were observed from the cross-sections.

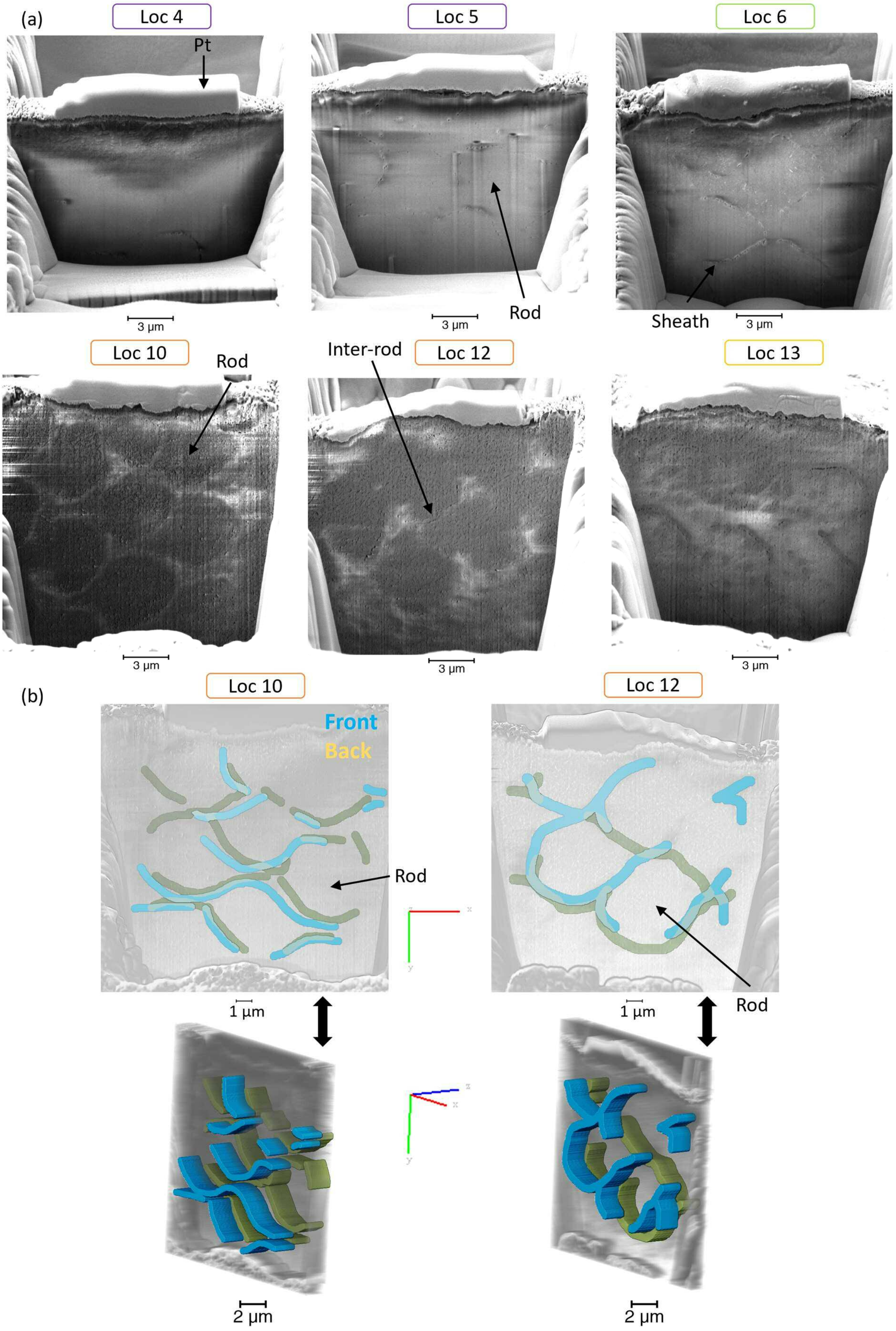
(a)
Loc 4
Pt
3 µm
Loc 5
Rod
3 µm
Loc 6
Sheath
3 µm
Loc 10
Rod
3 µm
Inter-rod
Loc 12
3 µm
Loc 13
3 µm
(b)
Loc 10
Front
Back
Rod
1 µm
2 µm
Loc 12
Rod
1 µm
2 µm

SI-Fig. S3. SEM images of the cross-sections from the front and back view. (a) SEM images of view of the back of the cross-sections, SEi. (b) Manual alignment of the two SEM images of the front and the back view was carried out and combined to have a stack of two images. SEM images filtered with Gaussian filter (2D) and segmentation was carried out using the Segmentation editor in Avizo. The shape of the rods was extracted based on the features seen on the SEM images. Loc 10 and Loc 12 in the carious region are shown.

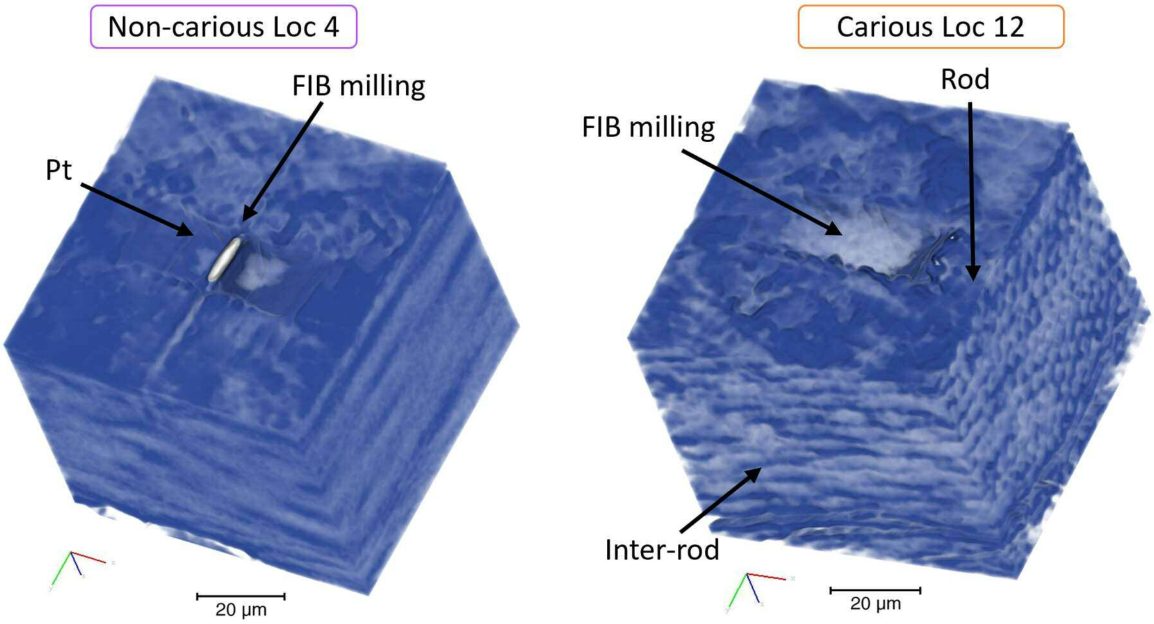

SI-Fig. S4. Synchrotron X-ray tomography of a location in the non-carious region Loc 4 and one in the carious region Loc 12. 3D representation (after 3D median filtered) of Loc 4 and Loc 12 with the visualisation of the shape of the rods in the carious region, voxel size of 0.325 µm.

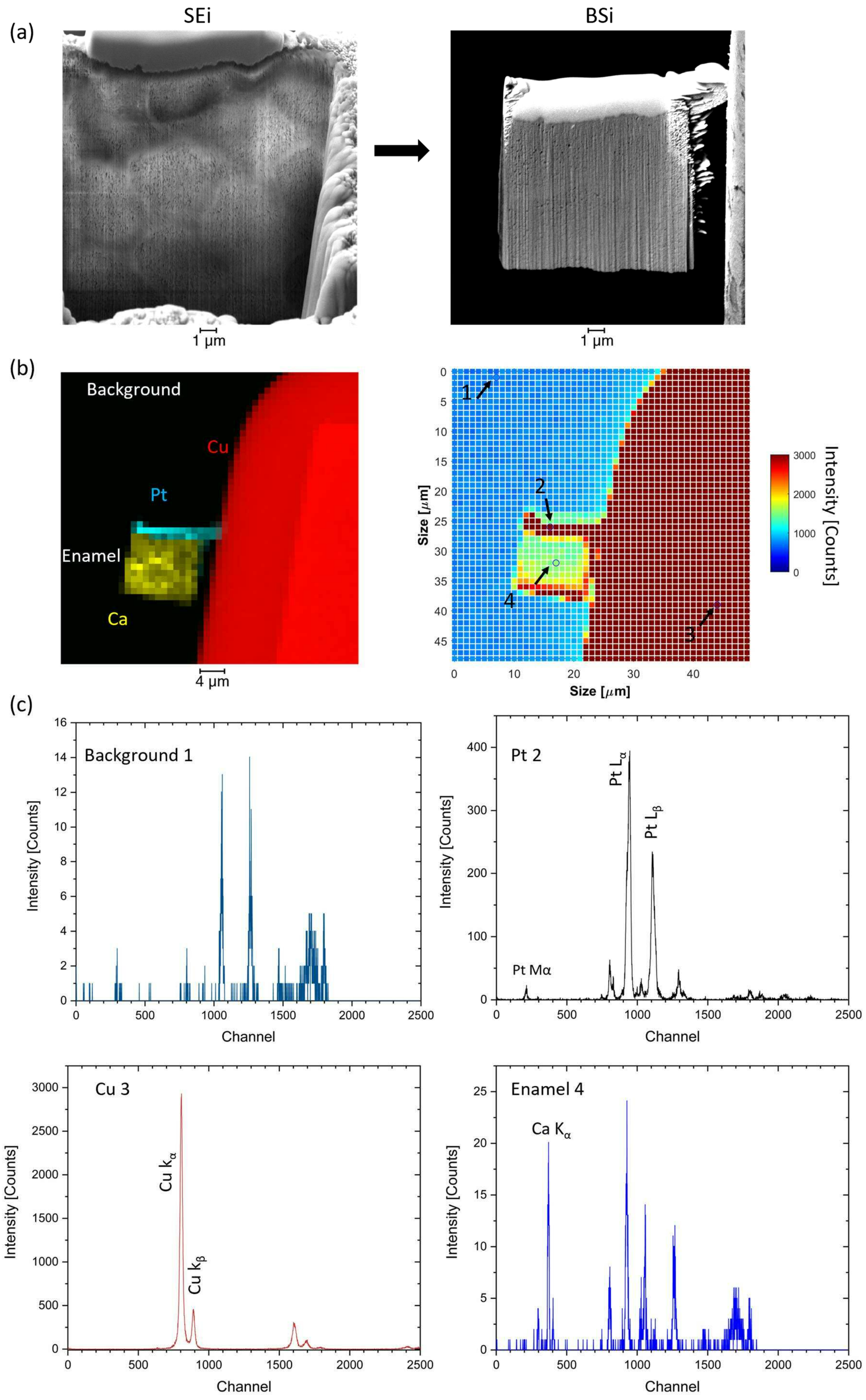
(a)
SEi
BSi
1 µm
1 µm
(b)
Background
Cu
Pt
Enamel
Ca
4 µm
1
2
3
4
Size [µm]
Size [µm]
Intensity [Counts]
(c)
Background 1
Intensity [Counts]
Channel
Pt 2
Pt L_α
Pt L_β
Pt Mα
Cu 3
Cu k_α
Cu k_β
Enamel 4
Ca K_α

SI-Fig. S5. SEM image of Loc 10 and the details of the X-ray fluorescence spectroscopy (XRF) map with a resolution of 150 nm. (a) SEM images of the cross-section and FIB-lamella of Loc 10. (b) Overview of the signal from the FIB-lamella with a superimposition of the XRF signal of calcium (Ca), platinum (Pt), and Cu, and the highlight of four locations. (c) XRF spectra of the four locations, an acquisition time of 15 ms, background, platinum deposition, copper grid and enamel (with also the contribution of the instrumental background and scattering from Pt layer). Each channel was 10 eV.

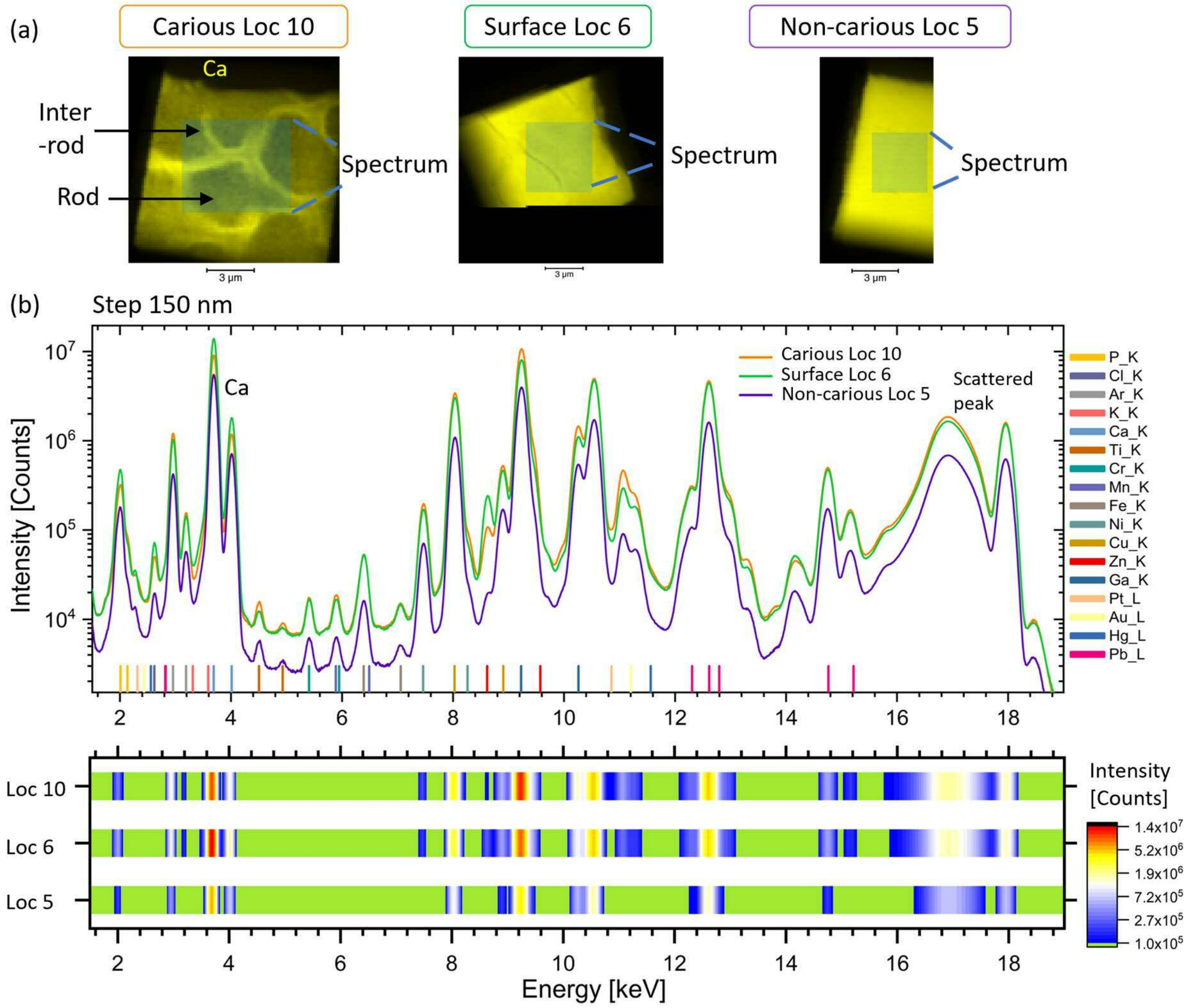

SI-Fig. S6. Details of the XRF spectral analysis of several regions of the enamel (carious, non-carious and surface) with step of 150 nm. (a) Location of the regions where the spectra plot in Figure 1a were extracted, for Loc 10,6,5, step of 150 nm, energy 18 keV (beam size of 40 × 57 nm^2 in focus for carious and non-carious regions, and 71 × 62 nm^2 in focus for the surface region prior to moving the stage by 75 µm for the analyses). (b) Plot of the spectrum of the sum of the intensity of the fluorescence signal of all the pixels in the region of interest of Loc 10, Loc 6 and Loc 5 in (a) using 18 keV X-rays (see Figure 1a for additional details). Several elements detected with signal from sample, setup, and processing (e.g. gallium (Ga)).

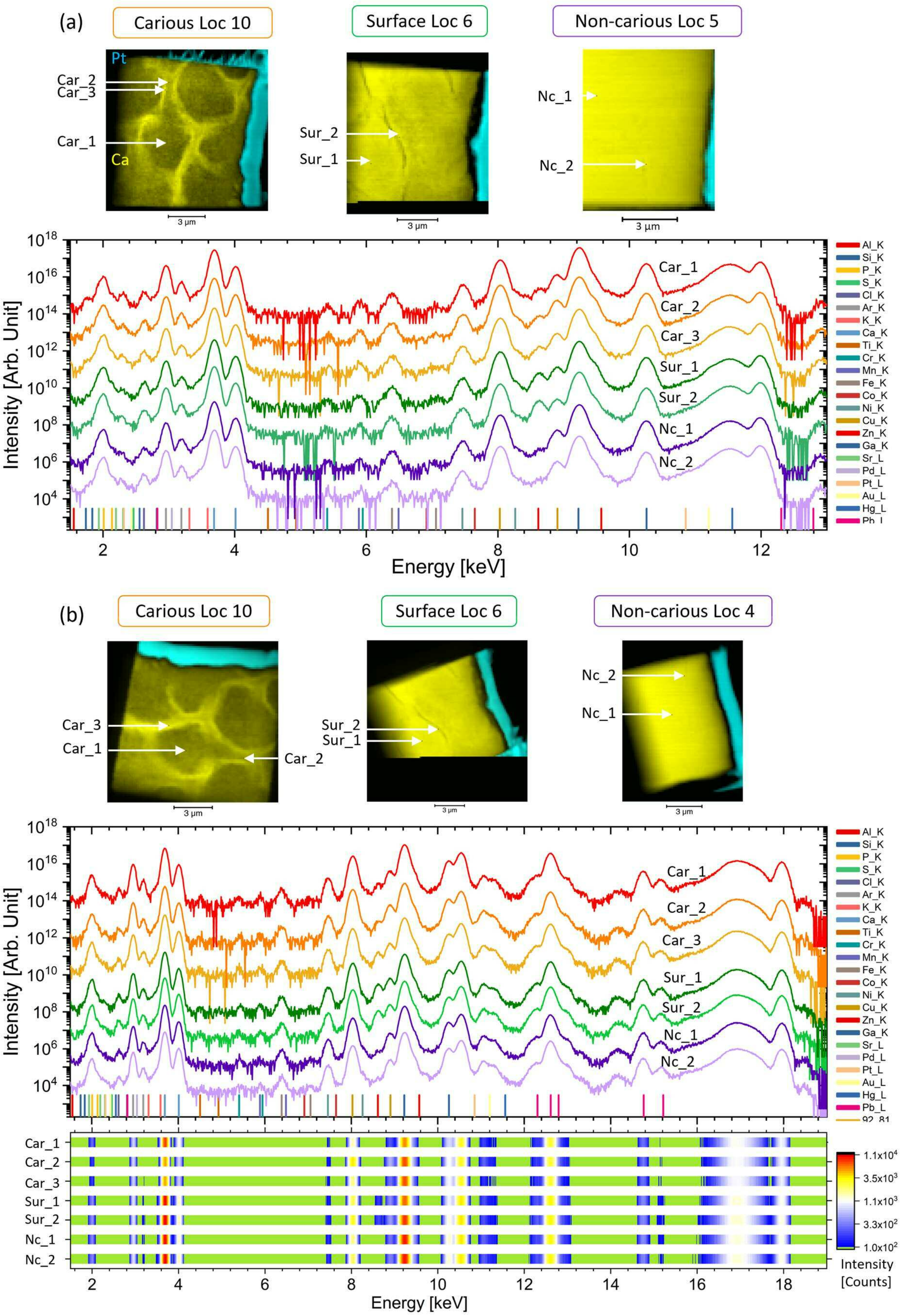

SI-Fig. S7. Details of the XRF spectral analysis of several regions of the enamel with step of 150 nm. (a) XRF map with Ca and Pt fluorescence intensity with the locations of the spectra plotted, energy

used 12 keV, step of 150 nm and from the Loc 10,6,5 (beam size of 55 × 45 nm^2 in focus prior to moving the stage by 75 µm for the analyses). (b) XRF map of Ca and Pt with the position of the spectra plot in the figure (b), energy used 18 keV, step of 150 nm from the Loc 10,6,4 (beam size of 40 × 57 nm^2 in focus for carious and 71 × 62 nm^2 in focus for surface and non-carious regions prior to moving the stage by 75 µm for the analyses). In the figure the abbreviation Car, Sur and Nc referred to as carious, surface and non-carious enamel with at least two pixels analysed in each locations referred to as _1 and _2. The region of interests were acquired with exposure of 5 s.

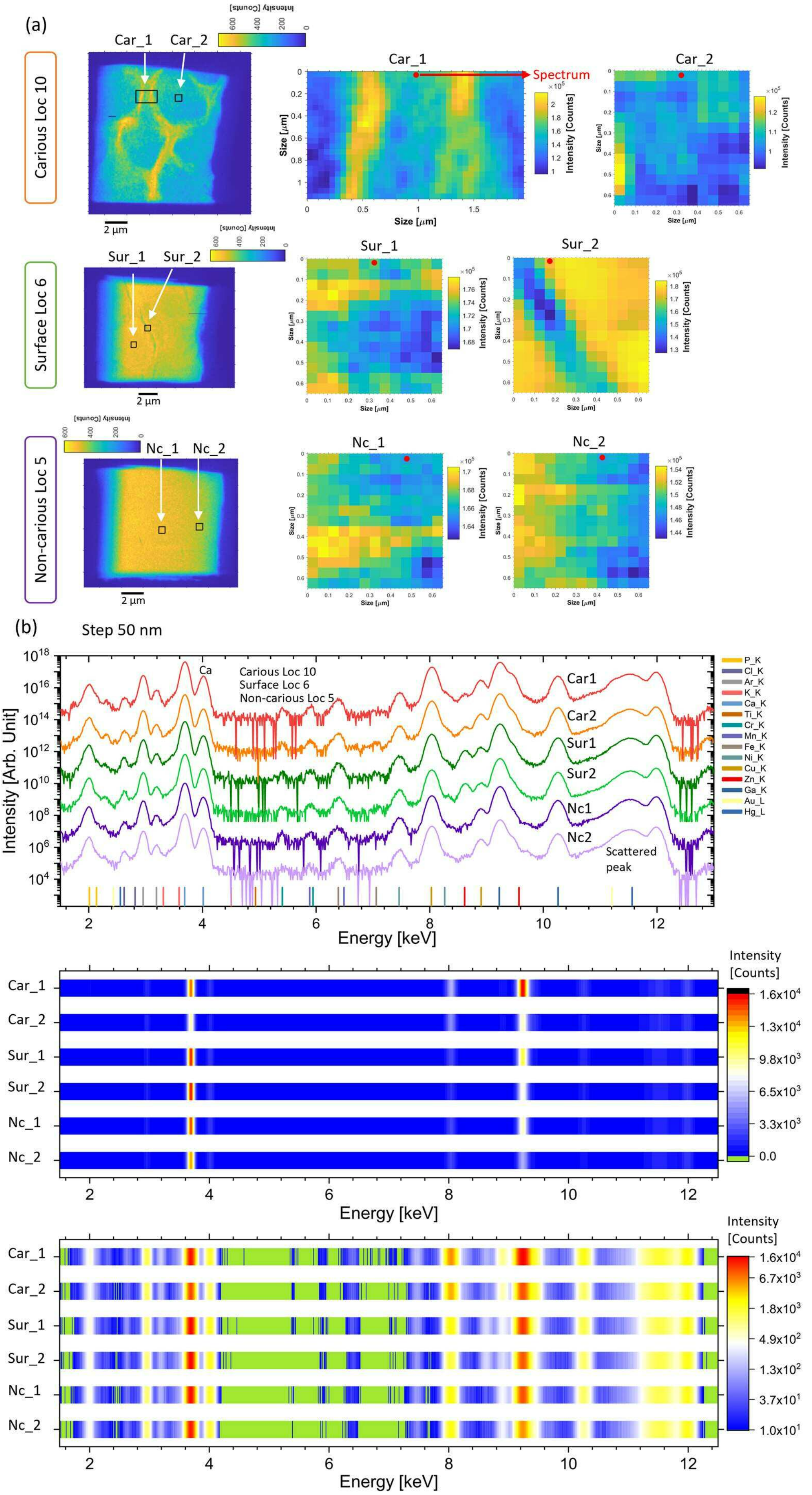
(a)
Carious Loc 10
Car_1
Car_2
Intensity [Counts]
2 µm
Spectrum
Size [µm]
Surface Loc 6
Sur_1
Sur_2
Non-carious Loc 5
Nc_1
Nc_2
(b)
Step 50 nm
Intensity [Arb. Unit]
Ca
Carious Loc 10
Surface Loc 6
Non-carious Loc 5
Car1
Car2
Sur1
Sur2
Nc1
Nc2
Scattered peak
P_K
Cl_K
Ar_K
K_K
Ca_K
Ti_K
Cr_K
Mn_K
Fe_K
Ni_K
Cu_K
Zn_K
Ga_K
Au_L
Hg_L
Energy [keV]
Intensity [Counts]

SI-Fig. S8. Details of the XRF spectral analysis of several regions of the enamel with a step of 50 nm. (a) XRF map of Ca with a large dimension and with the location of the regions where the spectra plotted in Figure 1b were extracted, for Loc 5, Loc 6 and Loc 10, with a step of 50 nm, energy 12 keV and beam size of 55×45 nm^2. The regions of interest were acquired with exposure of 5 s. In the figure the abbreviations Car, Sur and Nc are referred to as carious, surface and non-carious enamel, respectively, with at least two pixels analysed in each _1 and _2. (b) Plots of the XRF spectra in the various locations in addition to the details in Figure 2.

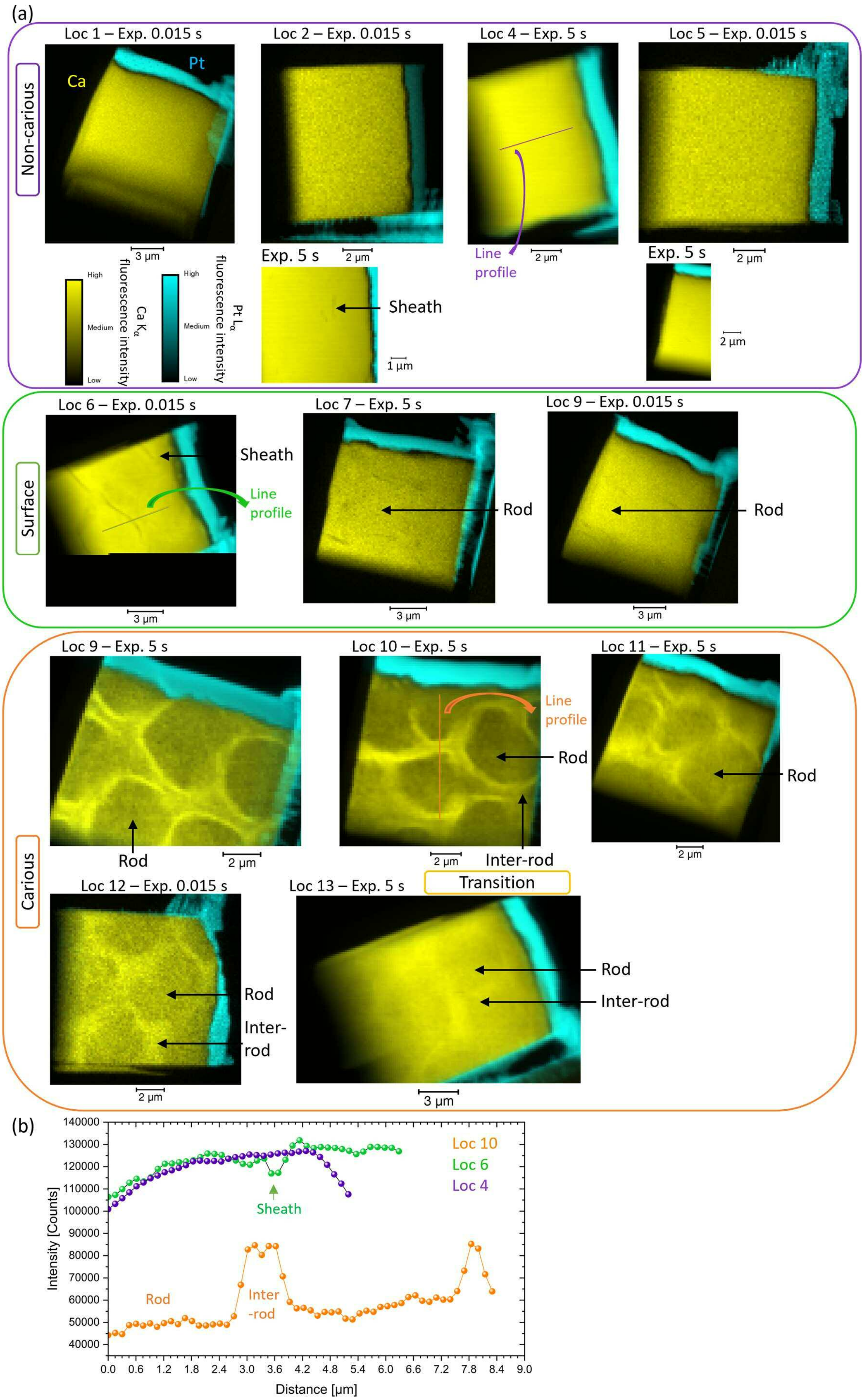

(a)
Non-carious
Loc 1 – Exp. 0.015 s
Ca
Pt
3 µm
Loc 2 – Exp. 0.015 s
Exp. 5 s
2 µm
Sheath
1 µm
Loc 4 – Exp. 5 s
Line profile
2 µm
Loc 5 – Exp. 0.015 s
Exp. 5 s
2 µm
2 µm
High
Medium
Low
Ca Kα fluorescence intensity
High
Medium
Low
Pt Lα fluorescence intensity
Surface
Loc 6 – Exp. 0.015 s
Sheath
Line profile
3 µm
Loc 7 – Exp. 5 s
Rod
3 µm
Loc 9 – Exp. 0.015 s
Rod
3 µm
Carious
Loc 9 – Exp. 5 s
Rod
2 µm
Loc 10 – Exp. 5 s
Line profile
Rod
Inter-rod
2 µm
Loc 11 – Exp. 5 s
Rod
2 µm
Transition
Loc 12 – Exp. 0.015 s
Rod
Inter-rod
2 µm
Loc 13 – Exp. 5 s
Rod
Inter-rod
3 µm
(b)
Intensity [Counts]
Distance [µm]
Loc 10
Loc 6
Loc 4
Sheath
Rod
Inter -rod
140000
130000
120000
110000
100000
90000
80000
70000
60000
50000
40000
0.0 0.6 1.2 1.8 2.4 3.0 3.6 4.2 4.8 5.4 6.0 6.6 7.2 7.8 8.4 9.0

SI-Fig. S9. XRF spectroscopy Ca map of all the locations analysed with a step size of 150 nm (unfocus). (a) Description of the XRF Ca map for locations in non-carious, surface, carious and transition zone with also the intensity for Pt to delimitate the FIB-lamella position, a step of 150 nm and scan exposure (exp.) reported. (b) A plot of the line profile extracted from three locations, Loc 4 non-carious, Loc 10 carious and Loc 6 surface zone, line profile analysis with a step of 150 nm. For the non-carious Loc 2 and Loc 5 additional maps are illustrated with exposure (Exp.) of 5 s with the view of the sheath revealed.

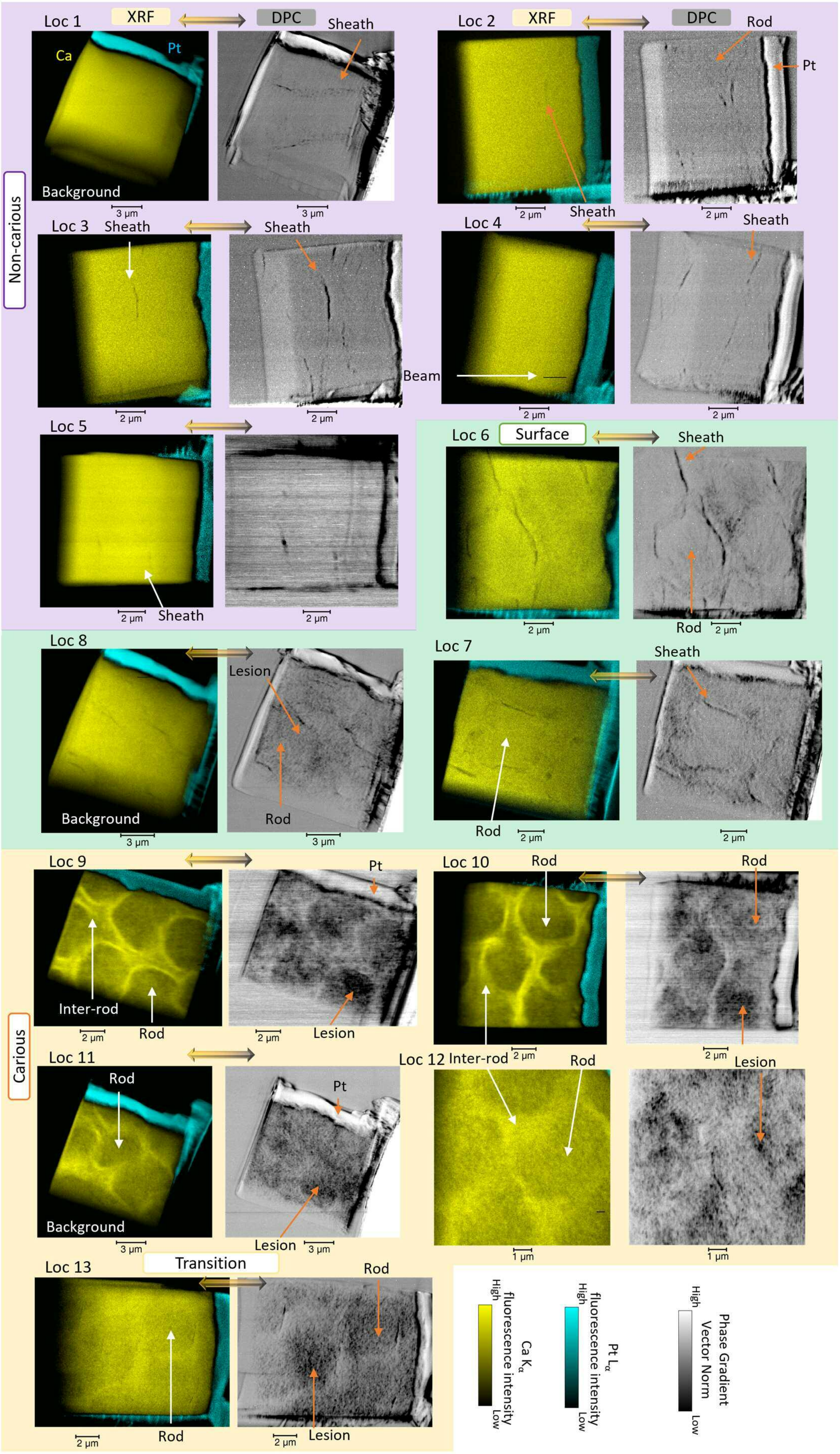
Loc 1
XRF
DPC
Sheath
Ca
Pt
Background
3 μm
3 μm
Loc 2
XRF
DPC
Rod
Pt
2 μm
Sheath
2 μm
Sheath
Non-carious
Loc 3
Sheath
Sheath
2 μm
2 μm
Loc 4
Beam
2 μm
2 μm
Loc 5
Sheath
2 μm
2 μm
Loc 6
Surface
Sheath
Rod
2 μm
2 μm
Loc 8
Lesion
Background
Rod
3 μm
3 μm
Loc 7
Sheath
Rod
2 μm
2 μm
Loc 9
Pt
Inter-rod
Rod
Lesion
2 μm
2 μm
Loc 10
Rod
Rod
Lesion
2 μm
2 μm
Carious
Loc 11
Rod
Pt
Background
Lesion
3 μm
3 μm
Loc 12 Inter-rod
Rod
Lesion
1 μm
1 μm
Loc 13
Transition
Rod
Rod
Lesion
2 μm
2 μm
High
Low
Ca Kα fluorescence intensity
High
Low
Pt Lα fluorescence intensity
High
Low
Phase Gradient Vector Norm

SI-Fig. S10. XRF of Ca and Pt intensity map and corresponding differential phase contrast (DPC) images for several locations described in the Figure 1 with a step of 50 nm (in focus). Correlative modal analysis with chemistry and phase contrast of several locations with a step of 50 nm (in focus). XRF map with Ca intensity and Pt (to highlight the orientation and position of the FIB-lamella), with a clear variation of the fluorescence signal and contrast between the carious region to the other regions in the enamel. Corresponding DPC imaging of the same region acquired simultaneously showing the variation of contrast in the structure of enamel. The analysis was done either with 8 or 12 keV and exposure of 0.015 s.

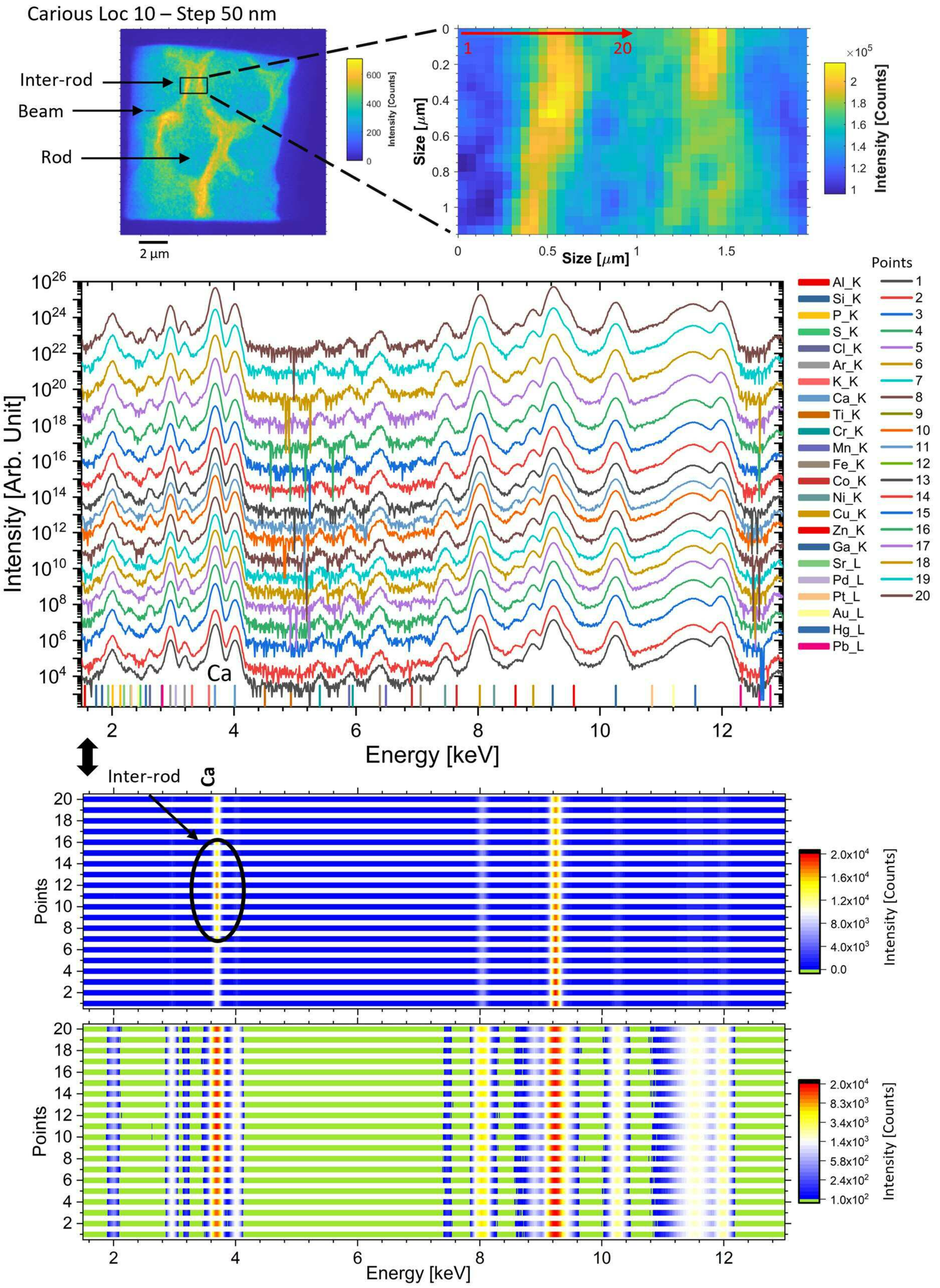

SI-Fig. S11. Localised analysis of the XRF analysis with a step of 50 nm (in focus) on Loc 10 for example. XRF map of Ca K_α fluorescence intensity with zoom in one location at the inter-rod, line scan plotted with a step of 50 nm, energy used 12 keV. The scan exposure of the region of interest was 5 s and for the large map 0.015 s, beam size of 55 × 45 nm^2.

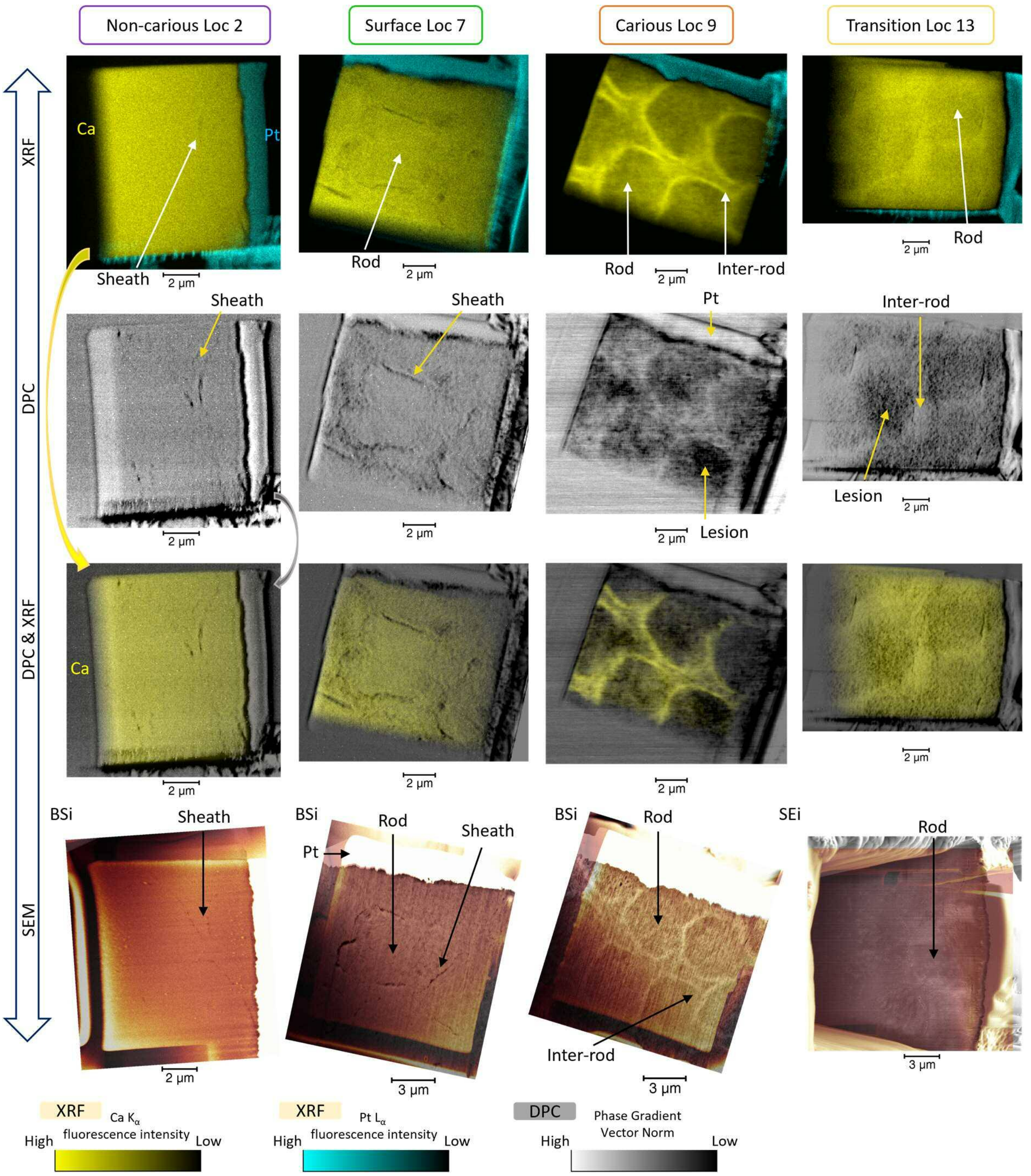

SI-Fig. S12. Overview of the correlative and imaging mode analysis for Loc 2,7,9,13. XRF map of Ca and Pt, XRF analysis with combined DPC, SEM images of the FIB-lamella superimposed to the SEM images of the cross-section of Loc 9 in carious, Loc 7 in surface and Loc 2 in non-carious and Loc 13 in the transition zone respectively. Significant differences were seen in structure and chemistry in these locations. Superimposition of images highlighting the correlative analysis carried out.

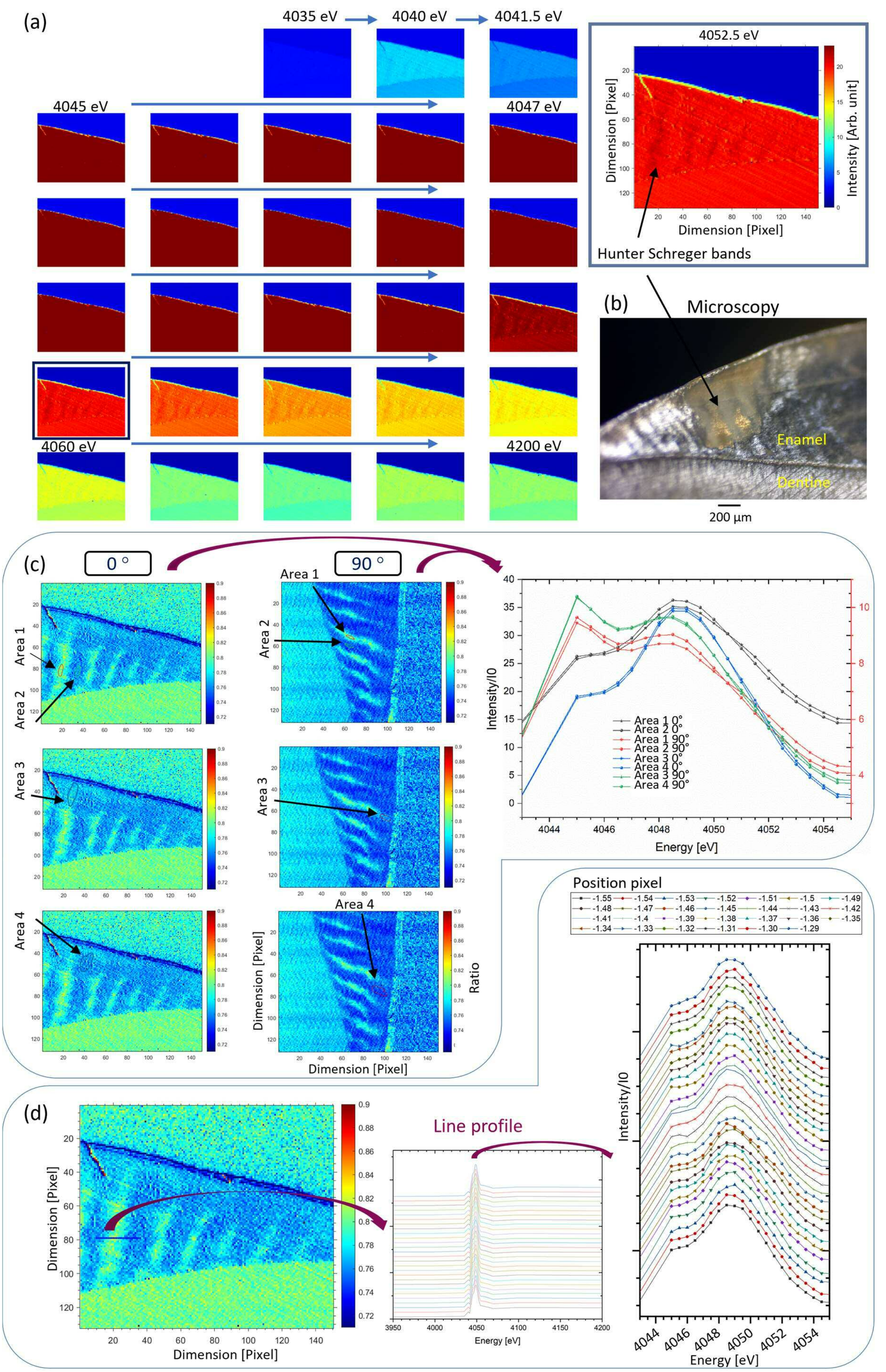
(a)
4035 eV
4040 eV
4041.5 eV
4045 eV
4047 eV
4060 eV
4200 eV
4052.5 eV
Dimension [Pixel]
Intensity [Arb. unit]
Hunter Schreger bands
(b)
Microscopy
Enamel
Dentine
200 µm
(c)
0 °
90 °
Area 1
Area 2
Area 3
Area 4
Ratio
Intensity/I0
Energy [eV]
Area 1 0°
Area 2 0°
Area 1 90°
Area 2 90°
Area 3 0°
Area 4 0°
Area 3 90°
Area 4 90°
Position pixel
(d)
Line profile
Energy [eV]

SI-Fig. S13. Synchrotron micro-XRF analysis with XANES in I18 (DLS). (a) X-ray absorption near edge structure (XANES) analysis of the slice for one rotation angle with the maps of the sample at different energy following the range of energies described in the method. A highlight of one energy 4052.5 eV with the visualisation of the enamel structure and Hunter-Schreger bands. (b) Light microscopy image of the slice before the synchrotron analysis. (c) Map from the ratio of the dataset at energy 4051 eV and 4048.5 eV (both divided by I0) at two rotation angles (0 and 90 °). Regions of interest in these maps shown, which were located in different regions of the enamel and the plot of the sum of the intensity of each pixel in these regions highlighted for the energy around 4048 eV showed the variation in the intensity. (d) Line profile with a step of one pixel extracted from the enamel based on the map in (c). Plot of the XANES spectra for each pixel along the line and zoom in the region around 4048 eV.

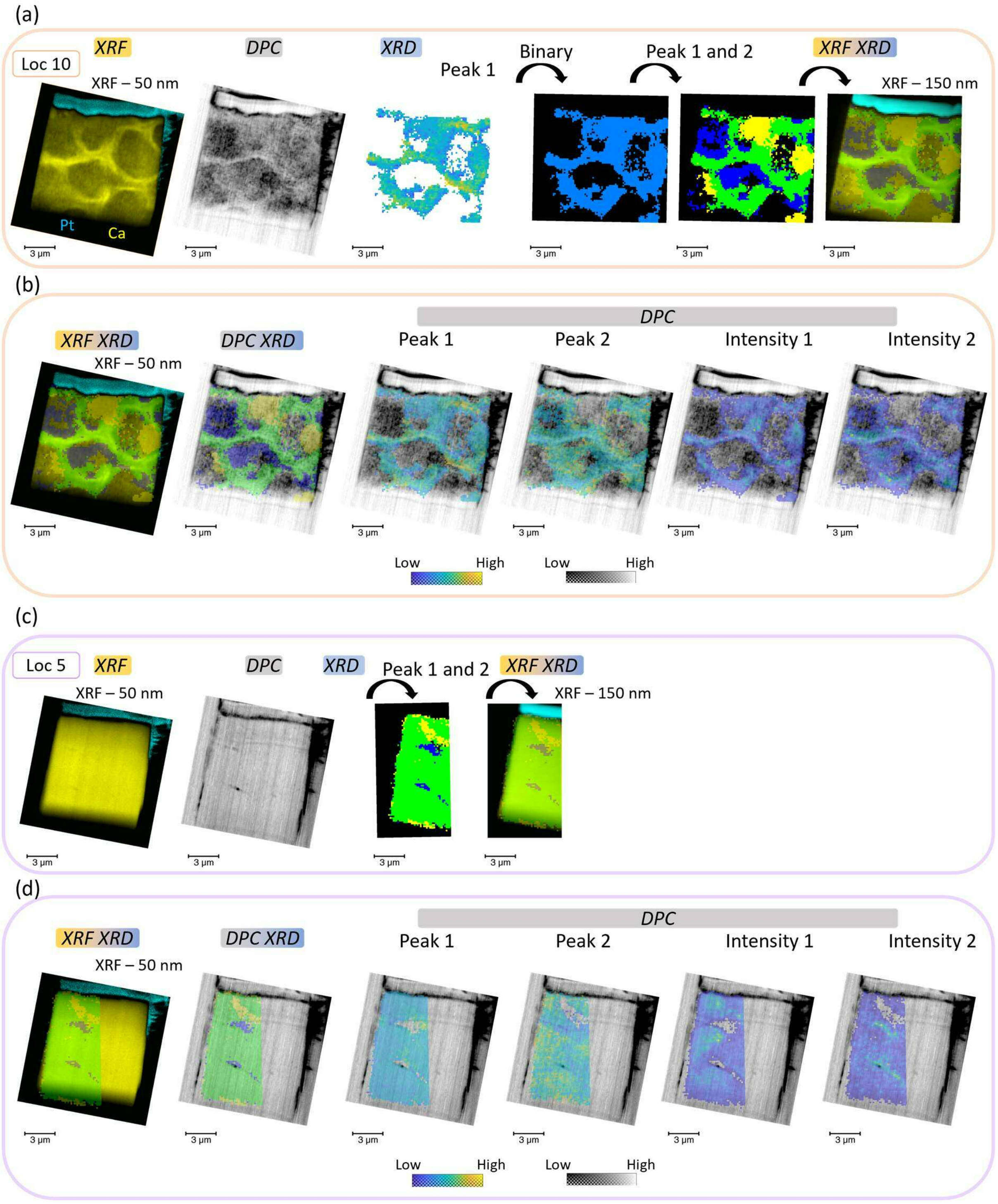

SI-Fig. S14. XRF and WAXS diffraction analysis of Loc 10 and Loc 4 for the correlation. (a) Analysis of Loc 10, XRF map of Ca intensity with a step of 50 nm, DPC image, XRD map with a step of 150 nm of the peak 1 position (position of the peak described in Figure 3), a binary image of this map, and then the map of the location of peak 1 in yellow, peak 2 in blue and the two peaks in green superimposed with XRF map with a step of 150 nm. (b) Superimposition of the map of the location of the peak on the XRF map with a step of 50 nm and on the DPC image. Superimposition of DPC image with individual map of the WAXS analysis (step of 150 nm), peak 1 and peak 2 with q value (see Figure 3 for more details) and intensity of the two peaks. (c,d) Analysis of Loc 5 based on (a,b) and Figure 3. Colour map showing the diffraction data and the DPC image.

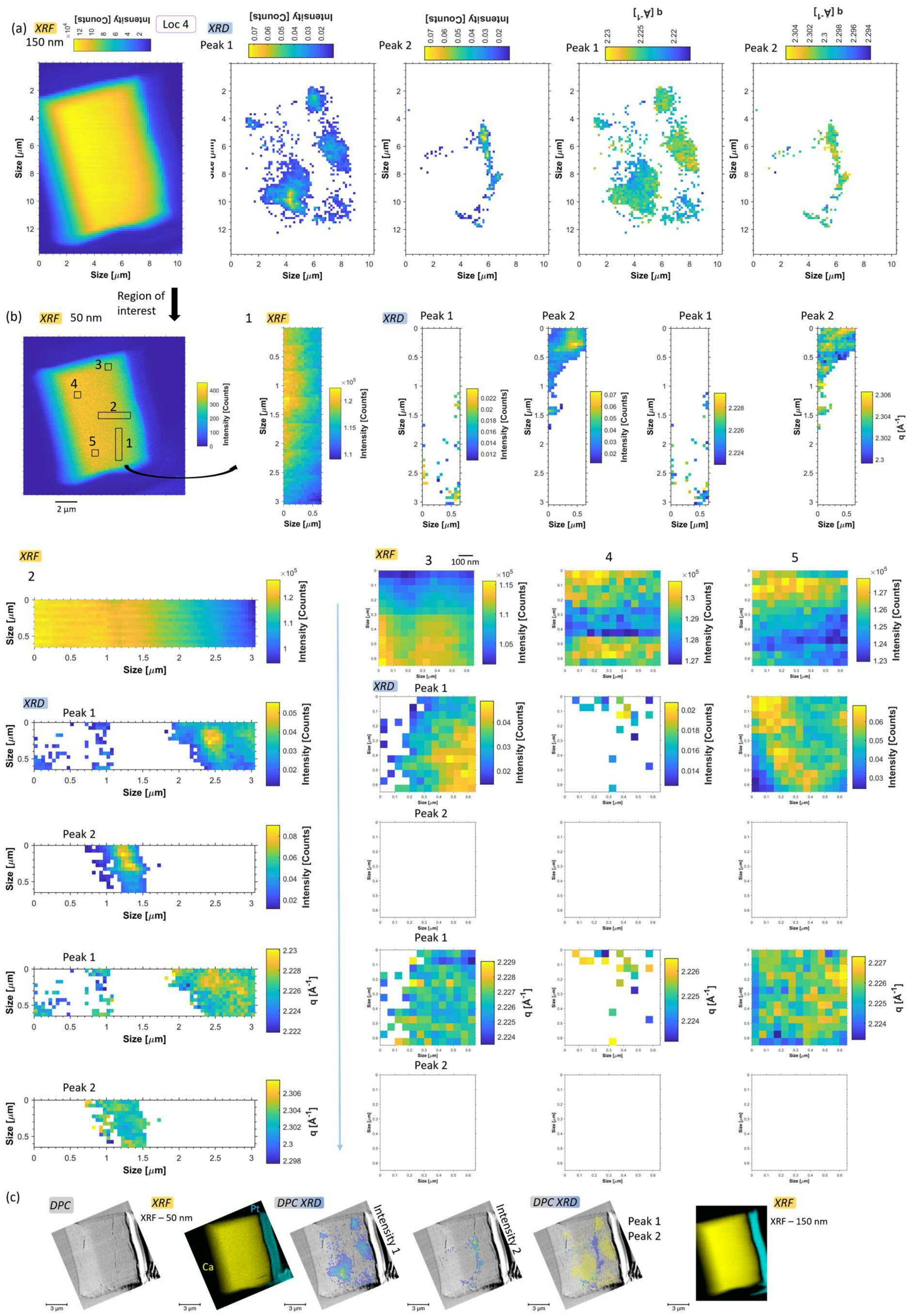

SI-Fig. S15. XRF and WAXS diffraction analysis of Loc 4 at low and high resolution. (a) XRF map of Ca with a step of 150 nm and the corresponding WAXS diffraction results for the peak 1 and 2, plane

respectively, intensity and peak position are illustrated with the map. (b) Analysis of the regions of interest (ROI) displayed on the Ca XRF map, XRF map of Ca with the ROI shown on the XRF Ca map with a step of 50 nm (in focus) and the WAXS results with intensity and peak position (q). (c) Correlative imaging analysis with the DPC image, XRF map of Ca and Pt (step of 50 nm), superimposition of the diffraction results of the peak 1 and 2 on the DPC results and superimposition of the diffraction results specifically to the location of peak 1 and 2 (see SI-Fig. S14 for the colour code and method) and XRF map of Ca and Pt with a step of 150 nm (scale bar 3 µm). The correlation of the structure of the enamel, rods and inter-rods visualised with DPC, to the SEM analysis referred to SI-Figs. S1,10 and Figure 1. Beam size of 71 × 62 nm^2 was used during the diffraction experiment when in focus.

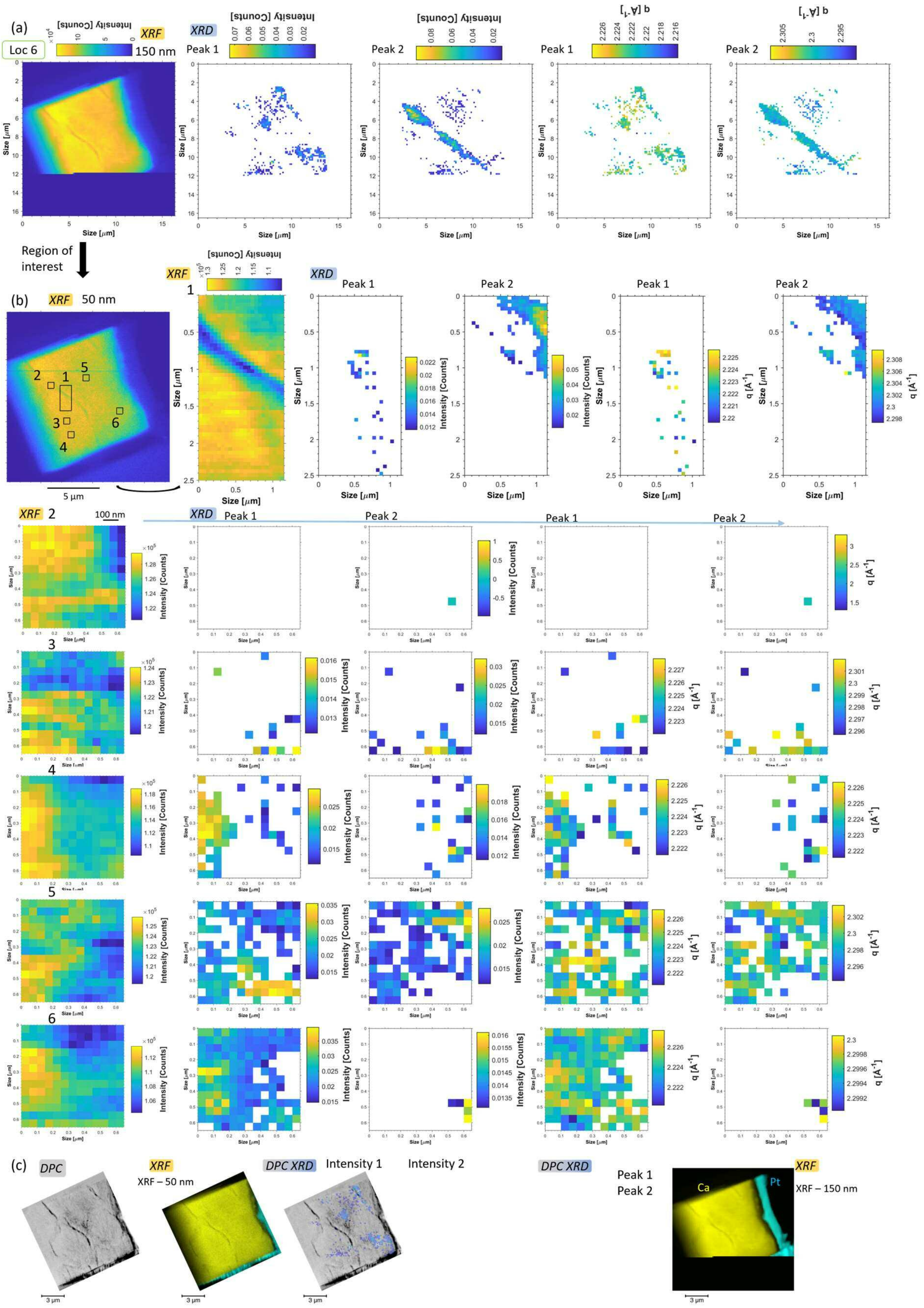

SI-Fig. S16. XRF and WAXS diffraction analysis of Loc 6 at low and high resolution. (a) XRF map of Ca with a step of 150 nm and the corresponding WAXS diffraction results for the peak 1 and 2, plane respectively, intensity and peak position are illustrated with the map. (b) Analysis of the regions of

interest (ROI) displayed on the Ca XRF map, XRF map of Ca with the ROI shown on the XRF Ca map with step of 50 nm (in focus) and the WAXS results with intensity and peak position (q). (c) Correlative imaging analysis with the DPC image, XRF map of Ca and Pt (step of 50 nm), superimposition of the diffraction results of the peak 1 and 2 on the DPC results and superimposition of the diffraction results specifically to the location of peak 1 and 2 (see SI-Fig. S14 for the colour code and method) and XRF map of Ca and Pt with a step of 150 nm (scale bar 3 µm). The correlation of the structure of the enamel, rods and inter-rods visualised with DPC, to the SEM analysis referred to SI-Figs. S1,10 and Figure 1. Beam size of 71 × 62 nm^2 was used during the diffraction experiment when in focus.

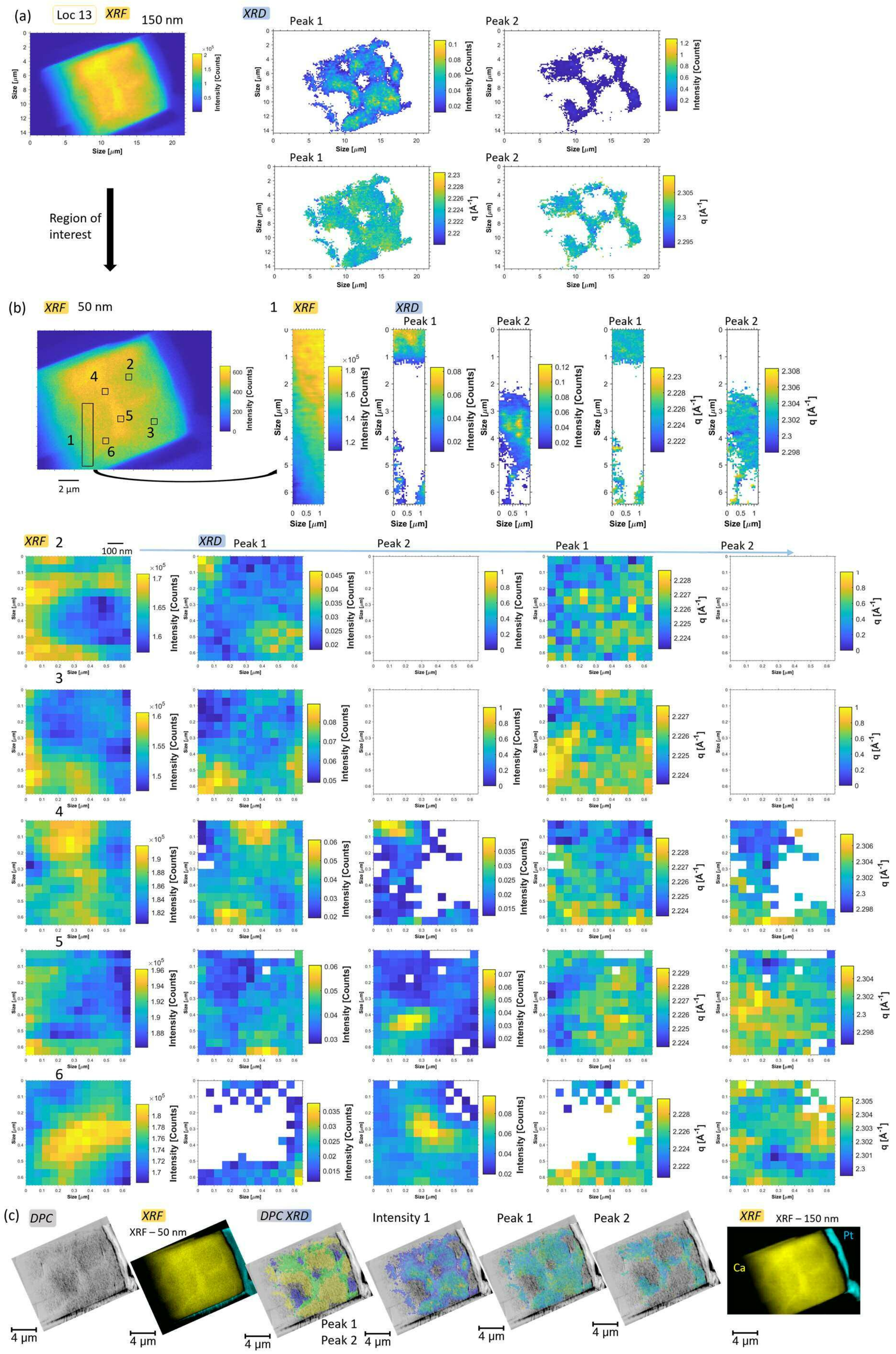
(a)
Loc 13
XRF
150 nm
XRD
Peak 1
Peak 2
Region of interest
(b)
XRF
50 nm
2 µm
100 nm
Intensity [Counts]
q [Å⁻¹]
Size [µm]
(c)
DPC
XRF
XRF – 50 nm
DPC XRD
Intensity 1
Peak 1
Peak 2
XRF – 150 nm
Pt
Ca
4 µm

SI-Fig. S17. XRF and WAXS diffraction analysis of Loc 13 at low and high resolution. (a) XRF map of Ca with a step of 150 nm and the corresponding WAXS diffraction results for the peak 1 and 2, plane respectively, intensity and peak position are illustrated with the map. (b) Analysis of the regions of interest (ROI) displayed on the Ca XRF map, XRF map of Ca with the ROI shown on the XRF Ca map with a step of 50 nm (in focus) and the WAXS results with intensity and peak position (q). (c) Correlative imaging analysis with the DPC image, XRF map of Ca and Pt (step of 50 nm), superimposition of the diffraction results of the peak 1 and 2 on the DPC results and superimposition of the diffraction results specifically to the location of peak 1 and 2 (see SI-Fig. S14 for the colour code and method) and XRF map of Ca and Pt with step of 150 nm (scale bar 3 µm). The correlation of the structure of the enamel, rods and inter-rods visualised with DPC, to the SEM analysis referred to SI-Figs. S1,10 and Figure 1. Beam size of 71 × 62 nm^2 was during the diffraction experiment when in focus.

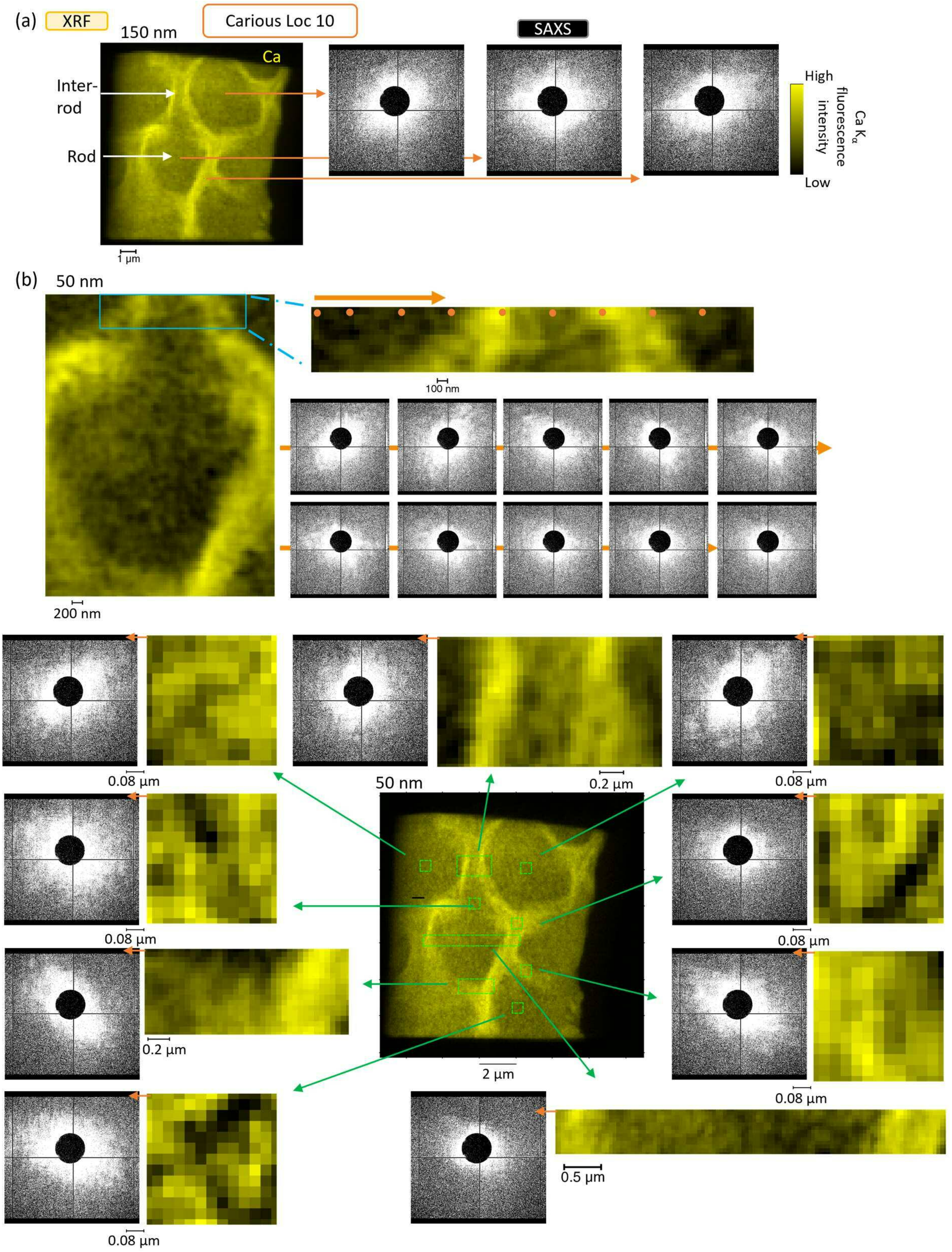

SI-Fig. S18. XRF/SAXS analysis of Loc 10 in the carious region. (a) XRF/SAXS analysis of Loc 10 with a step of 150 nm, exposure of 5 s, Ca K_α fluorescence intensity map and SAXS patterns from different regions with the observation of the scattering (beam size was 55 × 45 nm^2 in focus before moving the stage by 75 µm for the analyses). (b) XRF/SAXS analysis of Loc 10 in carious with a step of 50 nm. XRF map of Ca K_α fluorescence intensity with a step of 50 nm and an exposure of 5 s and the highlight of a region of interest extracted with the XRF map plotted and extracted SAXS patterns from various pixels (orange dot), going through rod and inter-rod as well as other regions of interest

with the XRF map and one SAXS pattern illustrated per region. Beam size was 55 × 45 nm^2 for the analyses.

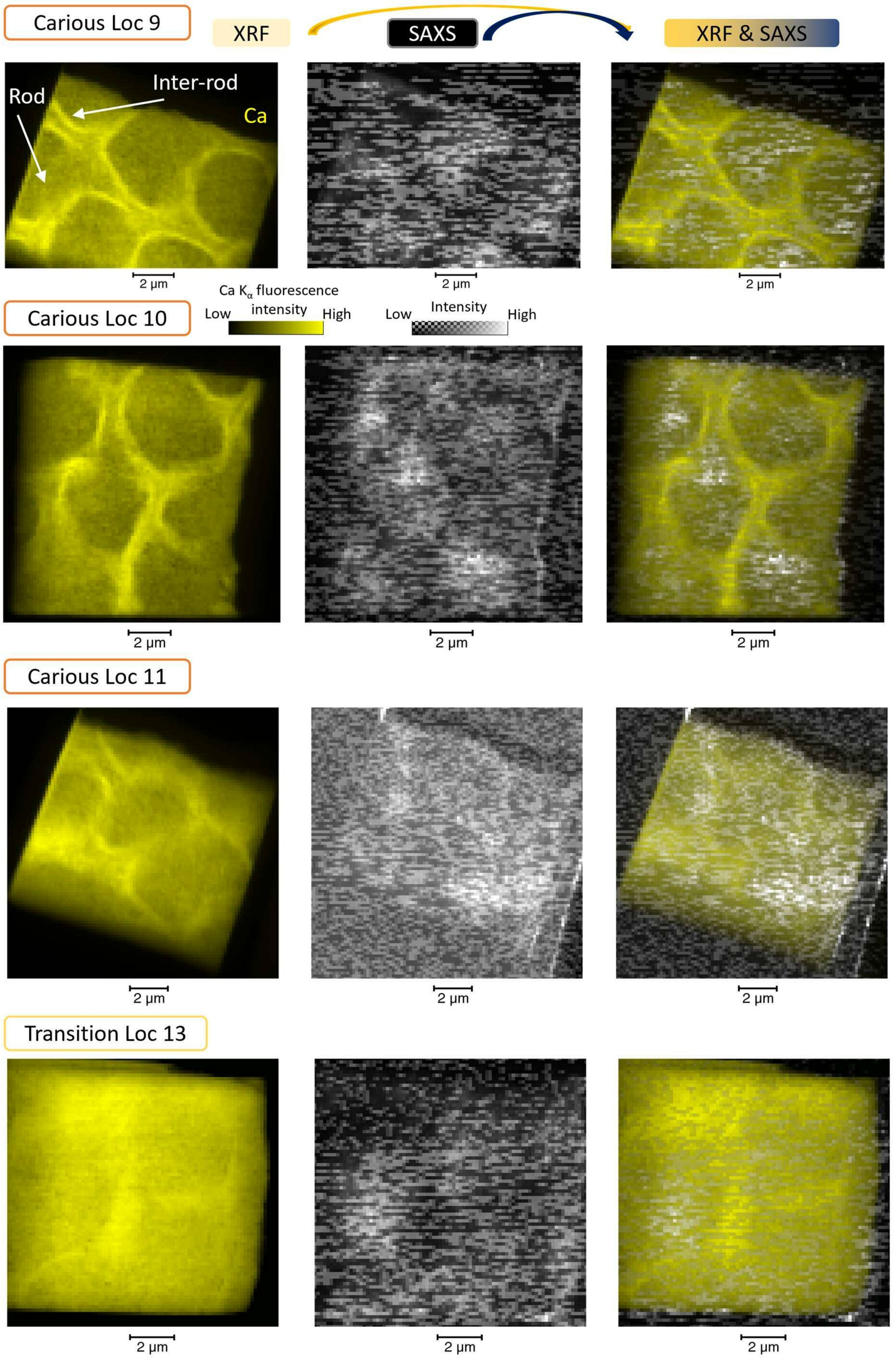
Carious Loc 9
XRF
SAXS
XRF & SAXS
Rod
Inter-rod
Ca
2 µm
2 µm
2 µm
Ca K_α fluorescence intensity
Low
High
Low
Intensity
High
Carious Loc 10
2 µm
2 µm
2 µm
Carious Loc 11
2 µm
2 µm
2 µm
Transition Loc 13
2 µm
2 µm
2 µm

SI-Fig. S19. XRF and the sum of the intensity of the SAXS patterns of Loc 9,10,11,13 in carious and transition region. XRF analysis of Loc 9,10,11,13 with a step of 150 nm, exposure of 5 s, Ca K_{α} fluorescence intensity map, map of the sum of the intensity of SAXS patterns acquired on the FIB-lamella simultaneously as the XRF, and superimposition of both images. The analysis was done at 12 keV.

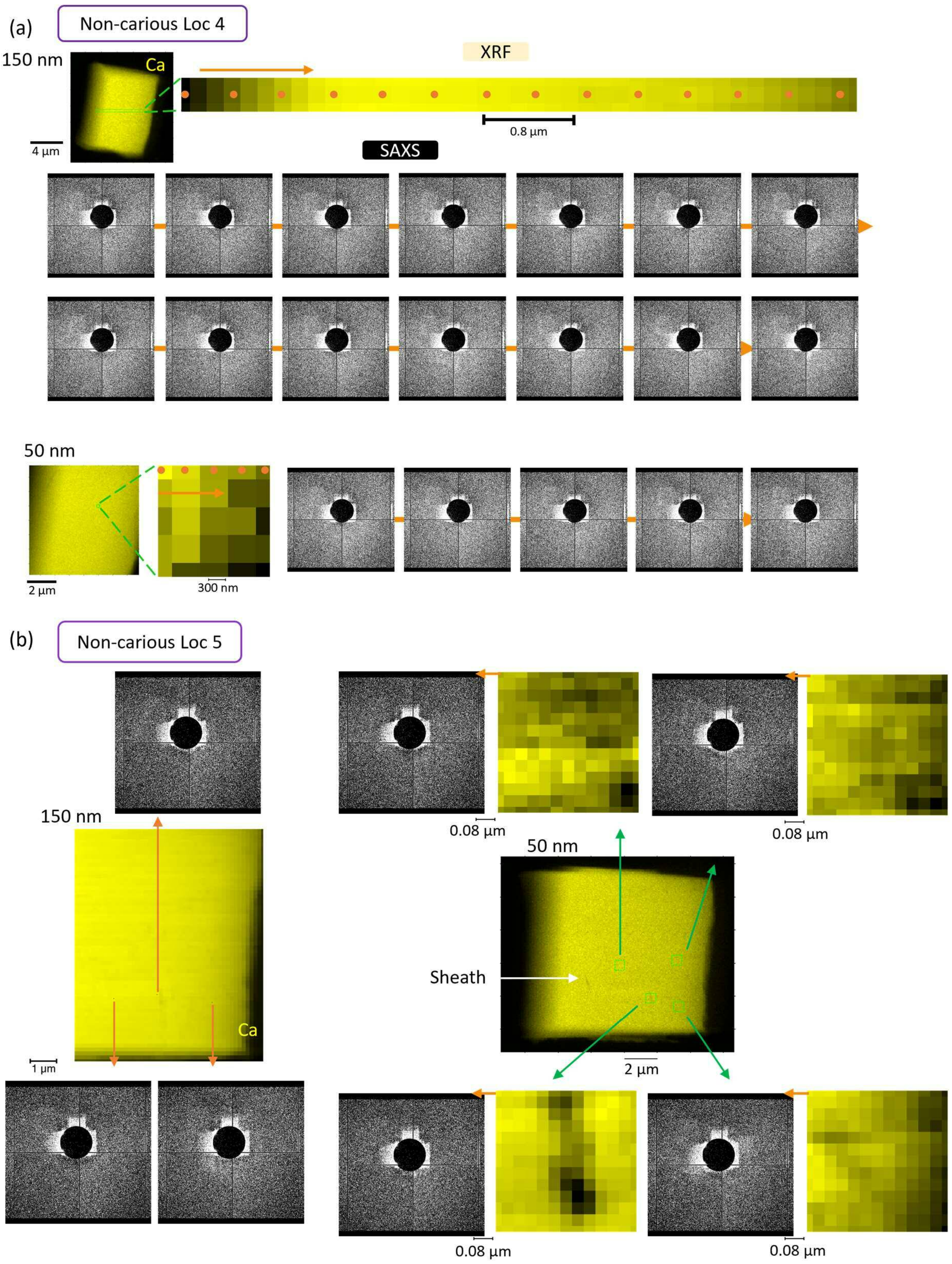

SI-Fig. S20. XRF/SAXS analysis of the non-carious region, Loc 4. (a) XRF/SAXS analysis of Loc 4 in the non-carious region. Ca K_{α} XRF map acquired with a step of 150 nm, exposure of 0.015 ms, region of interest highlighted. The region of interest with the acquisition of XRF and SAXS, map of Ca acquired with a step of 150 nm, exposure 5 s, simultaneously with SAXS (analysis without flight tube at 12 keV). Extraction of SAXS patterns from various pixels showing less scattering than the carious sample, see Figure 4. (b) Similar analysis as (a) for the Loc 5 with an acquisition with a step of 150 nm and 5 s exposure for SAXS and XRF over a large region, and a step of 50 nm (in focus) in the region of

interest respectively (acquisition for 5 s). The analysis was done with a flight tube. Several locations were illustrated to highlight the possibility to extract patterns and confirming the patterns seen.

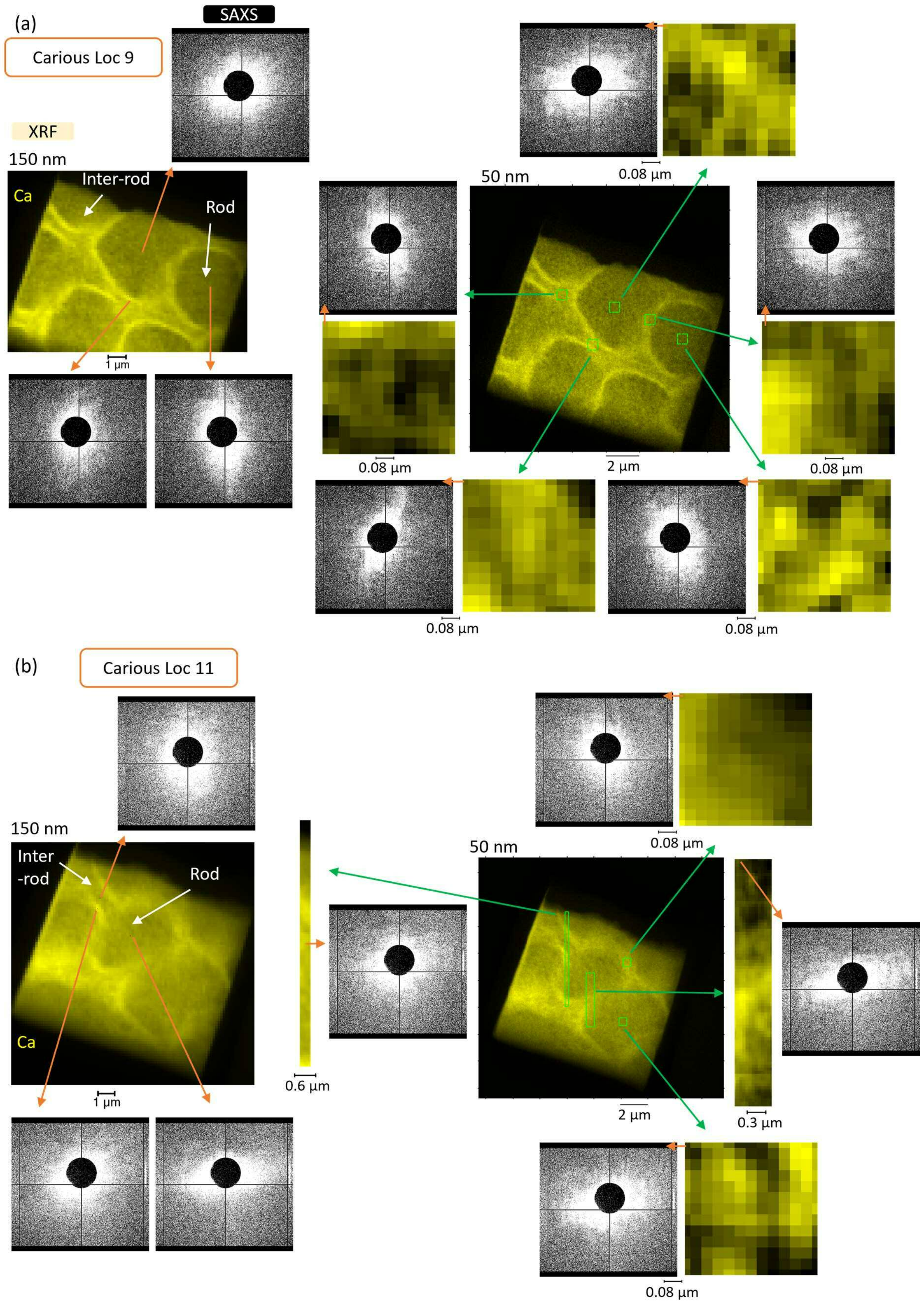

SI-Fig. S21. XRF and SAXS analysis of Loc 9 and Loc 11 in the carious region. (a) Analysis of Loc 9 with XRF map of Ca K$_{\alpha}$ fluorescence intensity acquired with steps of 150 nm and 50 nm. For the low resolution, SAXS were acquired simultaneously and the patterns were extracted from rod and inter-rod regions. For the analysis in focus, several regions of interest were acquired, for each XRF and

SAXS acquired, the XRF map is shown with a SAXS pattern from each region. Clear identification of the scattering directions on the patterns. (b) Similar analysis as (a) but carried out on Loc 11. The analyses were done at 12 keV, for the step of 150 nm (unfocus) acquisition time of 5 s, and for the step of 50 nm (in focus), 0.015 s for the overview, and 5 s of exposure for each region of interest.

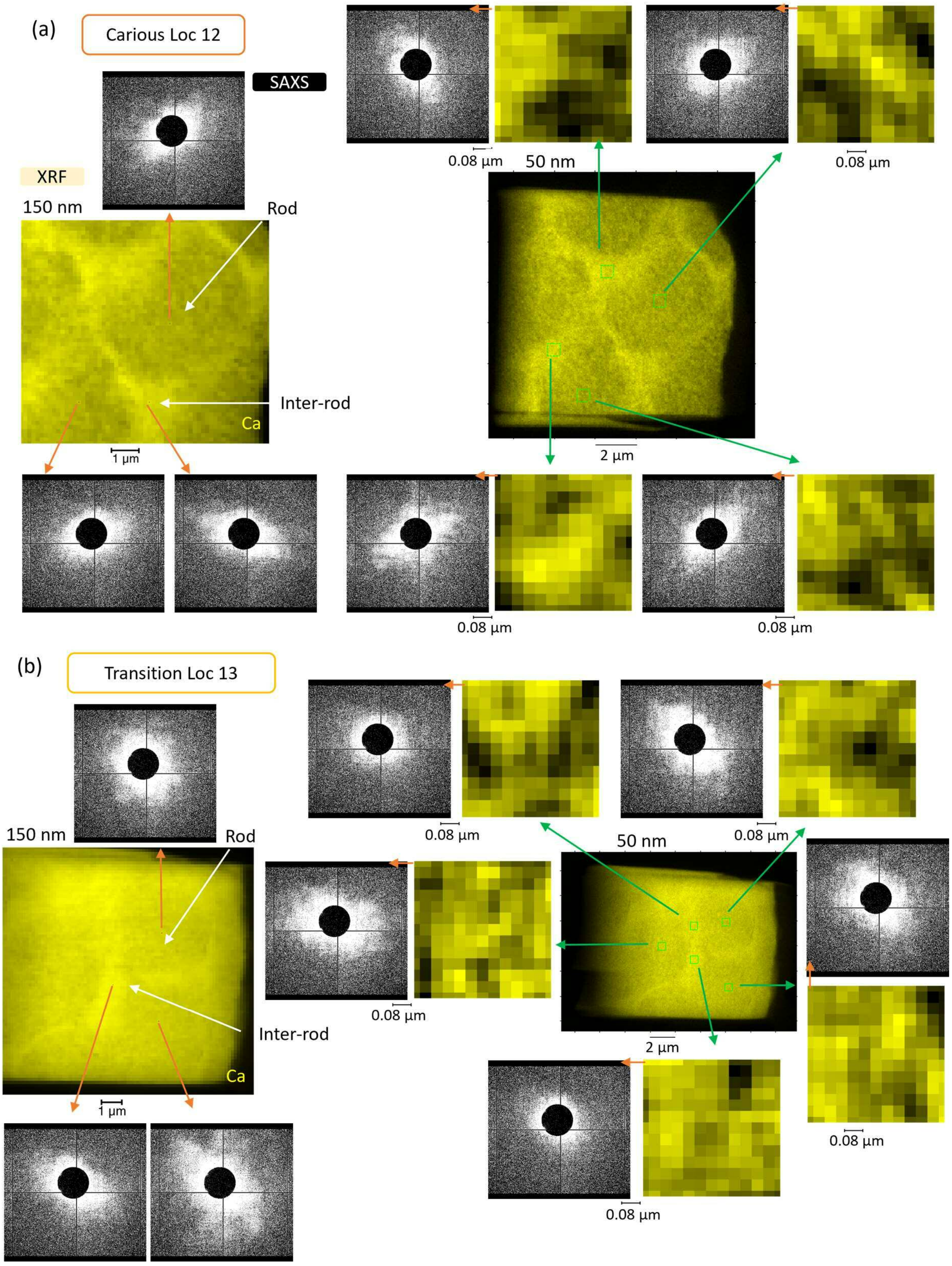

SI-Fig. S22. XRF and SAXS analysis of Loc12 and Loc 13 in the carious and transition region. (a) Analysis of Loc 12 with XRF map of Ca K_α fluorescence intensity acquired with steps of 150 nm and 50 nm. For the low resolution, SAXS were acquired simultaneously and the patterns were extracted from rod and inter-rod regions. For the analysis in focus, several region of interest were acquired, for each XRF and SAXS acquired, a XRF map is shown with a SAXS pattern from each region. Clear identification of the scattering directions on the patterns. (b) Similar analysis as (a) but carried out

on Loc 13 in the transition zone. The analyses were done at 12 keV, for the step of 150 nm (unfocus) acquisition time of 5 s, and for the step of 50 nm (in focus), 0.015 s for the overview, and 5 s of exposure for each region of interest.

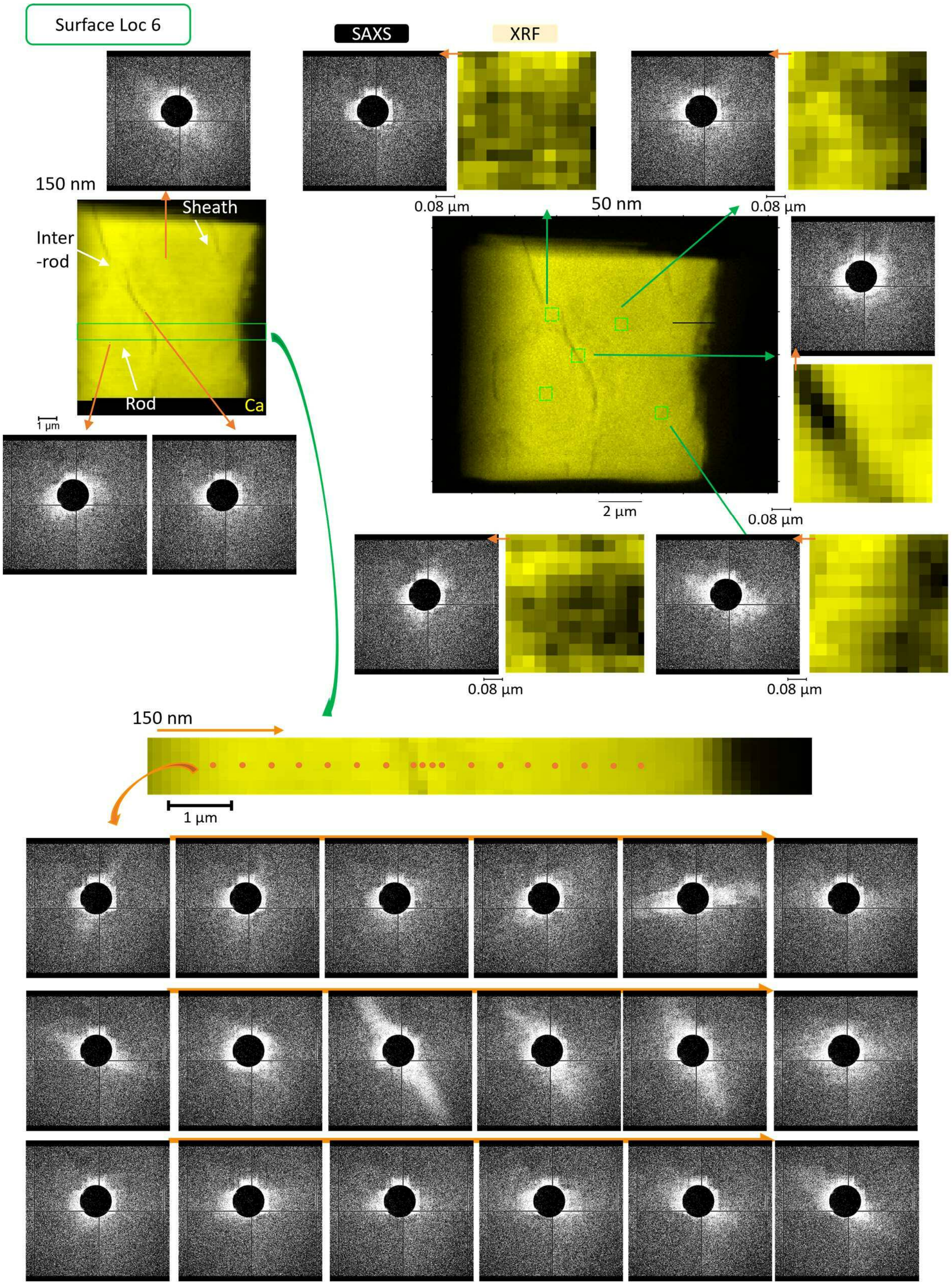

SI-Fig. S23. XRF and SAXS analysis of Loc 6 in the surface region. Analysis of Loc 6 with XRF map of Ca K_{α} fluorescence intensity acquired with steps of 150 nm and 50 nm. For the low resolution, SAXS were acquired simultaneously and the patterns were extracted in the different regions. For the high resolution, several regions of interest were acquired, for each XRF and SAXS acquired, a XRF map is

shown with a SAXS pattern from each region. Clear identification of the scattering directions on the patterns.

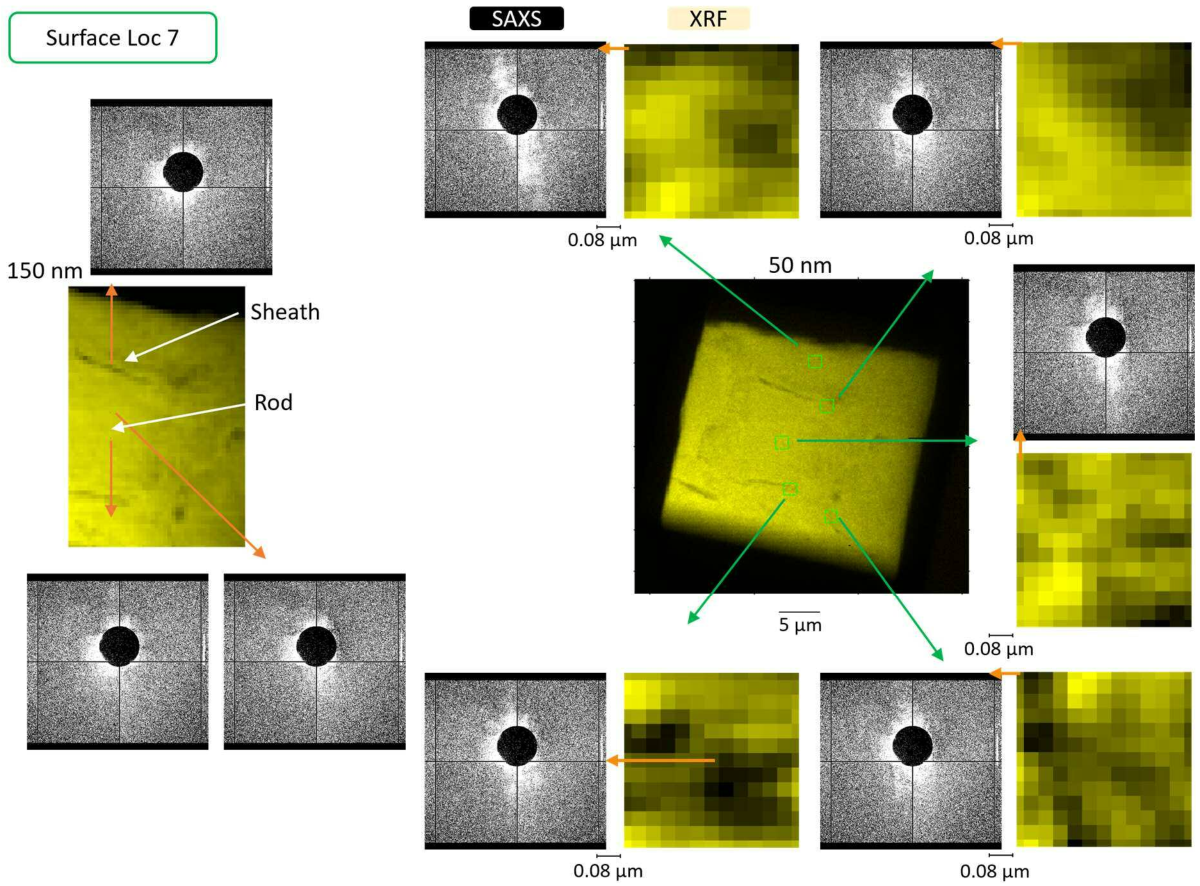

SI-Fig. S24. XRF and SAXS analysis of Loc 7 in the surface region. Analysis of Loc 7 with XRF map of Ca K_α fluorescence intensity acquired with steps of 150 nm and 50 nm. For the step of 150 nm (unfocus), SAXS were acquired simultaneously and the patterns were extracted from rod and inter-rod regions. For the high resolution, several regions of interest were acquired, for each XRF and SAXS acquired, a XRF map is shown with a SAXS pattern from each region. Clear identification of the scattering on the patterns. The analysis were done at 12 keV, for the low resolution acquisition time of 5 s, and for the step of 50 nm (in focus), 0.015 s for the overview, and 5 s exposure for each region of interest.

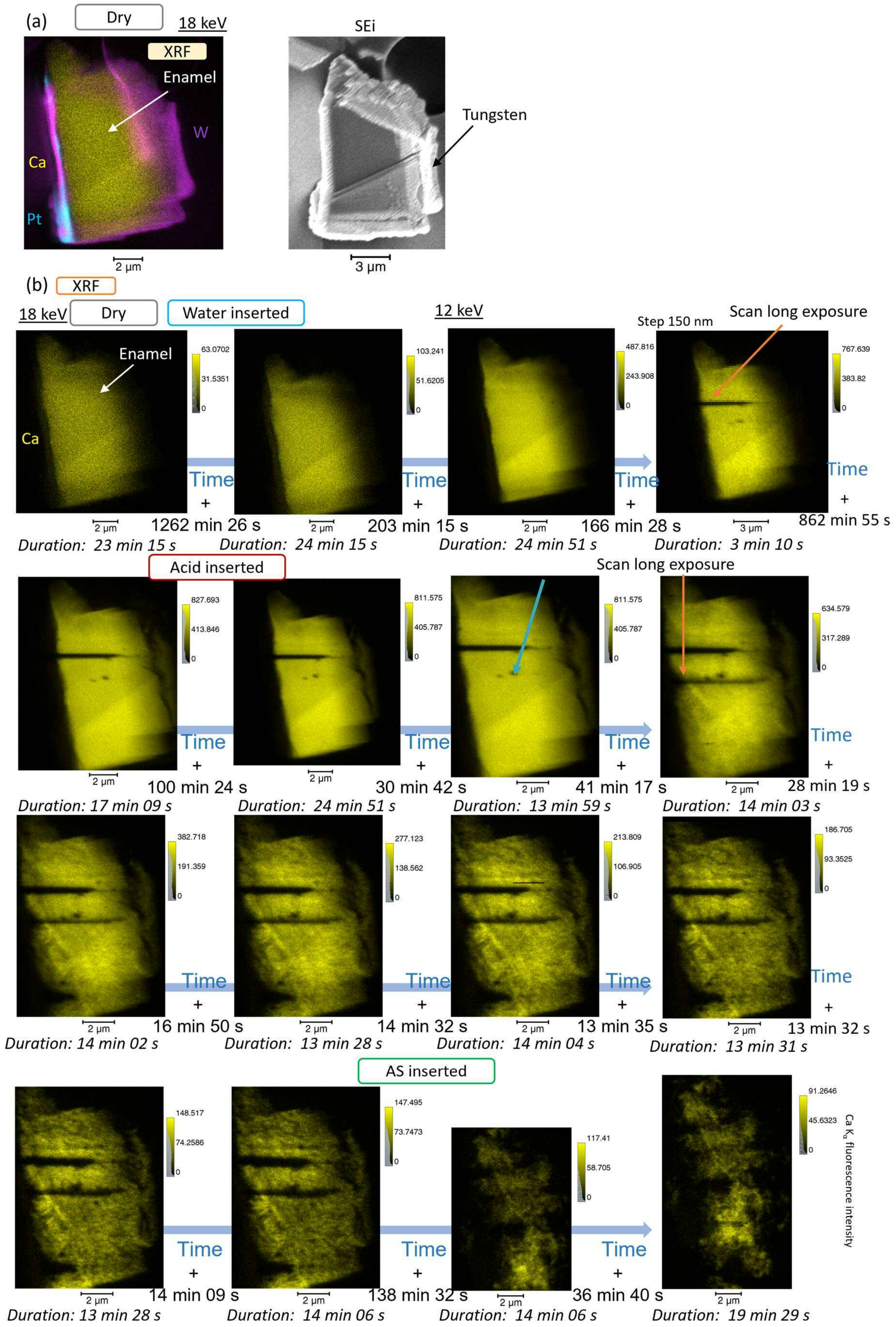
(a)
Dry
18 keV
XRF
Enamel
W
Ca
Pt
2 µm
SEi
Tungsten
3 µm
(b)
XRF
18 keV
Dry
Water inserted
12 keV
Step 150 nm
Scan long exposure
Enamel
Ca
Time
+
1262 min 26 s
Duration: 23 min 15 s
203 min 15 s
Duration: 24 min 15 s
166 min 28 s
Duration: 24 min 51 s
862 min 55 s
Duration: 3 min 10 s
Acid inserted
Scan long exposure
100 min 24 s
Duration: 17 min 09 s
30 min 42 s
Duration: 24 min 51 s
41 min 17 s
Duration: 13 min 59 s
28 min 19 s
Duration: 14 min 03 s
16 min 50 s
Duration: 14 min 02 s
14 min 32 s
Duration: 13 min 28 s
13 min 35 s
Duration: 14 min 04 s
13 min 32 s
Duration: 13 min 31 s
AS inserted
14 min 09 s
Duration: 13 min 28 s
138 min 32 s
Duration: 14 min 06 s
36 min 40 s
Duration: 14 min 06 s
Duration: 19 min 29 s
Ca Kα fluorescence intensity

SI-Fig. S25. XRF spectroscopy analysis of the FIB-lamella Loc 14 during *in situ* experiment. (a) Initial condition in dry condition with the location of the FIB-lamella and the XRF intensity of Ca, Pt and tungsten (W), step 50 nm, exposure 0.015 ms and energy 18 keV and SEi of the FIB-lamella positioned on the chip. (b) Ca intensity from the dry condition, during the flow of water (5 $\mu L.min^{-1}$), and then during the flow of acid and artificial saliva (AS). During the acid, illustration of several maps with the acquisition of 0.015 ms and step 50 nm which were carried out (one map was acquired with a step of 150 nm, and unfocus). The duration of each map is reported as well as time between maps (ending one map to the ending time of the other map). There is the highlight of the damages found during the time study from the localised region of interest map, acquisition 10 s. Additional details in SI-Fig. S26 with plot of intensity vs time and SI-Fig. S27 with the position of the region of interest.

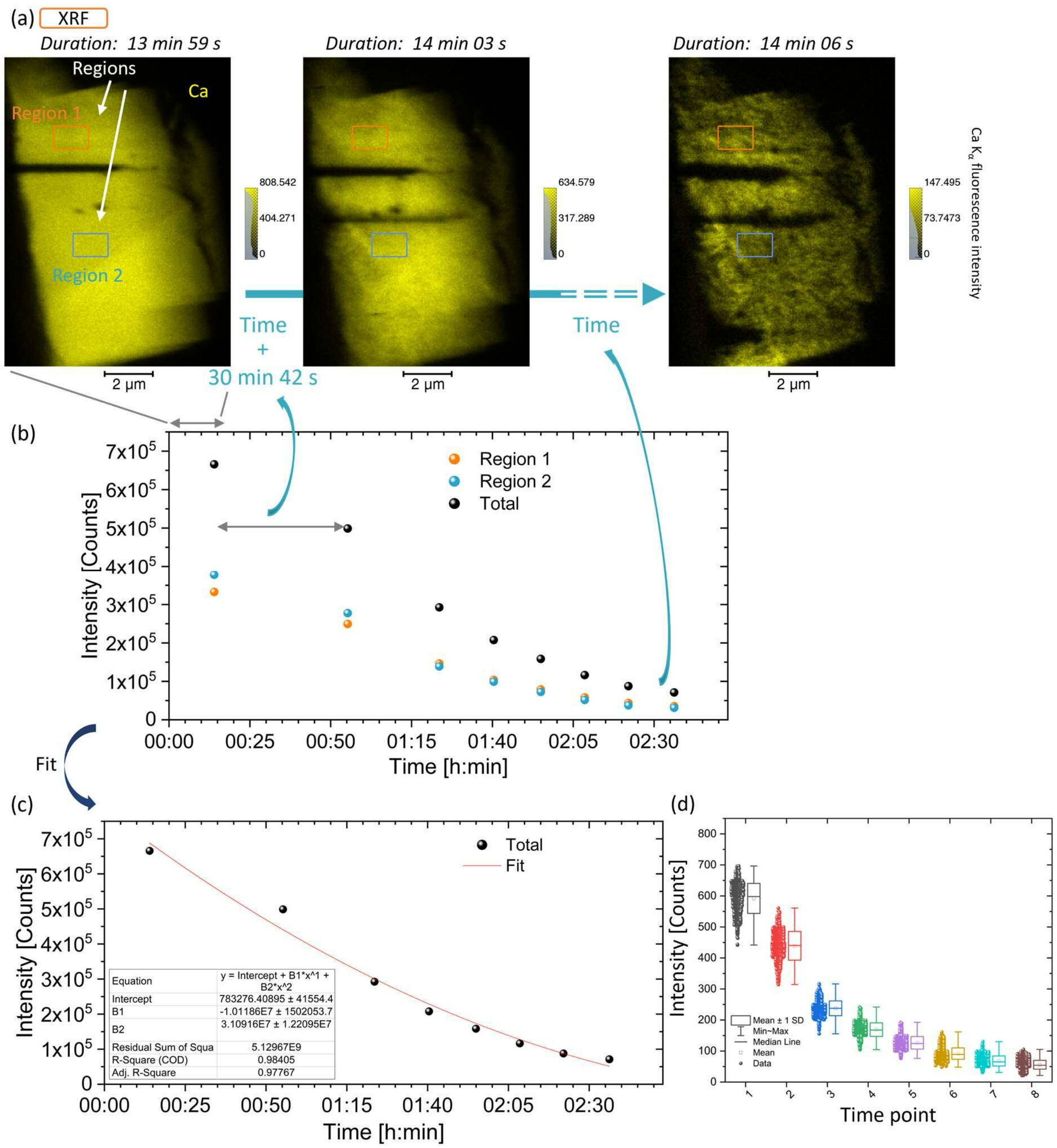

SI-Fig. S26. XRF spectroscopy analysis of the FIB-lamella Loc 14 during *in situ* experiment with the evolution of Ca intensity with time. (a) Ca fluorescence intensity maps acquired during the flow of acid (see SI-Fig. S25 for more details, these maps are also shown in SI-Fig. S25), with the illustration of several maps with the acquisition of 0.015 ms and step 50 nm, and the same dimension (9.4 × 12.8 µm, pixel size of 50 nm). The duration of the acquisition of individual map is shown, and then cumulative time is analysed. The time was determined from the starting time of the first map shown in the Figure to the ending time of the last map illustrated in the Figure. On the map, two regions were analysed, referred to as Region 1 and 2 (1.45 × 0.95 µm each from the full maps of dimension 9.4 × 12.8 µm, pixel size 50 nm). (b) The sum of the fluorescence intensity of Ca for the two regions, summed and plotted as a function of the time (the starting point at 0 min is from the starting of the acquisition of the first map shown in the Figure). This revealed a non-linear decrease of the Ca intensity with time (dissolution rate non-linear as reported from tomography data for instance[2]) as well as variations between regions. These regions were chosen to limit the contribution from the

large damages seen on the maps. (c) Plot of the fit of the sum of Ca fluorescence intensity of the two regions (referred to as 'total') showing the non-linear decay of intensity as a function of time (polynomial fit used). The analyses were done with DAWN, Avizo, Matlab and OriginPro. (d) Statistical analysis of the dataset described in (b) and (c) from each pixel of the two regions. One-way ANOVA test with post hoc Tukey's test was carried out. There are significant differences ($p \leq 0.0001$) between each time point..

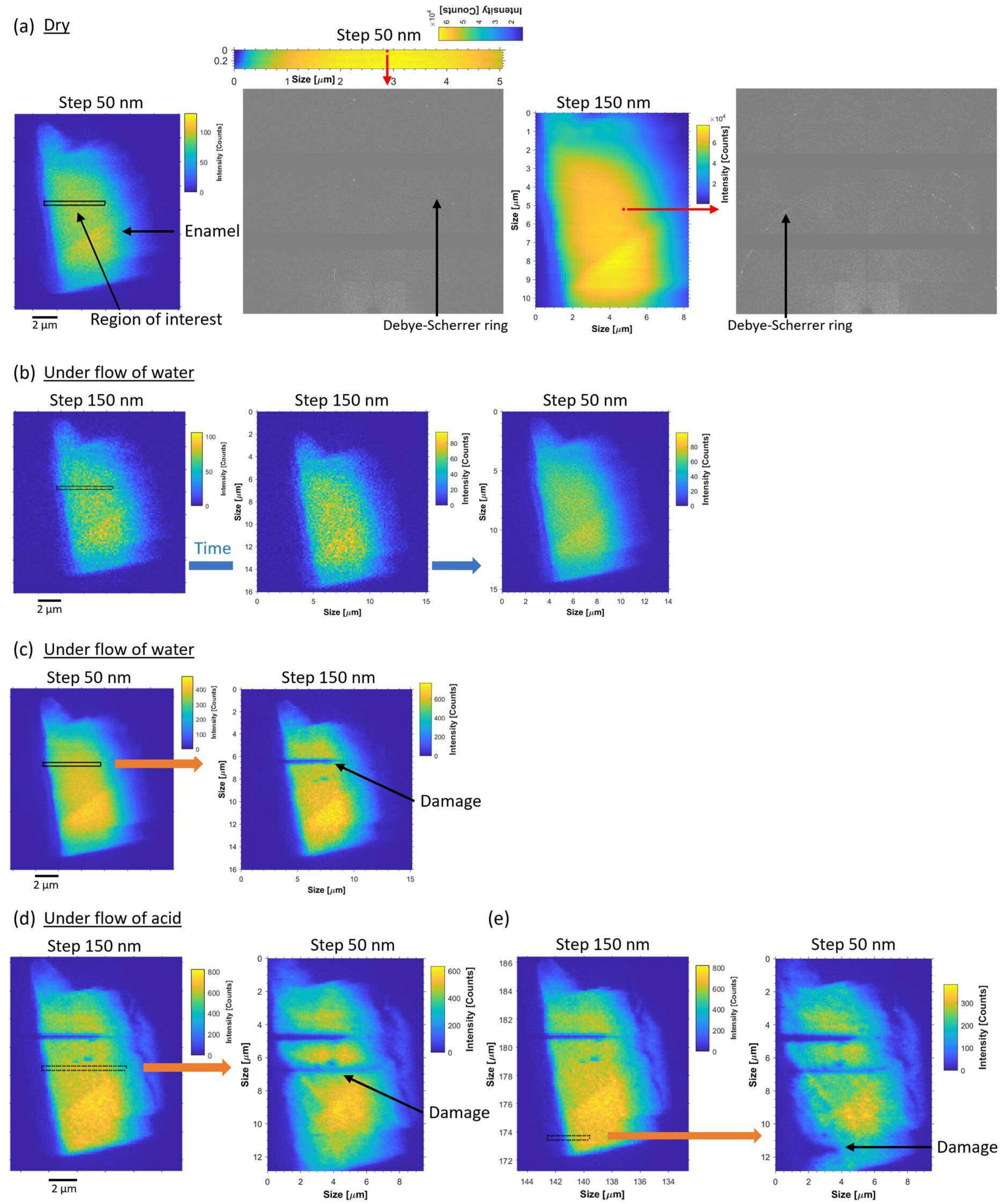

SI-Fig. S27. XRF analysis during the *in situ* experiment with the position of large scans and region of interest analysis. Additional details for the SI-Fig. S25. a) Details of the Ca map with a step of 50 nm, exposure of 0.015 s and region of interest (5 × 0.3 µm) with a step of 50 nm, exposure of 5 s and Ca map with a step of 150 nm, exposure of 10 s for the WAXS pattern extracted. (b) Details of the Ca map with a step of 150 nm, exposure of 0.015 s and region of interest (5 × 0.3 µm) with a step of 150 nm, exposure of 10 s and the XRF map after the acquisition with a step of 150 nm and 50 nm, the flow of water, energy 18 keV. The region of interest was repeated a few times before the new acquisition with the step of 50 nm. (c) Details of the Ca map with a step of 50 nm, exposure 0.015 s and region of interest analysed (5 × 0.3 µm) with a step of 50 nm, exposure of 10 s, and the Ca map after the acquisition of the region of interest, a step of 150 nm, exposure of 0.015 s, the flow of

water, energy 12 keV. (d) Details of the Ca map at a step of 150 nm, with a region of interest analysed (6 × 0.3 µm) with a step of 150 nm, exposure of 10 s, and the Ca map with a step of 50 nm (exposure 0.015 s) after the scan of the ROI, the flow of acid, energy 12 kV. (e) Details of the Ca map at step of 150 nm, exposure of 0.015 s, with another region of interest analysed (3 × 0.6 µm) with a step of 150 nm, exposure of 10 s, and the Ca map with a step of 50 nm (exposure of 0.015 s) after the scan of the ROI.

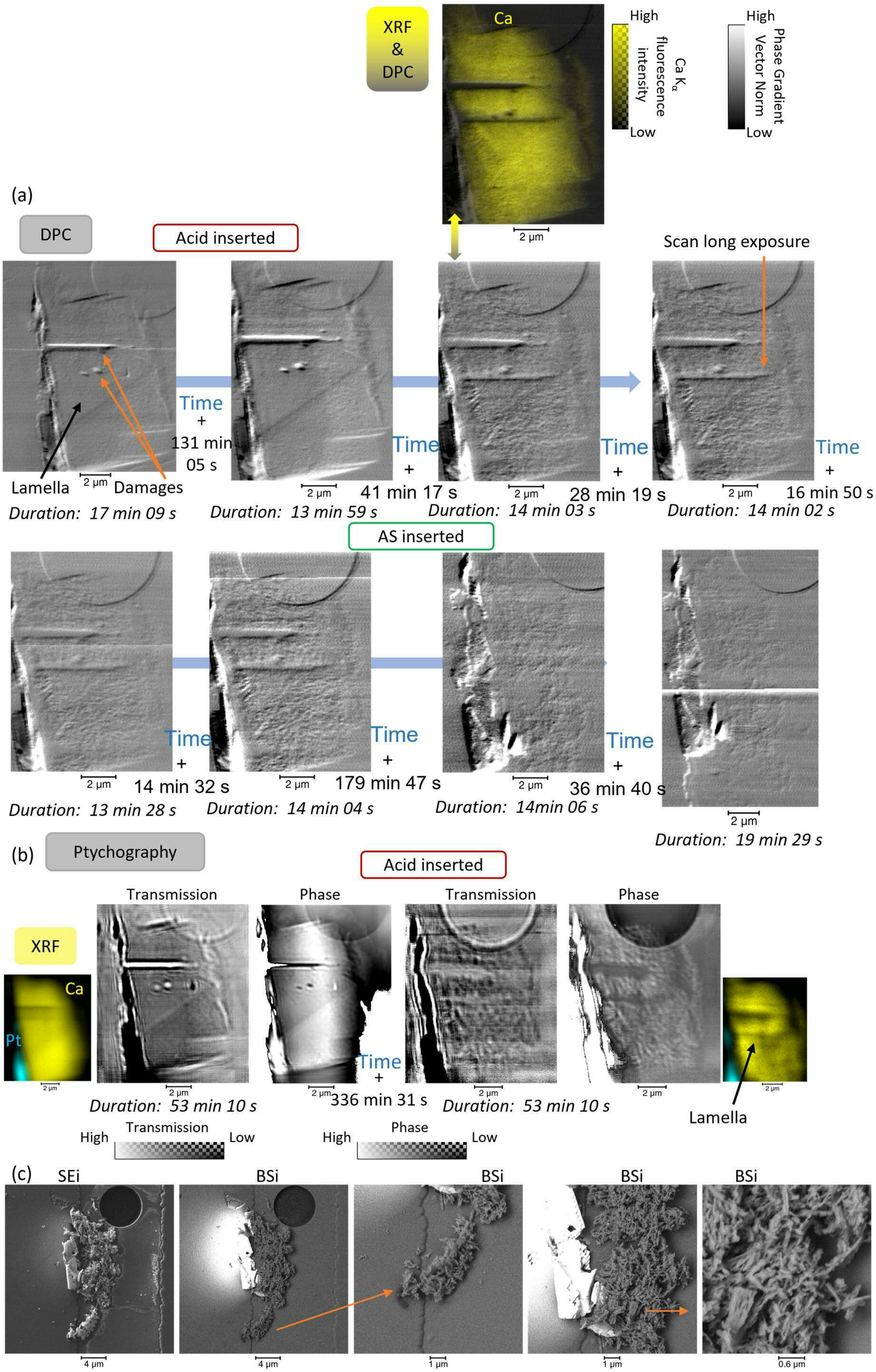
XRF & DPC
Ca
High
Ca K_α fluorescence intensity
Low
High
Phase Gradient Vector Norm
Low
(a)
DPC
Acid inserted
Scan long exposure
Lamella
Damages
Time + 131 min 05 s
Time + 41 min 17 s
Time + 28 min 19 s
Time + 16 min 50 s
Duration: 17 min 09 s
Duration: 13 min 59 s
Duration: 14 min 03 s
Duration: 14 min 02 s
AS inserted
Time + 14 min 32 s
Time + 179 min 47 s
Time + 36 min 40 s
Duration: 13 min 28 s
Duration: 14 min 04 s
Duration: 14min 06 s
Duration: 19 min 29 s
(b)
Ptychography
Acid inserted
Transmission
Phase
Transmission
Phase
XRF
Ca
Pt
Time + 336 min 31 s
Duration: 53 min 10 s
Duration: 53 min 10 s
Lamella
High Transmission Low
High Phase Low
(c)
SEi
BSi
BSi
BSi
BSi
2 µm
4 µm
1 µm
0.6 µm

SI-Fig. S28. DPC and ptychography analysis with structural details of the FIB-lamella Loc 14 during the *in-situ* experiment in comparison to XRF. (a) DPC images of the FIB-lamella during the flow of water, then the flow of acid, and finally, artificial saliva, for the scans acquisition of 0.015 s and a step of 50 nm were used. For one time point, superimposition of the XRF map of Ca and DPC image (XRF maps detailed in SI-Fig. S25). The duration of each DPC map is reported as well as time between maps (ending one map to the ending time of the other map, the same for the (b) for the ptychography data). (b) Transmission and phase image from the reconstructed ptychography data (reconstructed with a pixel size of 31.921 nm, 12 keV) of the FIB-lamella during exposure to water, and during the flow of acid, acquisition 0.25 s and step of 100 nm. (c) SEM images with SEi and BSi of the sample after the experiment with zoom-in images showing the remaining crystallites.

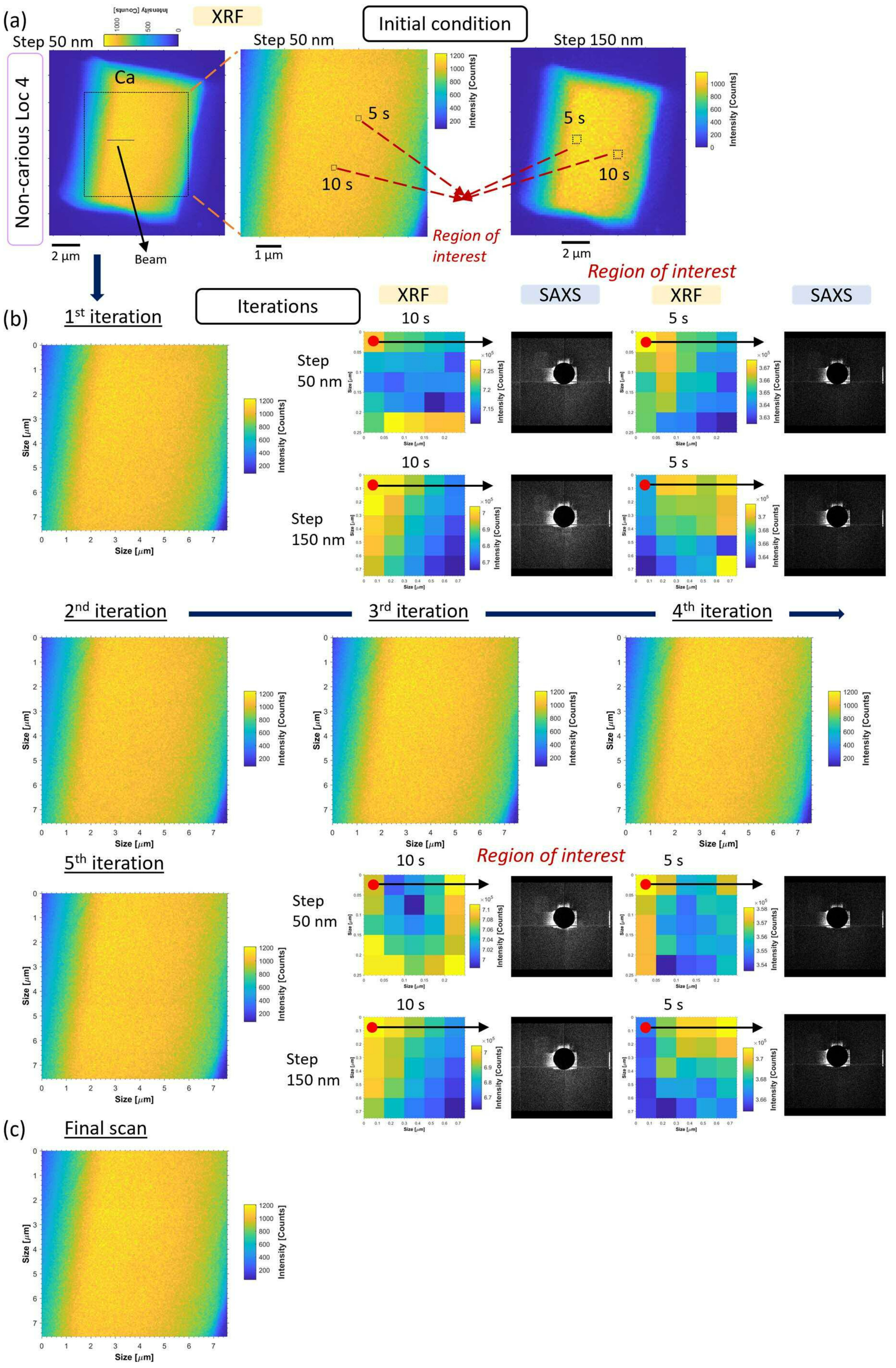
(a)
XRF
Initial condition
Step 50 nm
Step 50 nm
Step 150 nm
Non-carious Loc 4
Ca
5 s
10 s
5 s
10 s
Region of interest
2 µm
Beam
1 µm
2 µm
Region of interest
(b)
1st iteration
Iterations
XRF
SAXS
XRF
SAXS
10 s
5 s
Step 50 nm
Step 150 nm
2nd iteration
3rd iteration
4th iteration
5th iteration
Region of interest
(c)
Final scan
Size [µm]
Intensity [Counts]

SI-Fig. S29. Study of repeated exposure to the X-ray on regions of interest in the Loc 4, SAXS and XRF acquired at high and low resolution. (a) XRF map of Ca from the two resolutions 50 and 150 nm (in and out of focus respectively, exposure 0.015 s for the large map) with the details of the regions where the scan was repeated with 5 or 10 s in each resolution. (b) XRF map of Ca with a step of 50 nm, exposure 0.015 s at different iterations, in each iteration both low and high-resolution region of interest was acquired (5 and 10 s). For the iteration 1 and 5, XRF maps of the region of interest from the two resolutions performed and time acquired with for each the SAXS diffraction pattern extracted from one pixel. (c) XRF map of Ca with a step of 50 nm, exposure of 0.015 s, after the iterations. The exposure is per point.

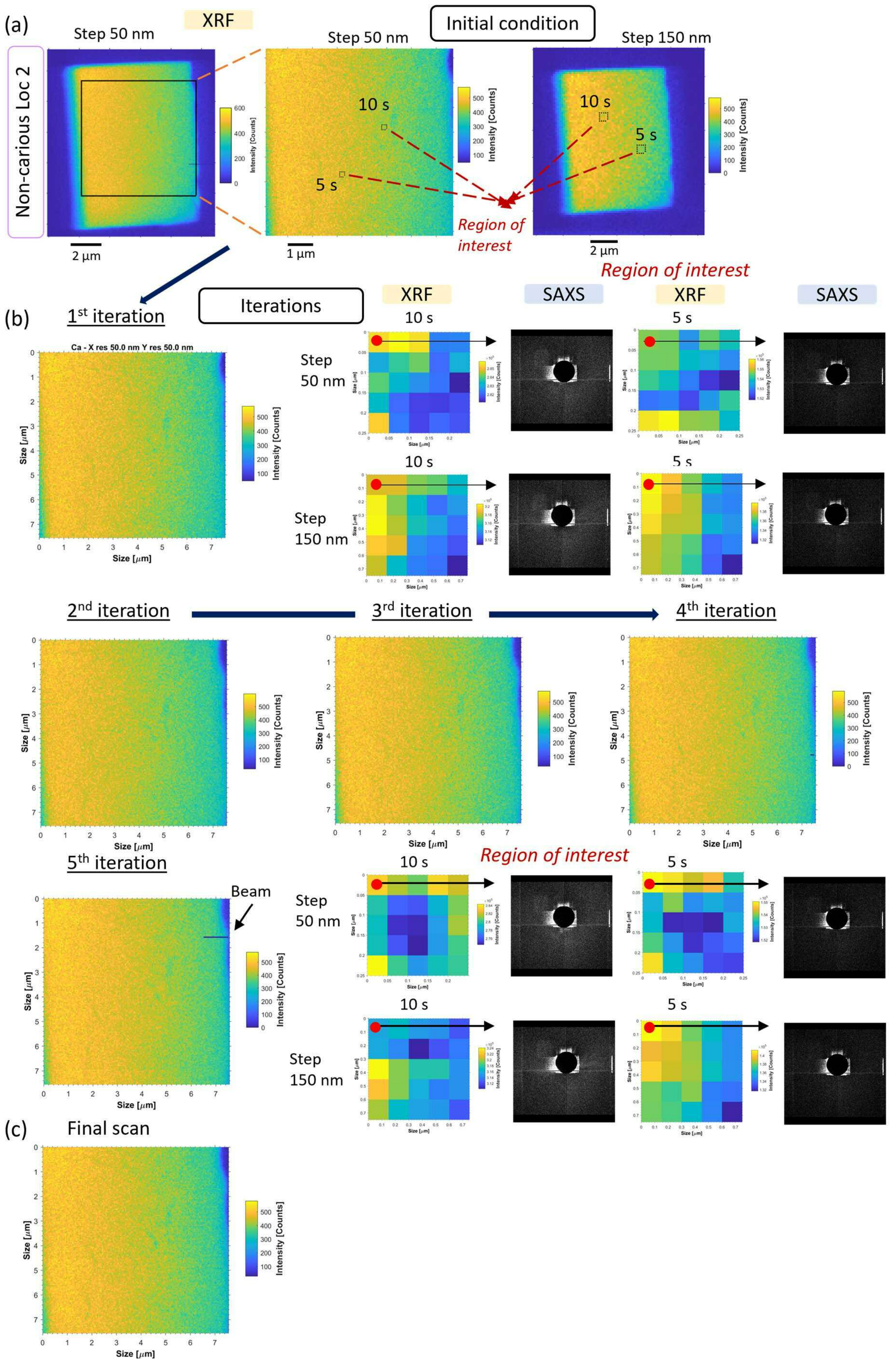
(a)
Non-carious Loc 2
Step 50 nm
XRF
Initial condition
Step 50 nm
Step 150 nm
10 s
5 s
Region of interest
2 µm
1 µm
(b)
1st iteration
Iterations
Region of interest
XRF
SAXS
10 s
5 s
Step 50 nm
Step 150 nm
Ca - X res 50.0 nm Y res 50.0 nm
Size [µm]
Intensity [Counts]
2nd iteration
3rd iteration
4th iteration
5th iteration
Beam
(c)
Final scan

SI-Fig. S30. Study of repeated exposure to the X-ray on regions of interest in the Loc 2, SAXS and XRF acquired at high and low resolution. (a) XRF map of Ca from the two resolutions 50 and 150 nm (in and out of focus respectively, exposure 0.015 s for the large map) with the details of the regions where the scan was repeated with 5 or 10 s in each resolution. (b) XRF map of Ca with a step of 50 nm, exposure 0.015 s at different iterations, in each iteration both low and high-resolution region of interest was acquired (5 and 10 s). For the iteration 1 and 5, XRF maps of the region of interest from the two resolutions performed and time acquired with for each the SAXS diffraction pattern extracted from one pixel. (c) XRF map of Ca with a step of 50 nm, exposure of 0.015 s, after the iterations. The exposure is per point. The analyses in this Figure are in agreement with the other location analysed (SI-Fig. S29).

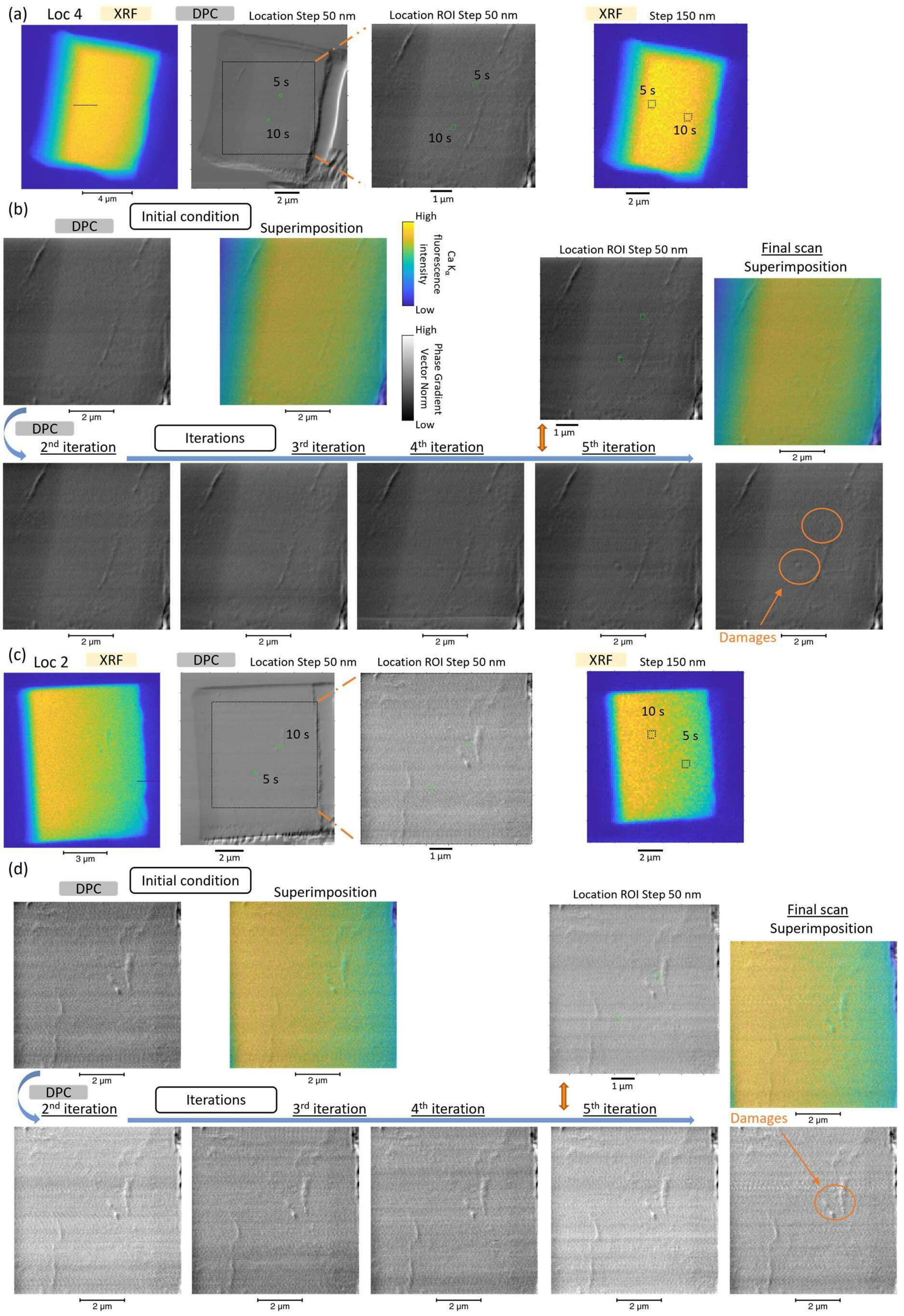

SI-Fig. S31. DPC and XRF analysis of the Loc 4 and Loc 2 during exposure iterations. (a) DPC images of the FIB-lamella Loc 4 with the locations of the region of interest used for the multitude scans (step 50 and 150 nm), and XRF maps were also shown. (b) DPC images during the several iterations and

the final scan. Superimposition of XRF and DPC image for the initial condition and final scan acquired on Loc 4. The damages due to the scans are highlighted. (c) DPC images of the FIB-lamella Loc 2 with the locations of the region of interest used for the multitude scans (step 50 and 150 nm), and XRF maps were also shown. (d) DPC images during the several iterations and the final scan acquired on Loc 2. Superimposition of XRF and DPC image for the initial condition and final scan. The damages due to the scans are highlighted. More studies are required to investigate the nature of the damage, structural (when liquid in the *in situ* analysis, dissolution of materials...). Analysis is done at 12 keV.

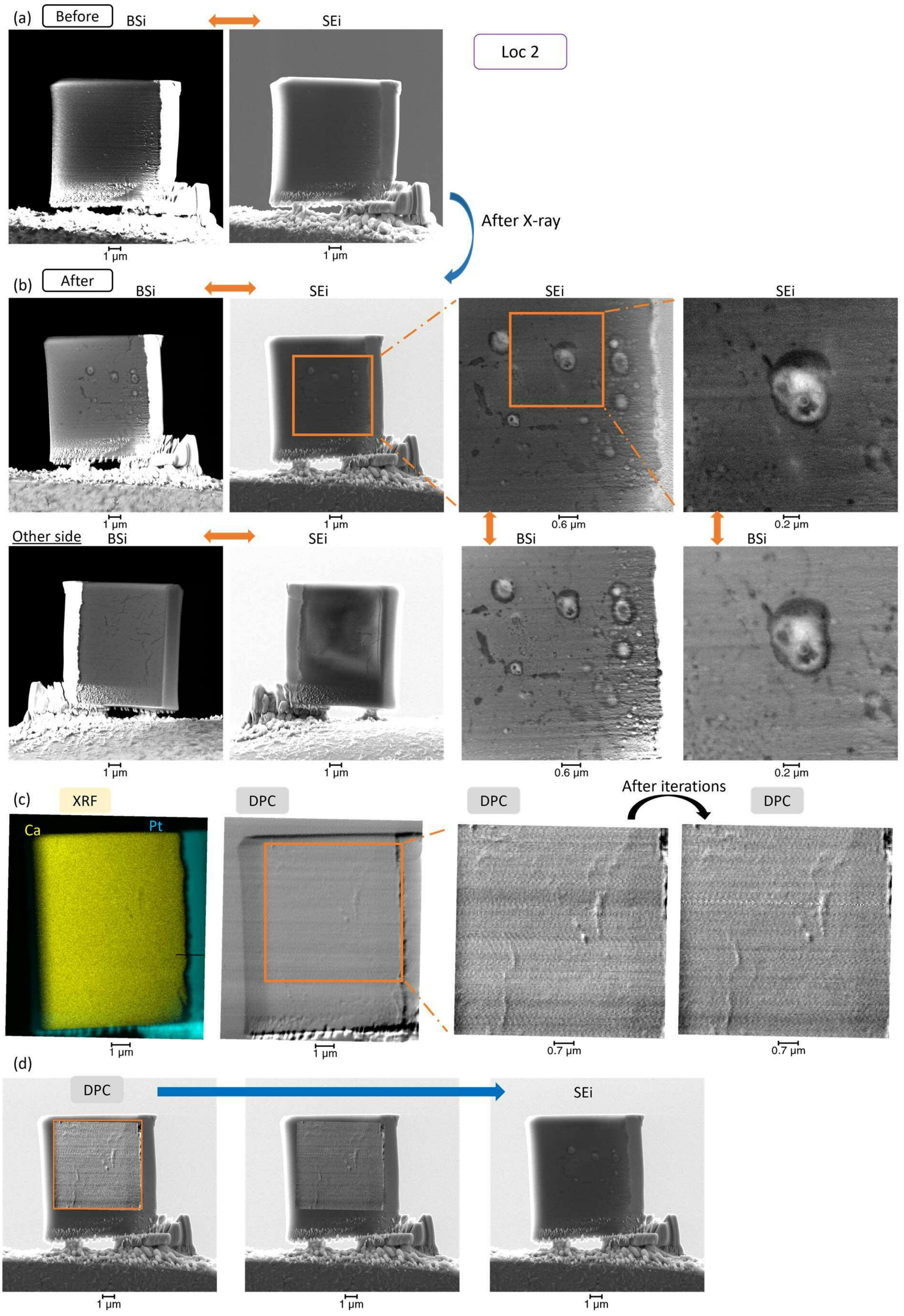

(a)
Before
BSi
SEi
Loc 2
1 µm
1 µm
After X-ray
(b)
After
BSi
SEi
SEi
SEi
1 µm
1 µm
0.6 µm
0.2 µm
Other side
BSi
SEi
BSi
BSi
1 µm
1 µm
0.6 µm
0.2 µm
(c)
XRF
Ca
Pt
DPC
DPC
After iterations
DPC
1 µm
1 µm
0.7 µm
0.7 µm
(d)
DPC
SEi
1 µm
1 µm
1 µm

SI-Fig. S32. SEM and nanoprobe analysis of Loc 2 before and after beam exposure. (a) SEi and BSi of the FIB-lamella of Loc 2 before the synchrotron experiments. (b) SEi and BSi after the experiments and highlight of the modification of the structure. (c) XRF and DPC map with the highlight of the region of interest before and after the iterations described in SI-Figs. S30-31. (d) Image showing the superimposition of the DPC and SEi. Additional damages on the SEM could come from previous X-ray scans.

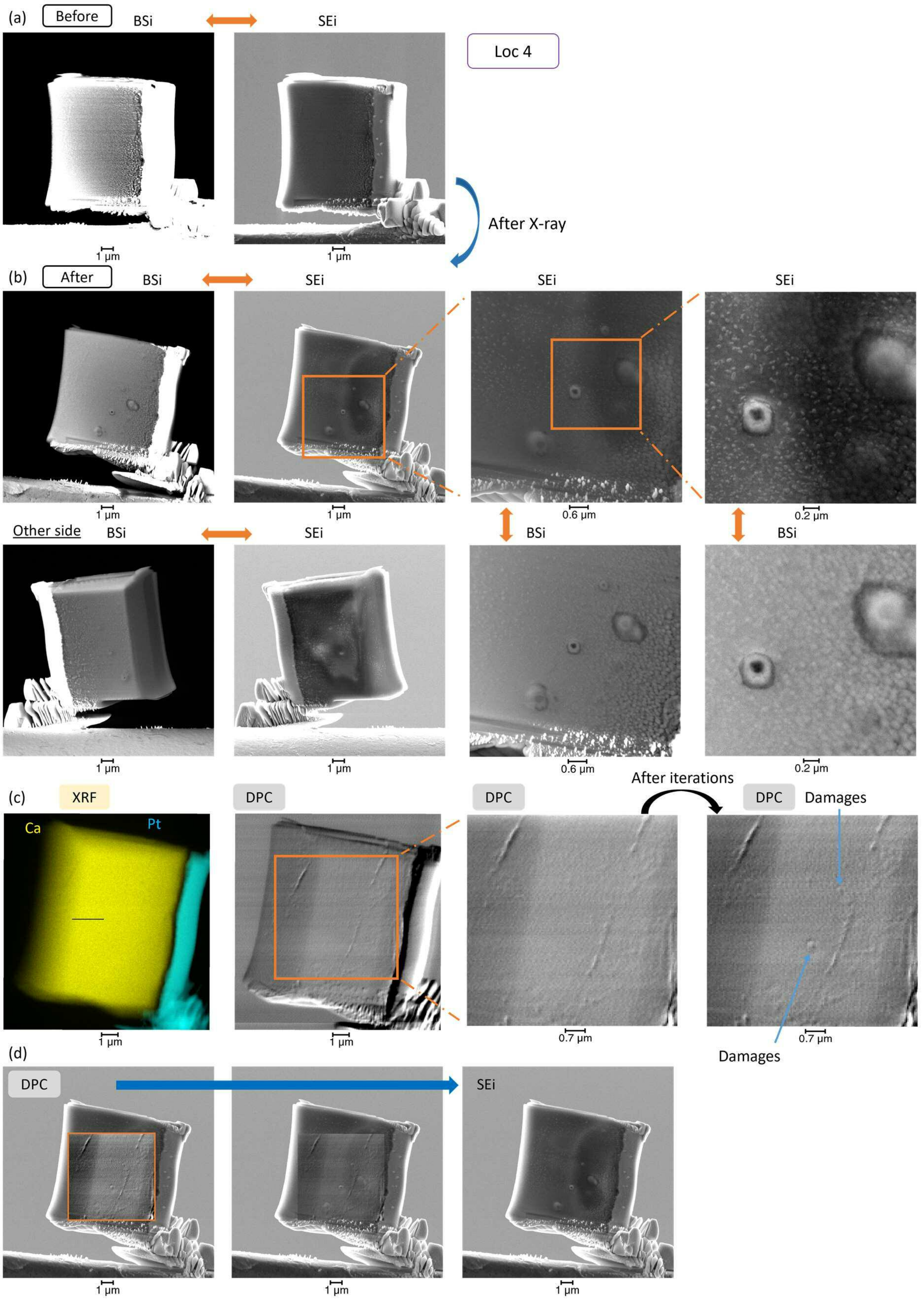

SI-Fig. S33. SEM and nanoprobe analysis of Loc 4 before and after beam exposure. (a) SEi and BSi of the FIB-lamella of Loc 4 before the synchrotron experiments. (b) SEi and BSi after the experiments

and highlight of the modification of the structure. (c) XRF and DPC map with the highlight of the region of interest before and after the iterations described in SI-Figs. S29,31. (d) Image showing the superimposition of the DPC and SEi. Additional damages on the SEM could come from previous X-ray scans.

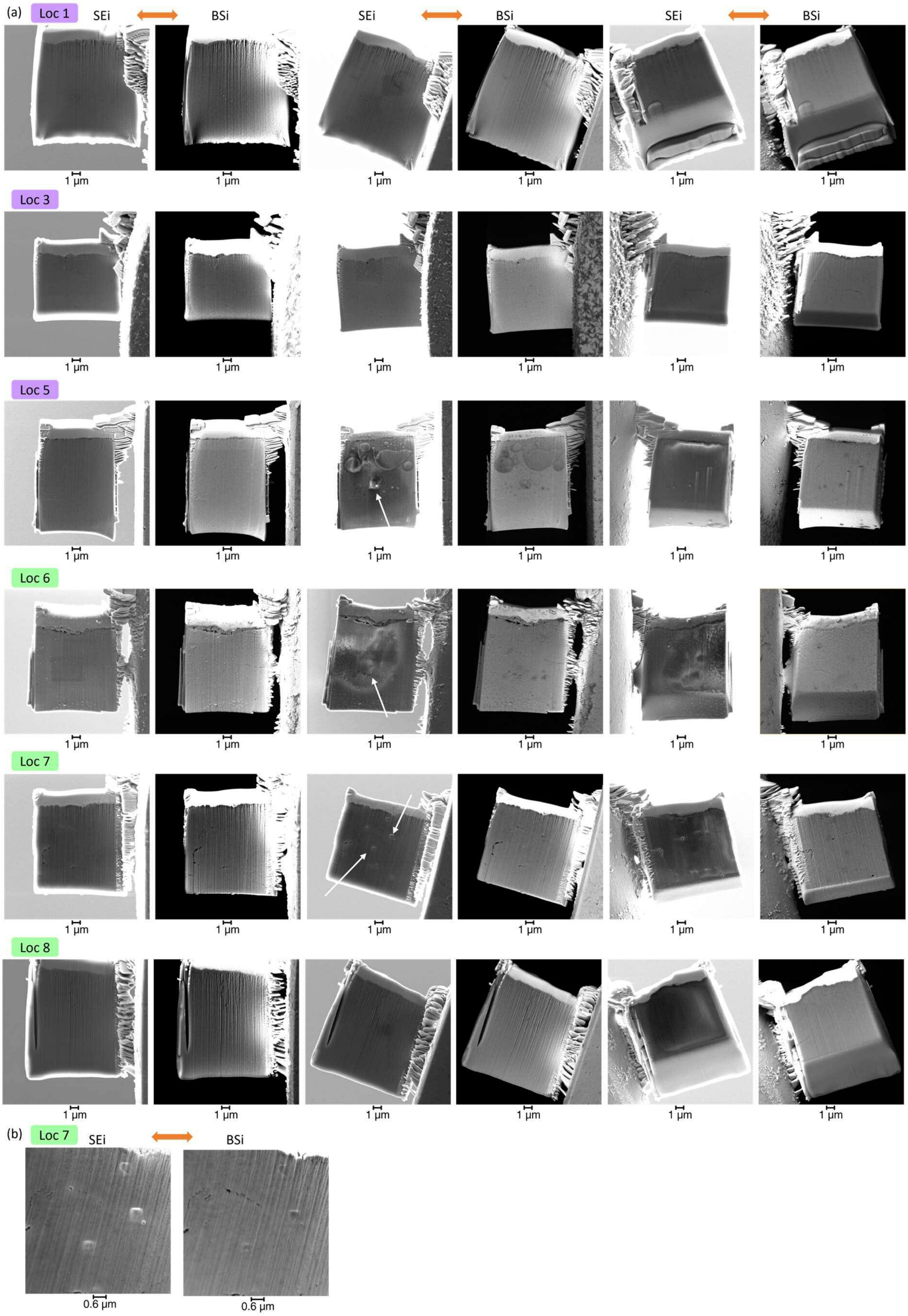

SI-Fig. S34. SEM analysis of locations in non-carious and surface regions before and after synchrotron experiments. (a) SEi and BSi analysis of locations in non-carious and surface regions before and after

synchrotron experiments, arrows highlighted some changes. (b) Zoom in the Loc 7 showing modifications of the structure, linked to the analysis done, SI-Fig. S24.

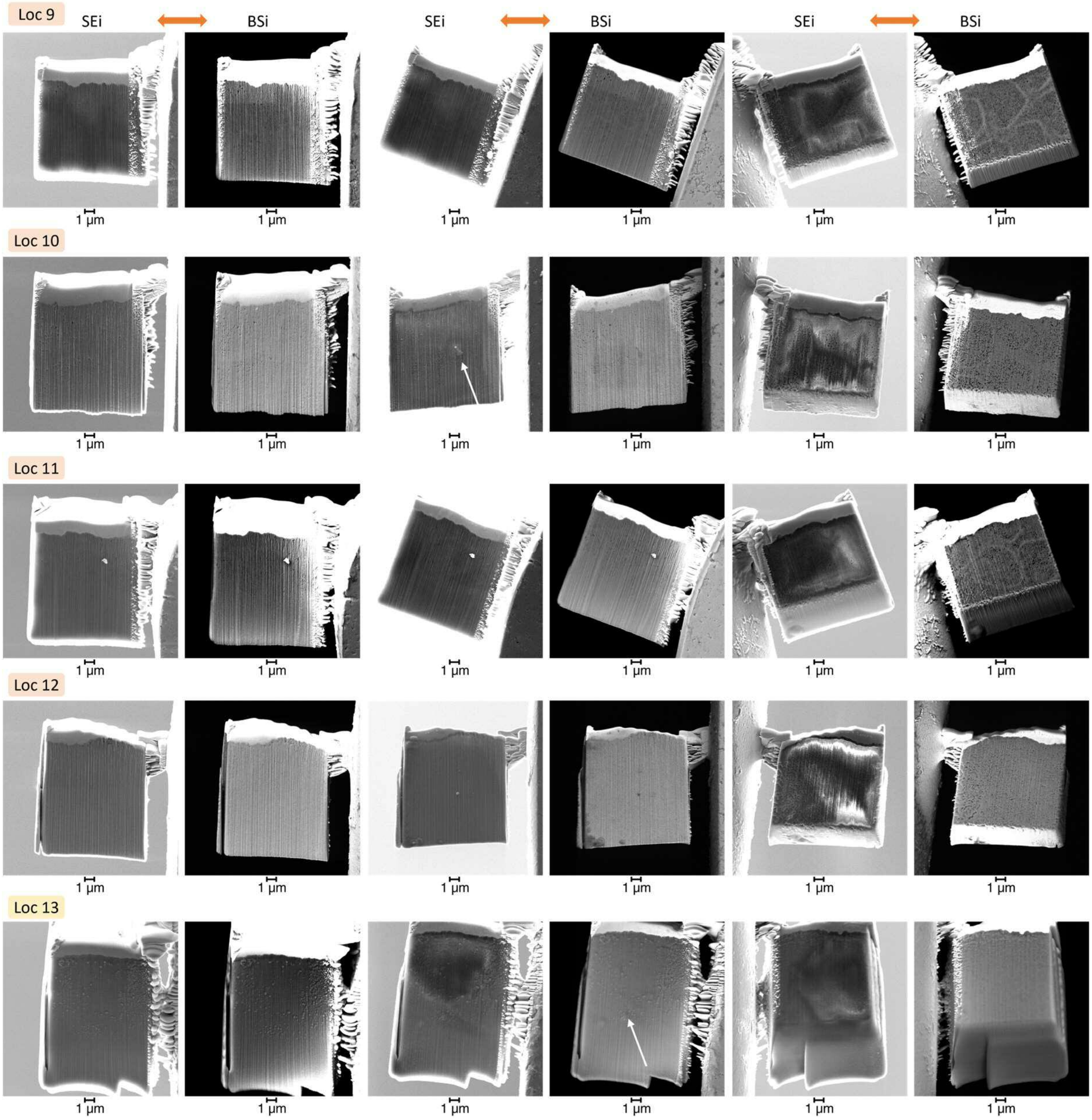

SI-Fig. S35. SEM analysis of locations in the carious and transition regions before and after synchrotron experiments, arrows highlighted some changes. SEi and BSi analysis of locations in non-carious and surface regions before and after synchrotron experiments.

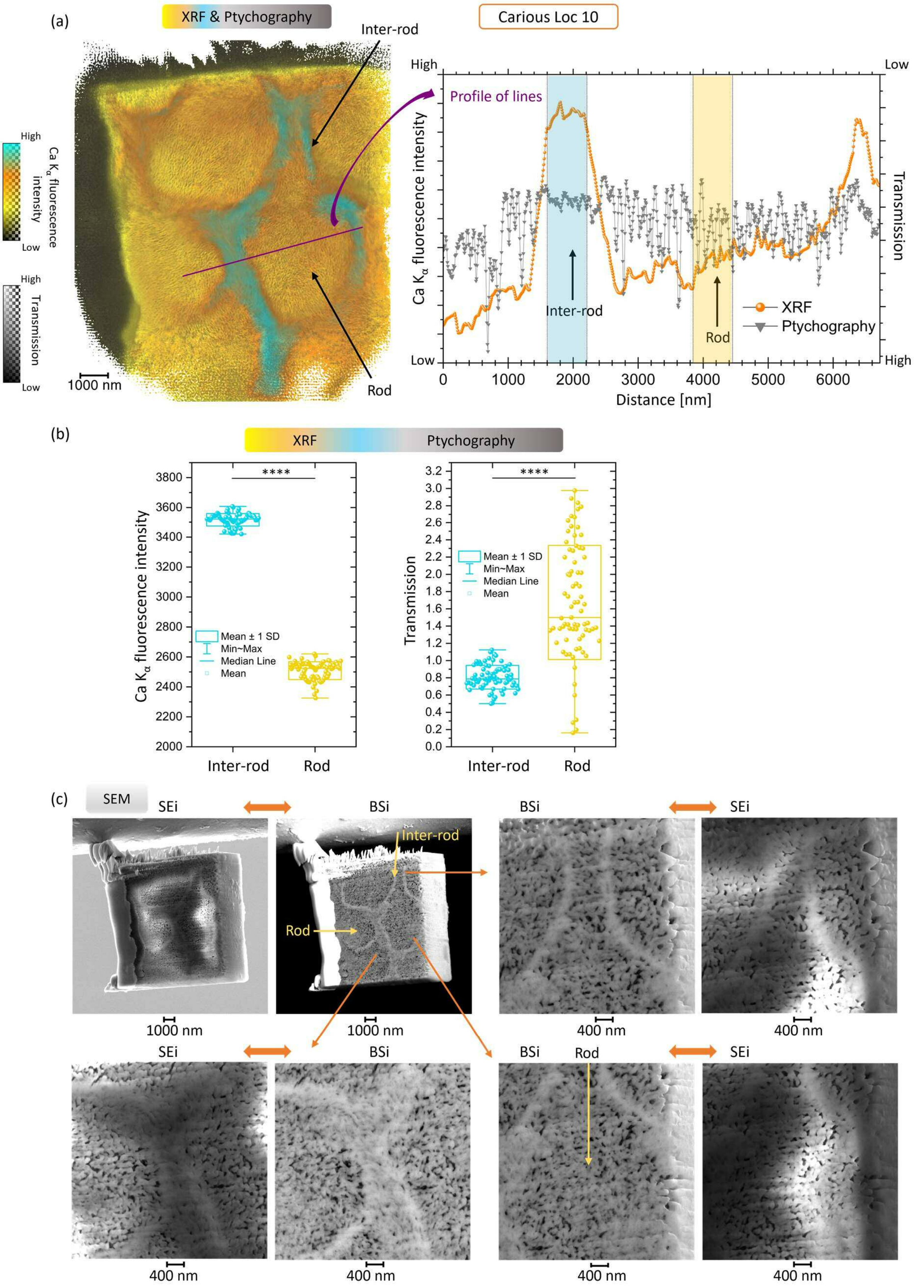

SI-Fig. S36. Correlative structural and chemical analysis of carious enamel Loc 10. Superimposition of XRF map of Ca fluorescence intensity (step of 50 nm), with the ptychography data reconstructed with a pixel size at 8 nm, adapted from[3], and detailed in Figure 5. This highlighted correlative analysis. A plot of the profile of lines extracted from the two datasets, lines in the superimposed

image, showing the correlation in the datasets in the rod and inter-rod. (b) Analysis of the data in the region highlighted in (a) in rod (yellow) and inter-rod (blue) from the XRF and ptychography. Two-sample t-test was carried out. **** represents $p \leq 0.0001$. (c) SEi and BSi of the Loc 10 with zoom in regions in rods and inter-rods showing the enamel structure in the carious region.

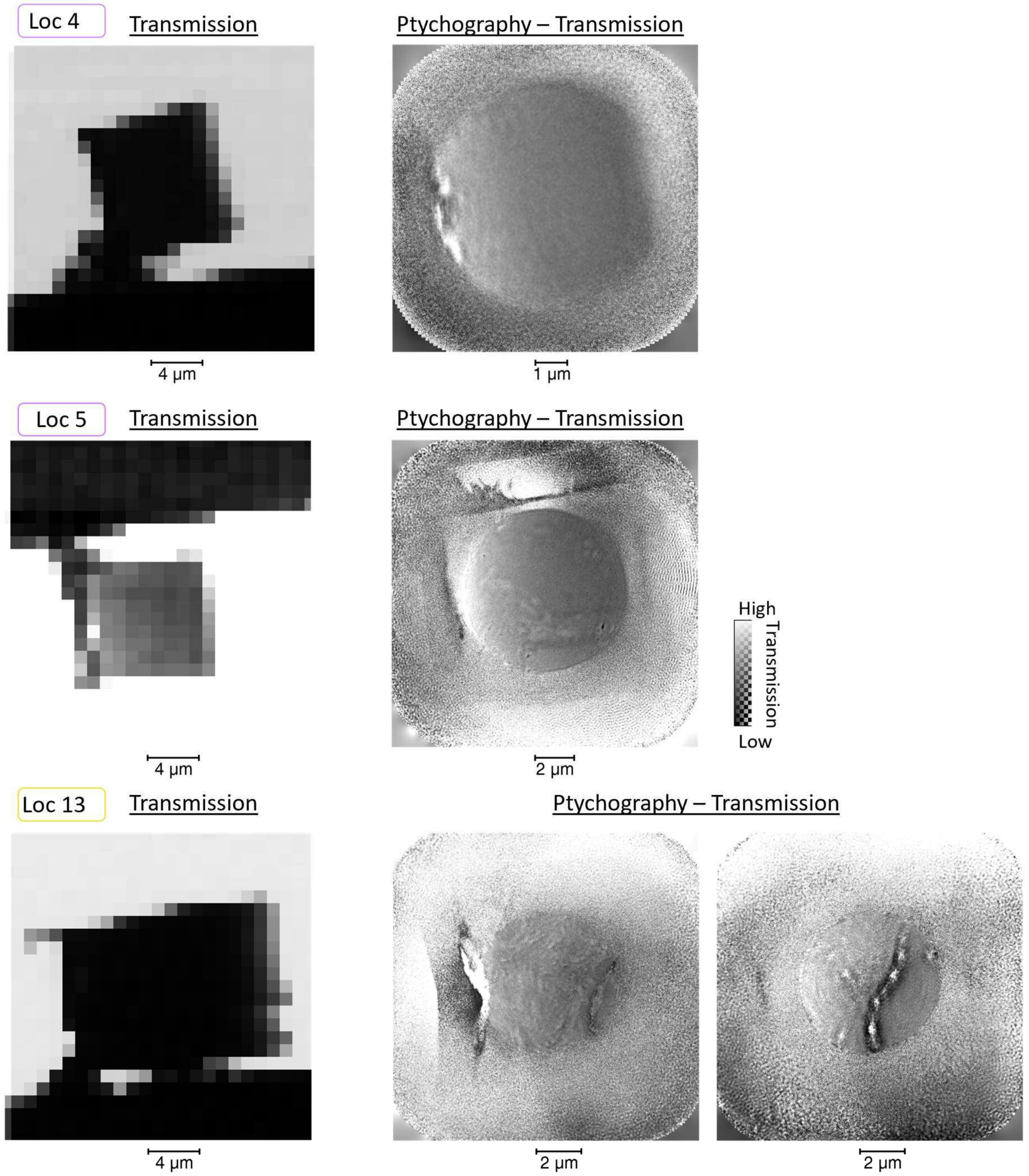

SI-Fig. S37. Soft X-ray ptychography analysis of Loc 4,5,13 using soft X-ray in I08-1 beamline (DLS, U.K.). Image of the transmission results of the scattering data from Loc 4,5,13 and the ptychography details with the amplitude recovered. This highlighted a clear difference with the Loc 10, Figure 5. In these FIB-lamellae, no significant features were seen to reveal the orientation of the crystallites.

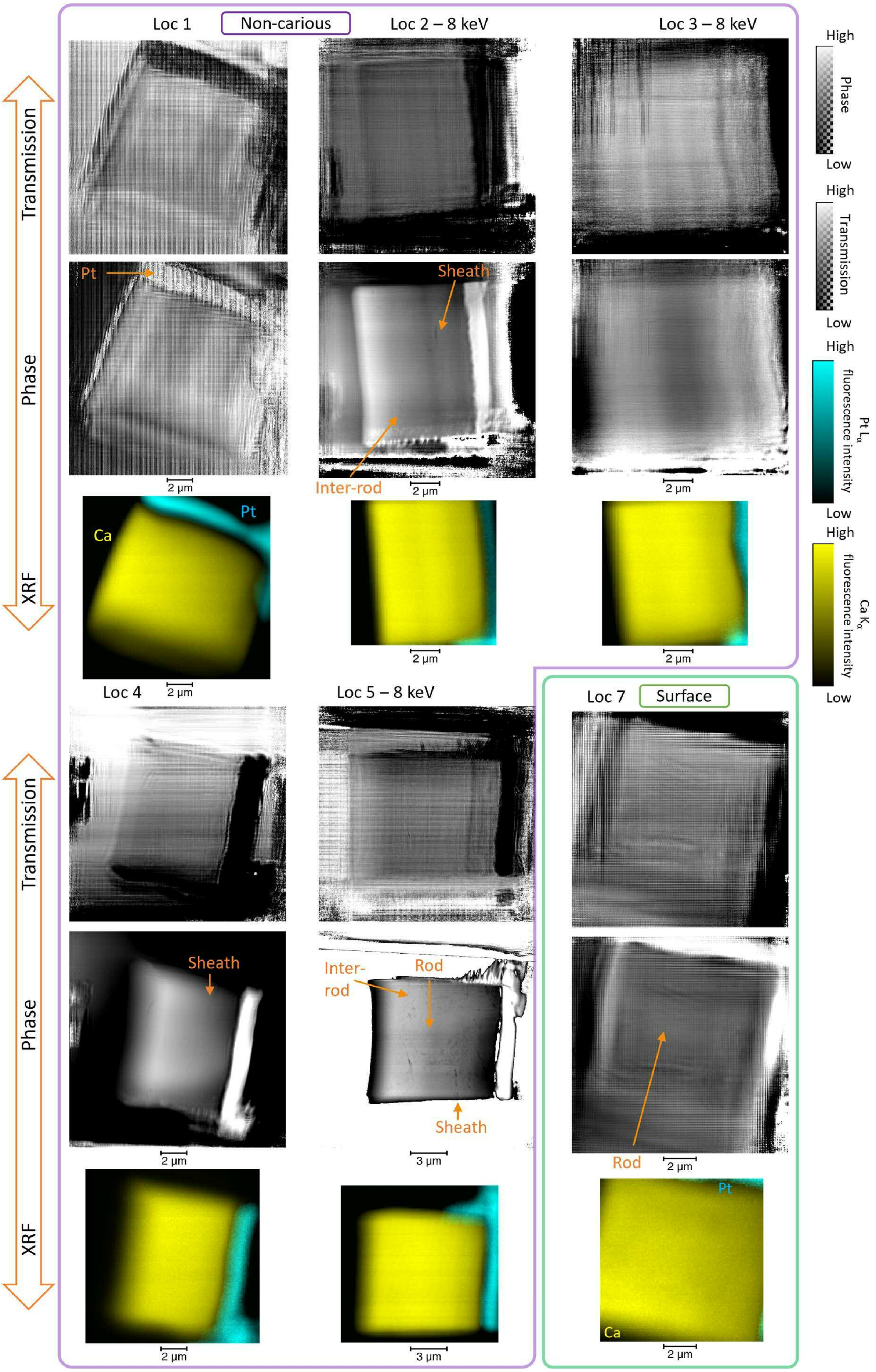
Loc 1
Non-carious
Loc 2 – 8 keV
Loc 3 – 8 keV
Transmission
Phase
XRF
Pt
Sheath
Inter-rod
Ca
Pt
2 μm
High
Phase
Low
High
Transmission
Low
High
Pt L_{α} fluorescence intensity
Low
High
Ca K_{α} fluorescence intensity
Low
Loc 4
Loc 5 – 8 keV
Loc 7
Surface
Sheath
Inter-rod
Rod
Sheath
Rod
3 μm
Pt
Ca

SI-Fig. S38. Hard X-ray ptychography data with the XRF map of Pt and Ca. Transmission and phase image from the reconstructed ptychography data of the location in the non-carious region and surface, acquisition 0.25 s and step of 100 nm. XRF map of Ca from simultaneous acquisition with the ptychography data. The ptychography reconstruction in overall did not show a lot of details, excepted sheath seen) and this was suggested from the sample thickness/density as highlighted with better reconstructions from the carious samples (seen before, and SI-Fig. S39) as well as the sample after demineralisation during the *in situ* analysis (SI-Fig. S28). This will require more optimisation of the analysis. The phase reconstruction were better for hard X-ray regime in comparison to the transmission data, shown for visualisation.

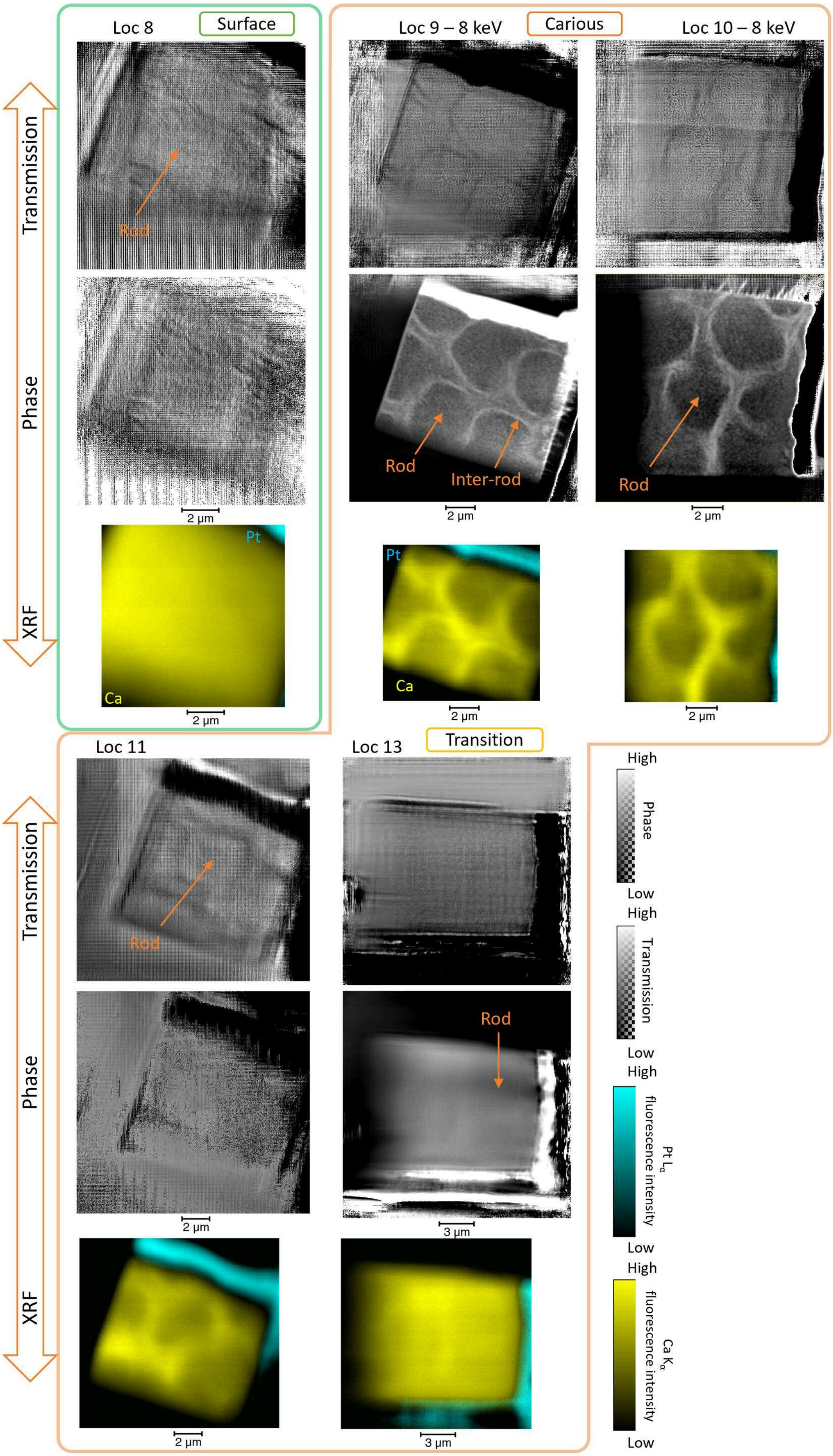
Loc 8
Surface
Loc 9 – 8 keV
Carious
Loc 10 – 8 keV
Transmission
Phase
XRF
Rod
Inter-rod
2 µm
Pt
Ca
Loc 11
Loc 13
Transition
3 µm
High
Low
Transmission
Pt L_α fluorescence intensity
Ca K_α fluorescence intensity

SI-Fig. S39. Hard X-ray ptychography data with the XRF map of Pt and Ca. Transmission and phase image from the reconstructed ptychography data of the location in the surface and carious region and the transition zone, acquisition 0.25 s and step of 100 nm. XRF map of Ca from simultaneous acquisition with the ptychography data.

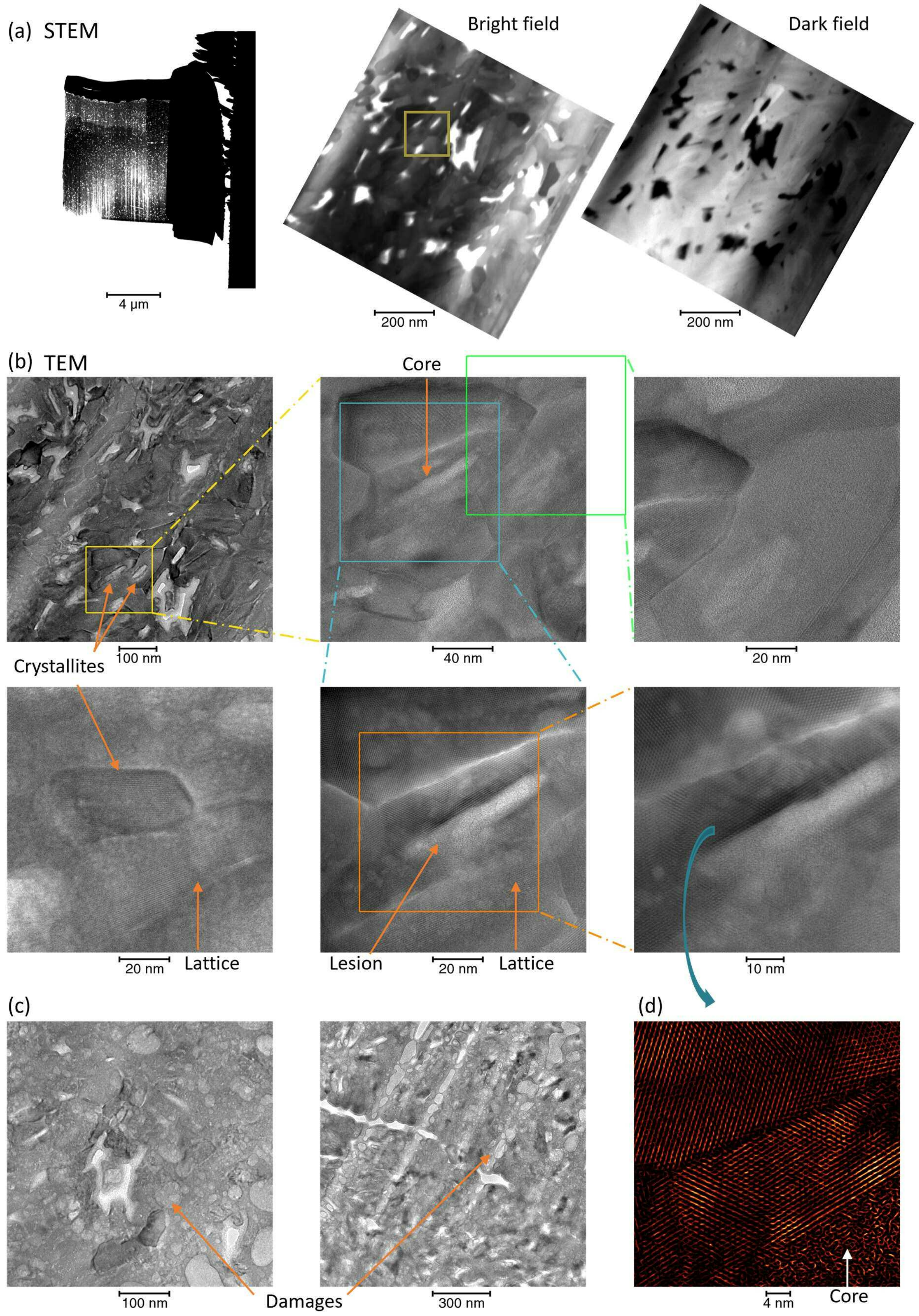

SI-Fig. S40. Electron microscopy analysis of carious enamel. (a) Scanning transmission electron microscopy (STEM) images from a FIB-lamella lifted out from the carious region and the details of

the crystallites, images adapted from[1] with the details of the FIB-lamella and images acquired. (b) TEM analysis of the FIB-lamella with the details of the crystallites, demineralised region and the visualisation of the crystal lattice. (c) TEM images after some exposure to the electron beam already carried out and the observation of damages due to the electron beam. (d) The highlight of the lattice in the crystallites shown using 'membrane enhancement filter' (Avizo) from the image of (b).

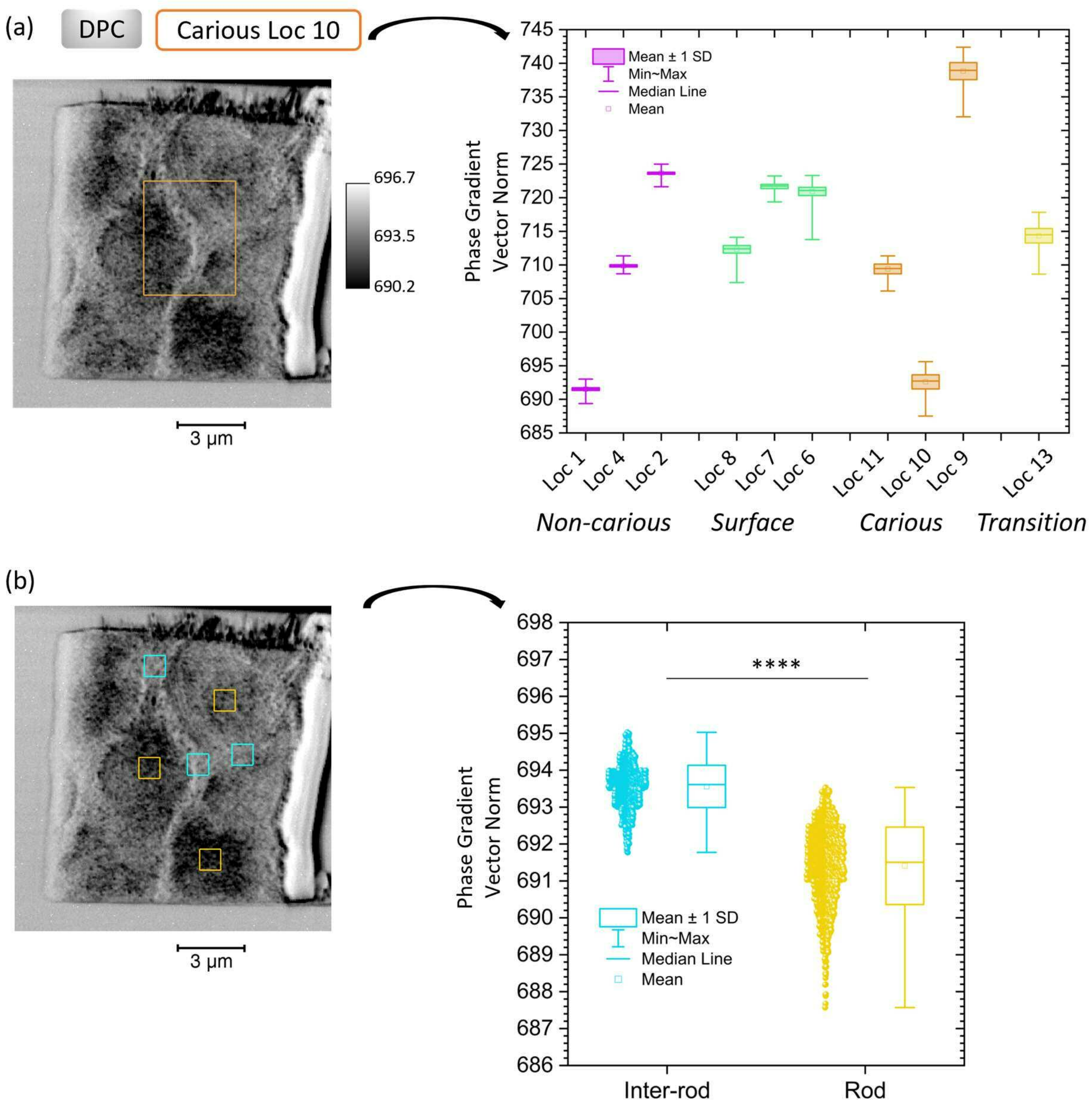

SI-Fig. S41. Analysis of the DPC image acquired in the regions of enamel. (a) DPC image of Loc 10 and statistical analysis of the phase in each pixel acquired in the four regions of enamel from a region of interest (83 × 103 pixel, pixel size 50 nm), highlighted in orange in Loc 10. The standard deviation of the datasets in the carious regions was higher than non-carious suggested from the demineralisation of enamel. (b) Localised analysis of the DPC from rods and inter-rods in Loc 10 illustrated in the DPC image. Three regions in rod and inter-rod were extracted (20 × 20 pixels, pixel size of 50 nm) and each pixel were studied. Two-sample t-test was carried out. **** represents $p \leq 0.0001$.

SI-Table S1. Summary of the different analytical technique used. Description of the techniques used in the correlative analysis of enamel.

Technique	Resolution or Pixel size	Information	Quantitative	Comments
**XRF**	50 nm (limited by beam size).	Chemical information Distribution of elements within the sample	Semi quantitative concentrations if calibration standards used. Relative peak intensities indicate changes for an element concentrations across samples if similar beamline collection conditions are applied.	Differences between the carious region and non-carious region, and surface, and significant differences between rod and inter-rod in the carious Loc 10.
**WAXS/SAXS**	50 nm (limited by beam size).	Phase, crystal structure, q, texture and orientation	Quantitative phase analysis possible when several phases present[4].	Variation of the crystallographic texture in rod and inter-rod. Localised analysis of the orientation of nanostructure. Clear differences in the SAXS scattering in rod and inter-rod.
**DPC**	50 nm (limited by beam size).	Phase image	Quantitative electron density for certain reconstruction conditions[5].	Fast image acquisition revealing structural information in the different regions analysed. Direct correlation with the XRF.
**Ptychography**	Reconstructed dataset performed with a pixel size down to 8 nm and resolution not beam size limited	Phase image or transmission image	The density of the material in certain condition in 3D can be quantified from the phase[6,7].	Nanoscale information about the variation of structure in the enamel in rods and inter-rods in the carious region.
**Tomography**	10x - pixel size was 0.33 µm. Typical best resolution ~0.8 µm and 4x – pixel size was 0.81 µm. Typical best resolution ~1.62 µm.	Imaging technique and 3D visualisation of the sample. This measurement relies on X-ray absorption and X-ray phase contrast mechanism for contrast. Depending on the sample to detector distance(i.e. X-ray propagation distance) it is possible to go from fully absorption to mix of absorption and phase.	For this experiment, a polychromatic beam was used centered around 27 keV ±5 keV. For this reason, the extraction of quantitative values on the thickness or composition becomes harder. However, other quantitative studies can be done where the demineralisation region was extracted or thickness of structure in other study[8] as well as the quantification of the mineralisation using a monochromatic tomography experiment[9]. Quantitative information on the demineralisation obtained post image processing.	Variation in the structure in the different regions. Significant differences in the porosity measured. Overview of the sample, however limited resolution for small details, seen with correlation with SEM.

**FIB-SEM**	Pixel size down to 2.7 nm. FIB and SEM measurement calibration is better than 2% at any magnification.	Topography analysis, contrast analysis from the phase present. FIB for machining sample and revealing subsurface information.	Quantitative details obtained post image processing, porosity in the materials for instance.	Clear variation of structure in the carious, non-carious, surface and transition region. Demineralisation significant in the carious region. Preparation of FIB-lamella for nanoscale experiment.

Movies

Movie S1. Details of the SAXS pattern in several locations of Loc 10. XRF map of Ca intensity with the locations of the SAXS pattern shown. Additional details on the Loc 10 in Figure 4.

References

1 Besnard, C., Marie, A., Buček, P., Sasidharan, S., Harper, R. A., Marathe, S., Wanelik, K., Landini, G., Shelton, R. M. & Korsunsky, A. M. Hierarchical 2D to 3D Micro/Nano-Histology of Human Dental Caries Lesions Using Light, X-ray and Electron Microscopy. *Materials & Design* **220**, 110829, doi:https://doi.org/10.1016/j.matdes.2022.110829 (2022).

2 Harper, R. A., Shelton, R. M., James, J. D., Salvati, E., Besnard, C., Korsunsky, A. M. & Landini, G. Acid-Induced Demineralisation of Human Enamel as a Function of Time and pH Observed Using X-Ray and Polarised Light Imaging. *Acta Biomaterialia* **120**, 240-248, doi:https://doi.org/10.1016/j.actbio.2020.04.045 (2021).

3 Besnard, C., Marie, A., Sasidharan, S., Buček, P., Walker, J., Parker, J. E., Moxham, T. E. J., Daurer, B., Kaulich, B., Kazemian, M., et al. Nanoscale Correlative X-ray Spectroscopy and Ptychography of Carious Dental Enamel. *Materials & Design* **224**, 111272, doi:https://doi.org/10.1016/j.matdes.2022.111272 (2022).

4 Popović, S. Quantitative Phase Analysis by X-ray Diffraction—Doping Methods and Applications. *Crystals* **10**, 27, doi:https://doi.org/10.3390/cryst10010027 (2020).

5 Quinn, P. D., Cacho-Nerin, F., Gomez-Gonzalez, M. A., Parker, J. E., Poon, T. & Walker, J. M. Differential Phase Contrast for Quantitative Imaging and Spectro-Microscopy at a Nanoprobe Beamline. *Journal of Synchrotron Radiation* **30**, 200-207, doi:https://doi.org/10.1107/S1600577522010633 (2023).

6 Diaz, A., Trtik, P., Guizar-Sicairos, M., Menzel, A., Thibault, P. & Bunk, O. Quantitative X-ray Phase Nanotomography. *Physical Review B* **85**, 020104, doi:https://doi.org/10.1103/PhysRevB.85.020104 (2012).

7 Dierolf, M., Menzel, A., Thibault, P., Schneider, P., Kewish, C. M., Wepf, R., Bunk, O. & Pfeiffer, F. Ptychographic X-ray Computed Tomography at the Nanoscale. *Nature* **467**, 436-439, doi:https://doi.org/10.1038/nature09419 (2010).

8 Besnard, C., Harper, R. A., Moxham, T. E. J., James, J. D., Storm, M., Salvati, E., Landini, G., Shelton, R. M. & Korsunsky, A. M. 3D Analysis of Enamel Demineralisation in Human Dental Caries Using High-Resolution, Large Field of View Synchrotron X-Ray Micro-Computed Tomography. *Materials Today Communications* **27**, 102418, doi:https://doi.org/10.1016/j.mtcomm.2021.102418 (2021).

9 Deyhle, H., Dziadowiec, I., Kind, L., Thalmann, P., Schulz, G. & Müller, B. Mineralization of Early Stage Carious Lesions *in Vitro*–a Quantitative Approach. *Dentistry Journal* **3**, 111-122, doi:https://doi.org/10.3390/dj3040111 (2015).
