# Supplementary material for: Multi-resolution Correlative Ultrastructural and Chemical Analysis of Carious Enamel by Scanning Microscopy and Tomographic Imaging
Source: ACS Appl Mater Interfaces. 2023 Jul 31;15(31):37259–73. doi: 10.1021/acsami.3c08031 (PMC10416148; doi:10.1021/acsami.3c08031)
Supplement: Supplementary file 3 — am3c08031_si_003.pdf [file am3c08031_si_003.pdf]

Multi-resolution Correlative Ultrastructural and Chemical Analysis of Carious Enamel by Scanning Microscopy and Tomographic Imaging

Authors

Cyril Besnard*[a], Ali Marie[a], Sisini Sasidharan[a,1], Petr Buček[b], Jessica M. Walker[c], Julia E. Parker[c], Matthew C. Spink[c], Robert A. Harper[d], Shashidhara Marathe[c], Kaz Wanelik[c], Thomas E.J. Moxham[a,c], Enrico Salvati[a,2], Konstantin Ignatyev[c], Michał M. Kłosowski[e], Richard M. Shelton[d], Gabriel Landini[d], Alexander M. Korsunsky[a*]

[a] MBLEM, Department of Engineering Science, University of Oxford, Parks Road, Oxford, Oxfordshire, OX1 3PJ, U.K.

[b] TESCAN-UK Ltd., Wellbrook Court, Girton, Cambridge CB3 0NA, U.K.

[c] Diamond Light Source Ltd., Didcot, Oxfordshire, OX11 0DE, U.K.

[d] School of Dentistry, University of Birmingham, 5 Mill Pool Way, Edgbaston, Birmingham, West Midlands, B5 7EG, U.K.

[e] Research Complex at Harwell, Harwell Campus, OX11 0FA, U.K.

[1] Present address: Department of Materials, Imperial College London, SW7 London, U.K.

[2] Present address: Polytechnic Department of Engineering and Architecture, University of Udine, 33100 Udine UD, Italy

Email addresses:

cyril.besnard@eng.ox.ac.uk, ali.marie@eng.ox.ac.uk, sisini.sasidharan@eng.ox.ac.uk, petr.bucek@tescan.com, jessica.walker@diamond.ac.uk, julia.parker@diamond.ac.uk, matthew.spink@diamond.ac.uk, R.A.Harper@bham.ac.uk, shashidhara.marathe@diamond.ac.uk, kaz.wanelik@diamond.ac.uk, thomas.moxham@eng.ox.ac.uk, enrico.salvati@eng.ox.ac.uk, konstantin.ignatyev@diamond.ac.uk, michal.klosowski@rc-harwell.ac.uk, R.M.Shelton@bham.ac.uk, G.Landini@bham.ac.uk, alexander.korsunsky@eng.ox.ac.uk

* ***Corresponding authors***: Cyril Besnard, cyril.besnard@eng.ox.ac.uk

Alexander M. Korsunsky, alexander.korsunsky@eng.ox.ac.uk

Supporting Information 2 (SI2)

Supporting Note 1

In this section, additional details are reported on the application of nanoprobe.

The synchrotron nanoprobe technique has been widely used. This includes tissues, asteroid samples, nanomaterials, iron, perovskite, freshwater invertebrate, plants, bacteria and cells, etc.[1-22], resolution down to ~15 nm and 40 nm for X-ray absorption near edge structure (XANES) [23]. The mineral was also studied in bones, rat bones[24], and human bone down to 50 nm of beam size[25]. It is important to decrease the resolution of the analysis to examine samples at the scale of the hydroxyapatite (HAp) structure. Each dimension may present a specific organisation and requires further analysis, nanoprobe equipment are required[26]. Combination of X-ray fluorescence spectroscopy (XRF) and X-ray diffraction (XRD) at the nanoscale was previously done to study lithium batteries down to 50 nm, simultaneous analysis[27] and biological sample bone. A beam size of 400 nm, was used to map a bone with XRD and XRF separately in three dimensions (3D) [28]. Cementum was studied with a resolution of 250 nm using XRF and XRD[29]. A lower resolution was done on mice bone with a resolution of 2 µm with XRD and XRF simultaneously, and further going down to 200 nm [30]. Nano-XRD and XRF were used to simultaneously map a bone with a resolution better than 120 nm, where a local variation of signals were generated[31] and 3D computed tomography of these two analyses modes were further reconstructed. This provided the level of details which were used here.

SI2-Table S1. A full summary of the locations and experiments carried out. Summary of the analysis carried out on the sample with the description of the locations characterised and technique and beamline used, I14 and I08-1 in Diamond Light Source (DLS, U.K.) and scanning electron microscopy (SEM). The locations on the sample are shown in Figure 1. Some locations have been previously reported before lift out[32] and the location numbers were updated for this manuscript (referred to as 'Loc paper'). WAXS, referred to as wide-angle X-ray scattering, SAXS for small-angle X-ray scattering, XRF for X-ray fluorescence spectroscopy, DPC for differential phase contrast imaging, FIB for focused ion beam, SEi and BSi for secondary and backscattered electron imaging in SEM. The Loc 10 has been described in[33] with preliminary results.

Loc [32]	Loc paper	Details	Thickness [μm]	I14 (DLS) XRF/WAXS/SAXS /DPC/Ptychography	I08-1 (DLS) Ptychography	SEM SEi/BSi/FIB
	1	Nc	4.27	x		x
	2	Nc	0.562	x		x
	3	Nc	0.935	x		x
3	4	Nc	1.9	x	X	x
4	5	Nc	1.83	x	x	x
5	6	Sur	2.19	x		x
	7	Sur	0.936	x		x
	8	Sur	4.04	x		x
	9	Car	1.78	X		x
10	10	Car	2.43	x	x	x
	11	Car	3.61	x		x
11	12	Car	2.18	x		x
19	13	Tra	4.44	x	X	x
	14	Nc	2.7	x		

Methods

-Sample for nanocharacterisation – Human carious enamel

A human carious enamel was studied where several locations were extracted for nanoprobe characterisations, ptychography, tomography and microscopy analysis. The sample studied had ethical approval obtained from the National Research Ethics Committee; NHS-REC reference 14/EM/1128/ Consortium Reference BCHCDent332.1531.TB. The sample was extracted at the School of Dentistry, University of Birmingham for non-caries related therapeutic reasons. This sample was chosen because it has been initially characterised in previous works showing the variations of structures in the locations of the enamel and had an important carious region. The sample contained non only carious enamel but also surface region and region around the caries. One direction of the sample was perpendicular to the occlusal surface. The conservation of the piece of the tooth allowed additional analyses to be carried out and the development of techniques based on previous screening of the sample Additional information on the enamel sample can be found in[32,34] with light and electron microscopy images and tomography scans previously acquired, as mentioned, the assessment of the caries was carried out by visual inspection by a dentist.
The locations of the enamel used in this study were chosen to have regions in various zones of the enamel, e.g. carious and non-carious region, see Figure 1.

-FIB-SEM imaging and preparation of the locations

Secondary and backscattered electron images (SEi and BSi) were acquired using dual beam focused ion beam (FIB)-scanning electron microscopy (SEM) Tescan Lyra 3 (Tescan, Czech Republic) with a voltage of 5 and 10 keV and FIB with a voltage of 30 keV. The locations analysed are in the continuity of our previous study where cross-sections were made and porosities statistically analysed[32], e.g. Loc 6 in the surface zone, Loc 1 in the non-carious region and Loc 10 in the carious region (body of the lesion and transition zone), with the localisation visualised in Figure 1a and detailed in SI2-Table S1. Some locations were already previously characterised in the study and the correspondence in the number is detailed in SI2-Table S1. The cross-sectional imaging was acquired with SEi and BSi. Porosity analysis was carried out in the locations of the enamel based on the method previously described[32], a region of interest of 230 × 230 pixels (pixel size 6.7 nm) located in the rod was analysed using Avizo (filtering using machine learning procedure[35,36], shadowing correction was applied when required and segmentation of the image leading to binarised images). Then the samples were lifted-out in each location of interest to continue with correlative analysis with nanoprobe analysis on FIB-lamellae using FIB milling. To avoid confusion with the natural structural feature in enamel also called "lamellae" we refer to those as "FIB-lamellae". For the lift out, the samples were attached on copper (Cu) grids following the methods described previously on FIB-lamella[32]. The illustration of the steps for one Location from the milling of the cross-section to the mounting on a holder for I14 nanoprobe (Diamond Light Source) is described in SI2-Fig. S1. In addition to the thinning of the cross-section, FIB milling to create undercuts and then connect to the nanomanipulator was done to carry out transmission electron microscopy FIB-lamella lift-out procedure. Platinum (Pt) was deposited and FIB milling to cut off the sample was done on the side of the location. Then the FIB-lamella was attached on a copper grid with Pt, and detached with a final cut. The thickness of the FIB-lamella was around 1.5 - 4 µm determined with SEM, SI2-Table S1 and SI2-Fig. S2. Several thicknesses were made for the experimental aspect of this study for the test of

several imaging modes as well as different locations in the enamel to have differences in the FIB-lamella with more demineralisation in the caries than non-carious region, with less packed structure. To have the overview of the location of the FIB-lamella on the cross-section prior to lifting and the reference of the structure, a superimposition was carried out from the SEM images acquired, SI-Fig. S2. The analyses of the images were carried out with Avizo software. Samples placed on the grid were mounted on holders for the synchrotron beamline I14 and I08-1 (DLS).

Correlative analysis from SEM and tomography was carried out from the locations. Loc 4, 10 and 11 were imaged after lift-out (trenches remaining and the surrounding regions) and the other locations before the undercut. The overlap from SEM and tomography, 3D rendering, SEi and BSi analysis was carried out using Avizo software based on our previous work[32]. The colour map could be modified for better clarity. Several locations in the carious sample were analysed with a nanoprobe multimodal imaging technique.

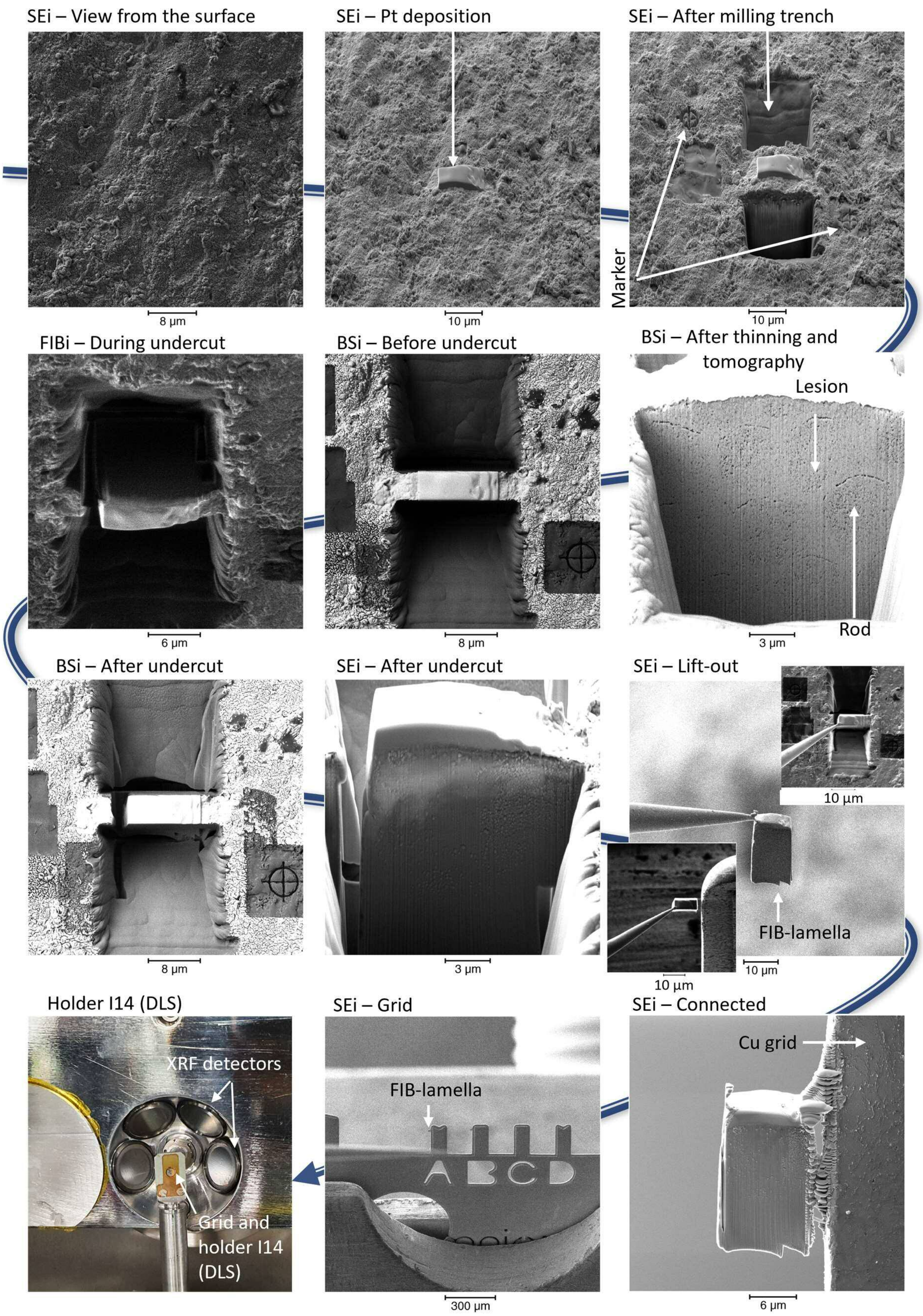

SI2-Fig. S1. Illustration of the steps carried out to prepare samples for nanoprobe with example the Loc 13, thick FIB-lamella. SEM images with BSi, SEi and focused ion beam image (FIBi) of the different steps carried out to prepare the FIB-lamellae attached on Cu grids for the nanoprobe experiment

with at the end the picture of the holder in the beamline I14 (DLS) and the X-ray spectroscopy detector.

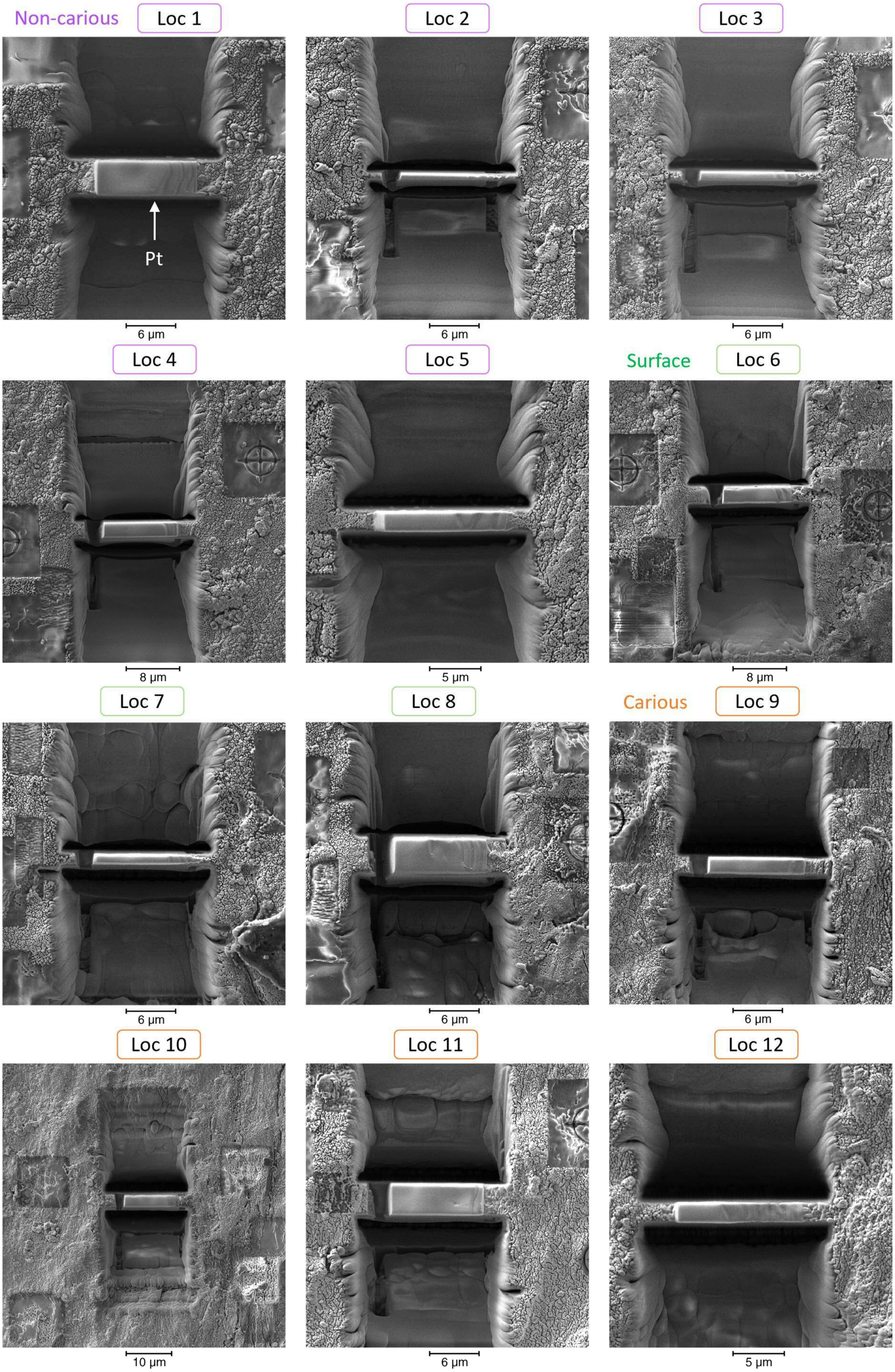
Non-carious
Loc 1
Pt
6 µm
Loc 2
6 µm
Loc 3
6 µm
Loc 4
8 µm
Loc 5
5 µm
Surface
Loc 6
8 µm
Loc 7
6 µm
Loc 8
6 µm
Carious
Loc 9
6 µm
Loc 10
10 µm
Loc 11
6 µm
Loc 12
5 µm

SI2-Fig. S2. SEM analysis of the FIB-lamella before lifting out. SEi of Loc 1 to 12 before lifting out showing the dimension of the FIB-lamella.

-Transmission electron microscopy (TEM)

A FIB-lamella lifted out and analysed previously[32] (referred to as Loc 14 from the carious region) was visualised using transmission electron microscopy JEOL JEM-2100 TEM (JEOL, Japan) with a voltage of 200 keV. A TEM grid was mounted on a TEM holder for the analysis. Enamel FIB-lamella and crystal lattice fringes were imaged. The sample was analysed after exposure to the electron beam to visualise the results of the interaction of the beam on the sample.

-Synchrotron tomography analysis

The sample was imaged using synchrotron tomography analysis in imaging beamline I13-2 [37]. To extend our previous study[32] two additional detector-sample distances were carried out, 25 and 135 mm in addition to the distance of 35 mm (referred to as distance 1, 3 and 2 respectively). Five scans for each distance were acquired to cover the sample. The scans were carried out at room temperature. The full description of the setup, acquisitions, and reconstructions has been previously described. A summary of the setup is described in SI2-Fig. S3a. The data were reconstructed with a voxel size of 325 nm and 0.8125 µm using 10X and 4X objective lenses, respectively. The modification of the distance of the sample led to the variation in the contrast of the reconstruction data with higher details seen for distance 2 and 3. At a further distance, higher contrast is seen from rods to inter-rod but also there is a loss of sharpness in the features, SI2-Fig. S4. This is based on the propagation-based X-ray phase-contrast and agreed with previous work done on dentine[38,39], but the variation of the distance was not yet compared in human carious enamel to the best of our knowledge and is summarised in SI2-Fig. S4 with virtual slices and zoom in regions. The 3D rendering of the regions of the FIB-lamellae was carried out from the dataset acquired at a distance of 35 mm. The rendering of the dataset and the 3D and 2D analyses were based on your previous work[32] and for the 3D rendering, median filtering was carried out. In the locations analysed, Loc 5,10,12 were lifted out prior to the tomography experiment, however, the details of the region were still extracted. A virtual slice after filtering was extracted from the data previously studied using ‘membrane enhancement filer’ which correlates with the optical and SEM image. The analysis, correlation of images were carried out using Avizo software. Porosity analysis was carried out in locations in the carious, non-carious, surface and transition regions. Three subregions of 70 × 52 × 70 pixels (22.425 × 16.572 × 22.425 μm^3) were extracted from each volume of 240 × 240 × 240 pixels, voxel size of 0.325 µm. The segmentation of the low grey value was carried out from the median filtered dataset and the volume fraction was calculated using Avizo. Additional details of the method are described in our previous work[32].

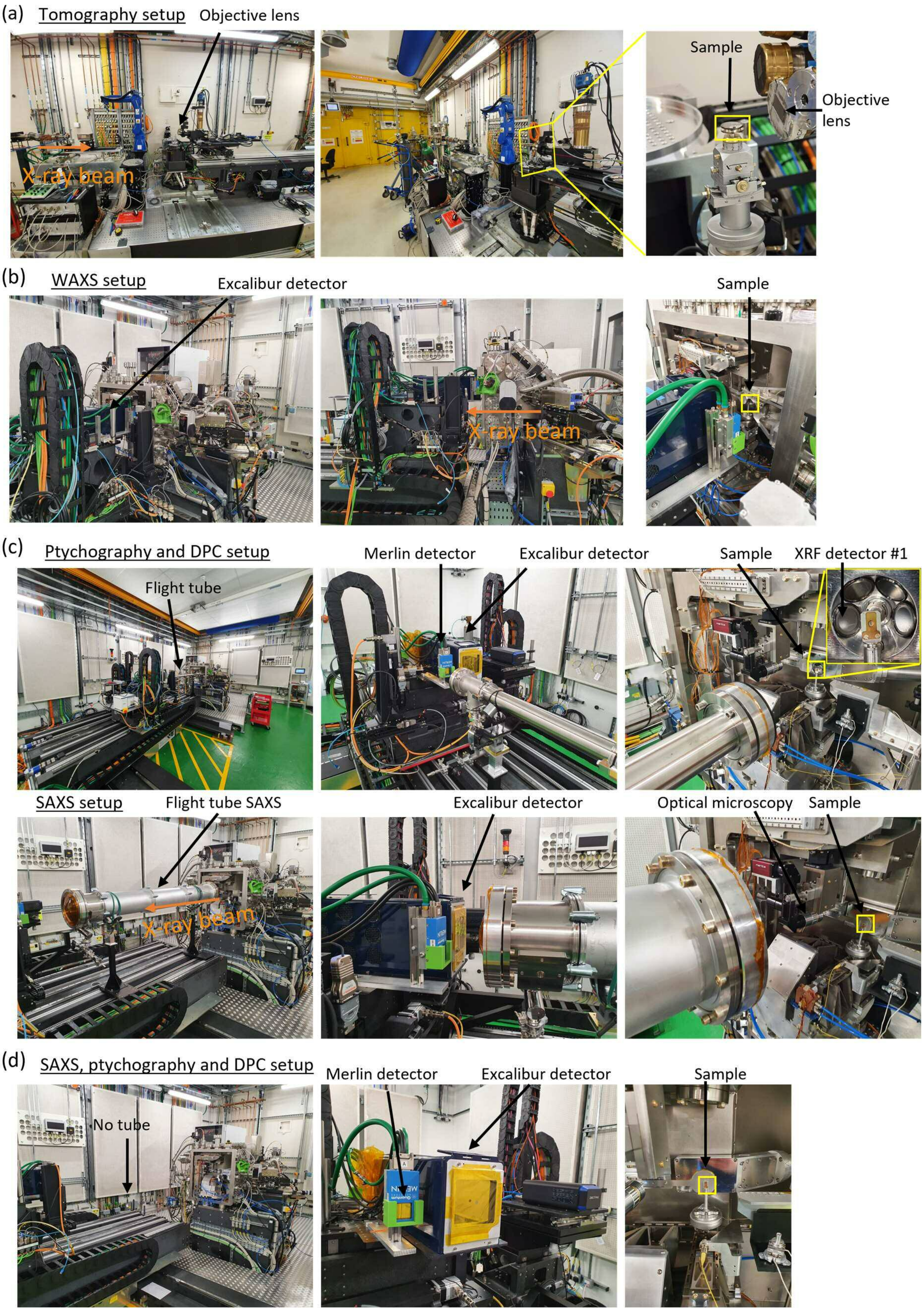

SI2-Fig. S3. Pictures of the setup used for tomography acquisition in I13-2 (DLS) and nanoprobe in I14 (DSL) experiments. (a) Details of the synchrotron tomography experiment carried out in I13-2 (DLS, U.K.) on the carious sample with the view of objective lenses and SEM stub. (b) Details on the

synchrotron nanoprobe I14 (DLS, U.K.) experiments with XRF and WAXS setup, (c) XRF, SAXS, ptychography and DPC with flight tube setup, and (d) technique similar to (c) but without flight tube.

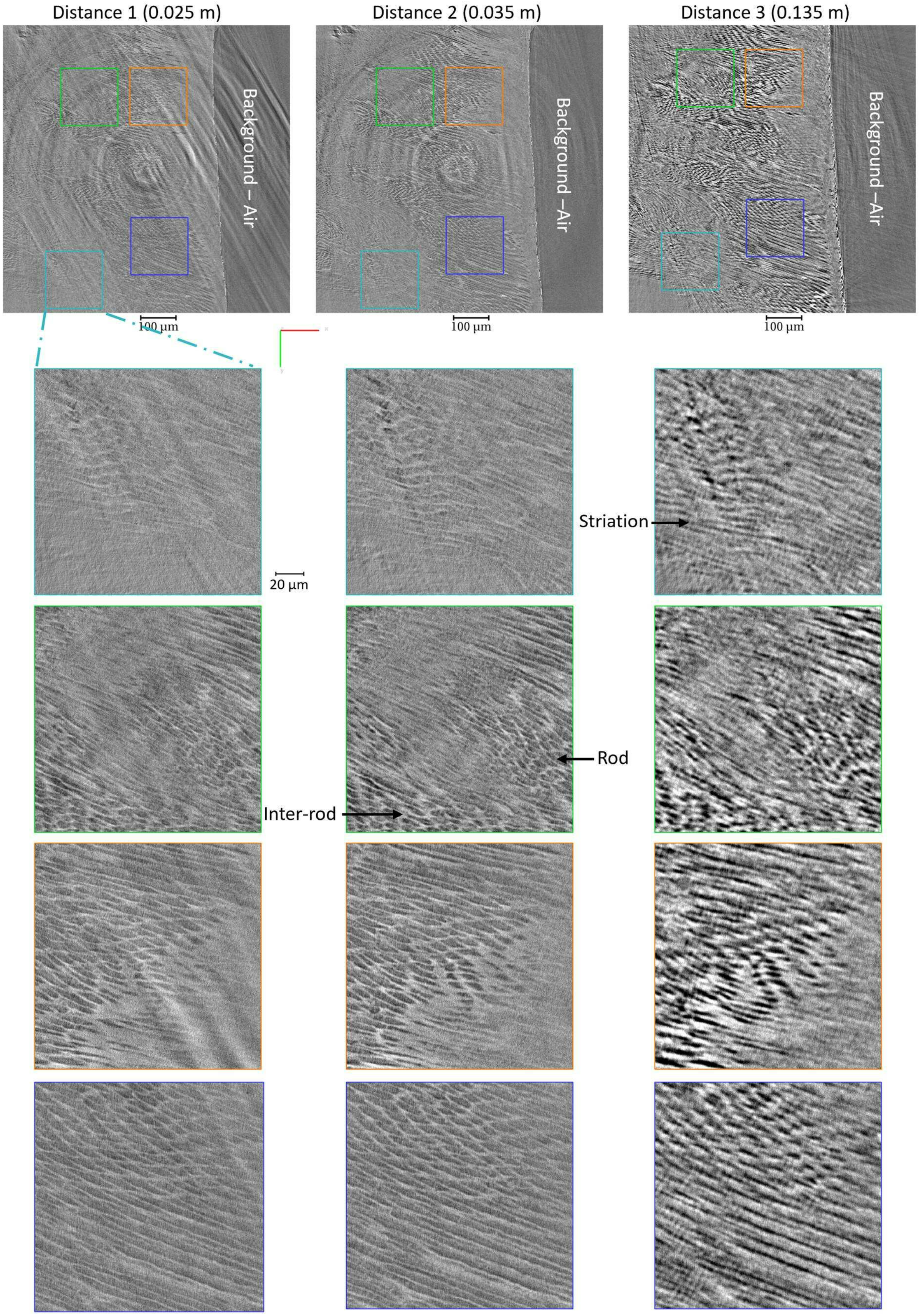

SI2-Fig. S4. Synchrotron tomography analysis of the carious sample mounted on SEM stub. Virtual slices extracted from the reconstructed tomography dataset acquired at three sample-distances. The full dataset for each distance was 815.425 × 815.425 × 685.425 µm and the regions of interest

162.175 × 162.175 µm, voxel size of 325 nm. The virtual slices are from non-filtered datasets. Variation of contrast could be seen as a function of the distance used.

-Nanoprobe I14 XRF/WAXS/SAXS/DPC/Ptychography in dry condition

In the nanoprobe beamline I14 (DLS) [40], preshaped Kirkpatrick-Baez (KB) mirror[41] are used to focus the X-ray to ~ 50 × 50 nm^2 beam size. The sample is raster scanned through the beam and the design of the beamline allows XRF to be acquired simultaneously to each imaging and diffraction mode which were carried out e.g. ptychography, differential phase contrast (DPC) imaging, wide-angle and small-angle X-ray scattering (WAXS and SAXS). Several locations were analysed and are summarised in SI2-Table S1. This corresponds to Cu grids with FIB-lamellae mounted on each one and then placed on the I14 beamline holder. We used the possibility to combine XRF and diffraction mode to provide further details and analysis which can be done either simultaneously or separately. WAXS and SAXS were carried out in several samples and the diffraction signal in both modes was obtained from the scattering over the thickness of the sample. The step size during the analysis and the dimensions of the mappings were a compromise of the time required for the analysis[31].

XRF/WAXS

Local variation in chemical analysis and structural analysis was carried out using synchrotron nanobeam X-ray fluorescence (XRF) and WAXS diffraction experiment mapping acquired at the beamline I14 (DLS)[40,42] using an incident monochromatic beam of energy 18.0004 keV (wavelength of 0.68863 Å). Kirkpatrick-Baez nano-focusing optics are used to provide nano-resolution. WAXS-XRD were acquired with Excalibur 3M - 3 Medipix arrays with 1793 × 2069 pixels, 55 µm pixel size, and a maximum count rate of 1 Mcts/px. Scanning X-ray fluorescence was acquired with a four element Si drift detector screen ~0.6-0.8 sr solid angle, with a maximum count rate of 1.5Mcps per channel, and a 12.5 µm beryllium window. Four detectors are used and the sum of the signal from each detector was analysed (same as when used simultaneously as WAXS, SAXS, DPC and ptychography). The geometry of the analysis was transmission for diffraction and backscatter for XRF. Each analysis was done at room temperature. The pictures of the setup and position of the detector is shown SI2-Fig. S3b,5. Calibration of WAXS was carried out at 18 keV and with standard cerium oxide (CeO_2) for q spacing[43] and for XRF with AXO standard.
Two ranges of beam size were used, in focus (‘high resolution’) 40 × 57 nm^2 and ~71 × 62 nm^2 and 140 × 157 nm^2 and 171 × 162 nm^2 for the analysis unfocused, ‘low resolution’. To change the resolution, the sample was moved out of focus by 75 µm, where the beam size was estimated from a photography reconstruction and beam projection to be approximately 150 x 150 nm^2 highlighting the versatility of the line. These two ranges of beam size will be referred to as focus for high resolution and unfocus for low resolution (and 50 and 150 nm) in this manuscript and acquired each time with a step size of 50 nm and 150 nm respectively. For the WAXS analysis, the sample detector distance was ~243 mm (unfocus distance).

Initial XRF maps were done on each FIB-lamella to locate the FIB-lamella after optical imaging live view (using optical microscopy in the beamline), prior to refining the location of interest. These maps were done with a step of > 150 nm (up to 1 µm) and acquisition of 15 ms using raster and

continuous scan. The integrated intensity of Ca, Cu and Pt from the specific characteristic X-ray lines were extracted in each pixel of the maps and overlapped to display the location, this was carried out by isolating the peak corresponding of elements Ca, Pt and Cu, (without fitting) for the observation of the elements, see SI2-Fig. S5. This provided the accurate position of the FIB-lamella on the grid. A combination of WAXS and XRF with a step of 150 nm, resolution of 150 nm, and acquisition time of 5 s was carried out on regions of interest of several locations. For the experiments, there was a balance between the area which could be scanned and the time considered, for instance, one FIB-lamella could take around 10 h to be acquired with this resolution. A higher XRF resolution map with a step of 50 nm and an acquisition time of 15 ms, was carried out. Here as described previously, this resolution led to a large amount of dataset, as well as time to acquire. With the fast acquisition time, XRF was performed at several locations, however, it was limited for WAXS analysis, a low signal was obtained and the data are not shown. A large region with a low resolution of 150 nm for simultaneous WAXS and XRF analysis was done for Loc 4,5,6,10,13, and zoom in five regions in each location of Loc 4,6,13 was done with a high resolution of 50 nm for both imaging modes. Additional illustrations of the setup, results for one acquired XRF map, one diffraction pattern, the example of XRF spectra from each fluorescence detector acquired for 5 s at 18 keV and the sum of the signal are shown in SI2-Fig. S5. For the XRF experiment, the energy used was able to excite the X-ray lines of Ca, and P as the main elements of HAp, and Cu, Pt were also checked initially for the grid and Pt deposition location, respectively. The data was processed pixel by pixel. From the raw data, background, copper grid, platinum and enamel had distinctive signals displayed in SI-Fig. S5 and SI2-Fig. S6

The analysis of the XRF maps was carried out with Dawn and perspectives in it[44], PyMCa software[45], with the information of each pixel acquired and thus each spectrum, and the map per element from the integrated intensity of it. Several elements were considered based on previous chemical analysis, see SI2-Table 2. Argon (Ar) peak was found from the interaction of the beam with the air. The analysis of the WAXS pattern data was carried out based on our previous works; DAWN and Matlab were used and the fitting of the diffraction peaks was carried out using Gaussian functions[46], SI2-Fig. S5,b. The diffraction pattern was compared to the phase of HAp from the CIF file[47]. In the setting used here and for a thickness of FIB-lamella ~1-4 µm, the signal was low, but some peaks in the diffraction pattern could be identified and analysed. For both the low and high resolution and XRF and WAXS analysis, it was important to check that the signal was exploitable and different from the background and this was confirmed, SI2-Fig. S6a,b, for the diffraction a long exposure was necessary.

The analysis results were plotted with Matlab, and OriginPro software, and imaging analysis and the correlation of the XRF map, WAXS results and SEM images were done with Avizo. Line scan from the XRF data was carried out using Avizo, a step of 50 and 150 nm (pixel size).

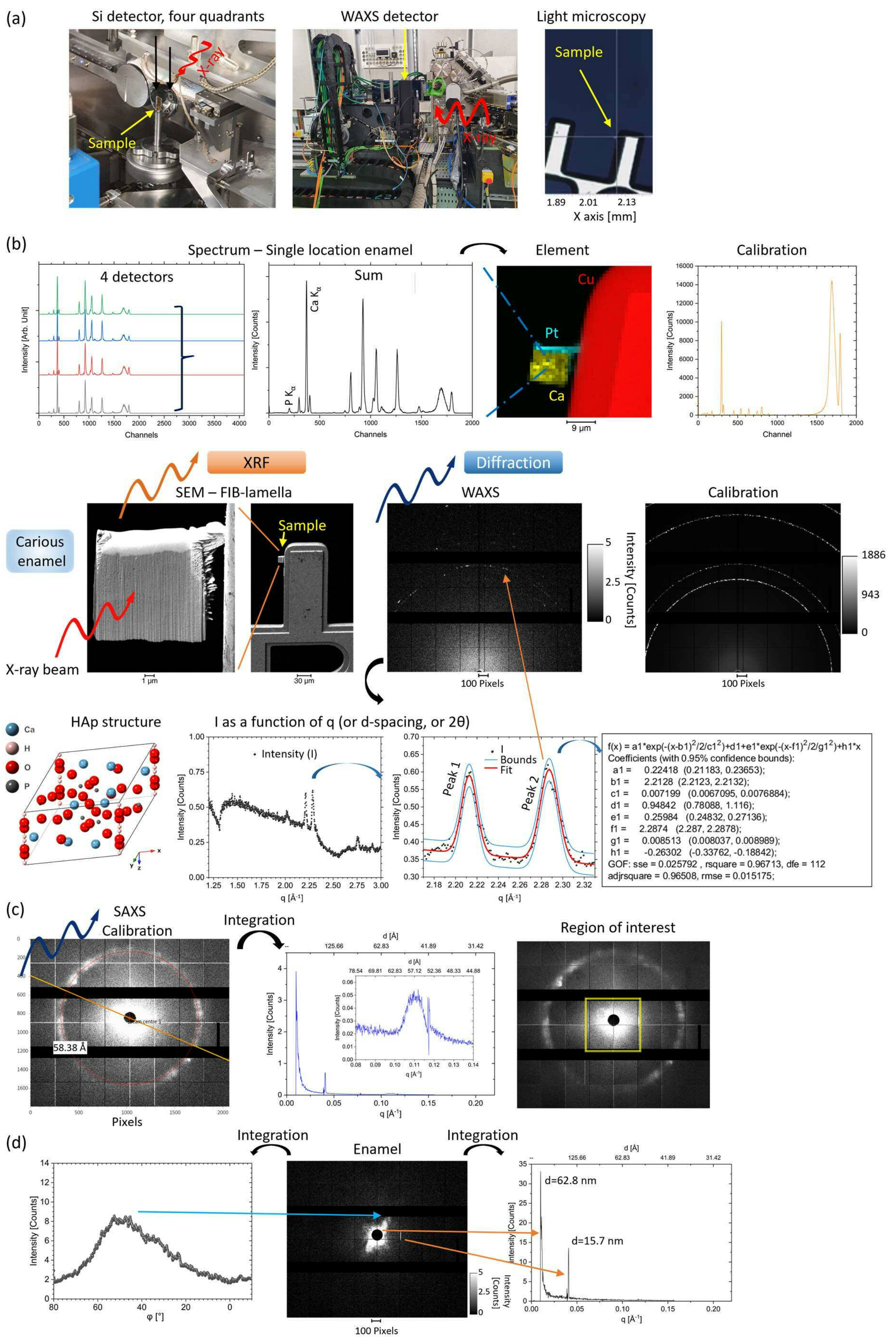
(a)
Si detector, four quadrants
X-ray
Sample
WAXS detector
X-ray
Light microscopy
Sample
1.89 2.01 2.13
X axis [mm]
(b)
Spectrum – Single location enamel
4 detectors
Intensity [Arb. Unit]
Channels
Sum
Intensity [Counts]
Ca Kα
P Kα
Channels
Element
Cu
Pt
Ca
9 µm
Calibration
Intensity [Counts]
Channel
XRF
Diffraction
SEM – FIB-lamella
Sample
Carious enamel
X-ray beam
1 µm
30 µm
WAXS
100 Pixels
Intensity [Counts]
Calibration
1886
943
0
100 Pixels
HAp structure
Ca
H
O
P
I as a function of q (or d-spacing, or 2θ)
Intensity (I)
Intensity [Counts]
q [Å⁻¹]
I
Bounds
Fit
Peak 1
Peak 2
Intensity [Counts]
q [Å⁻¹]
f(x) = a1*exp(-(x-b1)^2/2/c1^2)+d1+e1*exp(-(x-f1)^2/2/g1^2)+h1*x
Coefficients (with 0.95% confidence bounds):
a1 = 0.22418 (0.21183, 0.23653);
b1 = 2.2128 (2.2123, 2.2132);
c1 = 0.007199 (0.0067095, 0.0076884);
d1 = 0.94842 (0.78088, 1.116);
e1 = 0.25984 (0.24832, 0.27136);
f1 = 2.2874 (2.287, 2.2878);
g1 = 0.008513 (0.008037, 0.008989);
h1 = -0.26302 (-0.33762, -0.18842);
GOF: sse = 0.025792 , rsquare = 0.96713, dfe = 112
adjrsquare = 0.96508, rmse = 0.015175;
(c)
SAXS
Calibration
58.38 Å
Pixels
Integration
d [Å]
Intensity [Counts]
q [Å⁻¹]
Region of interest
(d)
Integration
Enamel
Integration
Intensity [Counts]
φ [°]
100 Pixels
Intensity [Counts]
d [Å]
d=62.8 nm
d=15.7 nm
q [Å⁻¹]

SI2-Fig. S5. Schematic and illustration of the setup and analysis of the synchrotron nano-XRF, XRD and SAXS data in I14 (DLS). (a) Setup of the experiment with the silicon (Si) detector and the sample's position. (b) BSi of one FIB-lamella, Loc 10, and illustration of the XRF spectra from each detector and the sum acquired on one pixel with the overlap of the energy of the elements Ca, Pt, and Cu and the spectrum from the AXO standard (18 keV). Illustration of the WAXS pattern of the calibrant CeO_2 (acquired at 18 keV, unfocus and the first Debye-Scherrer ring at d-spacing of 3.12 Å) and from the sample and the 1D plot intensity (referred to as I) as a function of q, fitting of the peak 1 and 2 reported in the manuscript and the HAp structure, CIF file 203027 Inorganic Crystal Structure Database (ICSD) [47,48]. CrystalMaker® (CrystalMaker Software Ltd, Oxford, England) was used for the representation of the crystal structure. (c) Illustration of the SAXS pattern from the calibrant AgBe using DAWN software (average of the patterns acquired using 12 keV and unfocus) showing the first ring at d-spacing 58.38 Å [49], and a region of interest which is used in the analysis of the SAXS pattern acquired on the enamel. 1D plot of the intensity as a function of the q spacing from the calibrant showing the distance acquired and zoom in the q range (0.08 to 0.014 $Å^{-1}$) from 0 to 5 ° anti-clockwise. (d) SAXS pattern from the enamel Loc 10 and integration of the data. 1D plot of the intensity as a function of the q spacing for 0 to 5 ° to highlight the dimension studied and 1D plot of the intensity as a function of the azimuthal angle φ (this was done from -10 to 80 ° and it covered the q range 0.009 to 0.039 $Å^{-1}$) for the orientation[50].

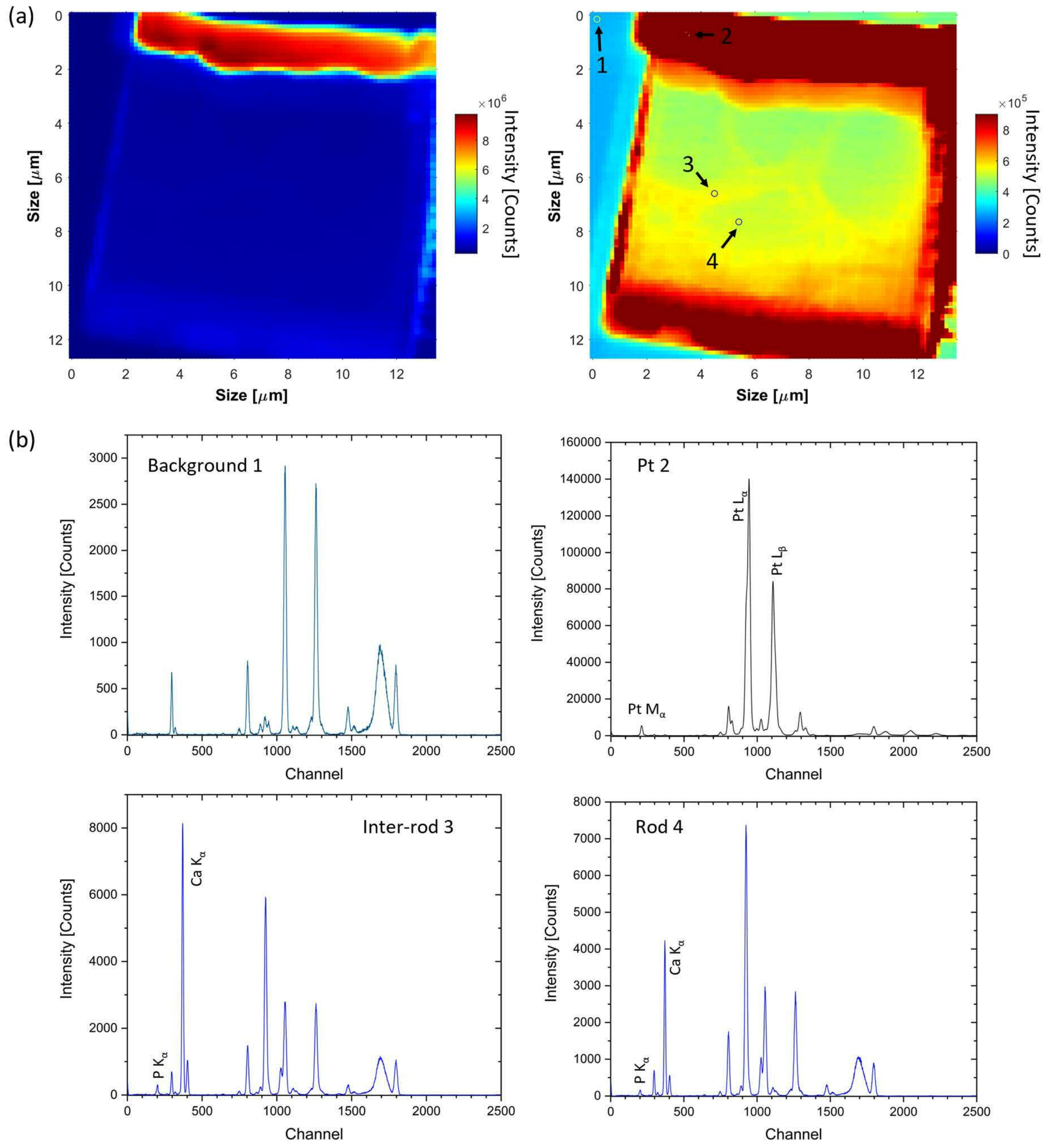

SI2-Fig. S6. XRF analysis of the Loc 10, raw data. (a) Map of the sum of the signal from each detector, resolution 150 nm, and acquisition time 5 s, with the highlight of four locations. (b) XRF spectra of the four locations, background, platinum deposition, rod and inter-rod.

XRF/SAXS

SAXS experiment was carried in I14 (DLS) on several samples using an incident monochromatic beam of energy 12.0004 eV, two sessions were carried out, with and without flight tube. The setups were similar to the wide-angle experiment, with initially microscopy and XRF map to locate the sample and then refinement of the location was carried out. The preliminary fast XRF scans were acquired for each sample (exposure 0.015 ms, rough step in x and y e.g. 1 µm, and map size 20 × 20 µm) to

locate the FIB-lamellae and then refinement was done for the map. Ca, Pt and Cu were used to locate the sample on the Cu grid. Beam sizes used for experiments were the same 55 x 45 nm^2 when in focus and ~150 × 150 nm^2 when it is out of focus and are referred to as 50 and 150 nm respectively in the manuscript. SAXS was carried out in transmission mode and the diffraction patterns were recorded with Excalibur 3M - 3 Medipix arrays consisting of 1793 × 2069 pixels, 55 µm pixel size a maximum count rate of 1 Mcts/px and the distance sample detector of 2.21 m (unfocus distance). Maps were acquired with a step of 50 nm (x and y) in focus and 150 nm out of focus (for demagnification the sample was moved from 0.075 mm along the beam) and the acquisition time was mainly 5 s (10 s and 15 s were also used but only for a few data). Silver behenate was used as standard for SAXS during the experiment. Using 5 s exposure for SAXS and XRF, high resolution maps were carried out in region of interests (rods, inter-rods) and the low resolution on the other hand provided large area on the FIB-lamellae. This was also required because within the time access, it was not possible to scan the full FIB-lamella with the highest resolution for XRF/SAXS. XRF map at high resolution was also done with 15 ms exposure which provided the locations for the region of interest. For the XRF analysis, see section XRF/WAXS. Similar as the WAXS pattern, the signal from the scattering was checked to confirm that the data can be studied, and this was demonstrated for both resolution, see SI2-Fig. S7c-f. SAXS was carried out with and without flight tube, and in both setup the scattering could be seen, SI2-Fig. S8 and the setup is described in SI2-Fig. S2. Calibration of the SAXS distance was carried out using silver behenate (AgBe), SI2-Fig. S5c.

For the analysis, the assessment of the X-ray beam damage in localised regions after several exposures to the beam was checked on two locations, Loc 2 and Loc 4, using XRF and SAXS. Same location was exposed to 10 or 5 s in high and low resolution with both XRF and SAXS, and then XRF map was acquired over a larger area, this was repeated 5 times, and the results are present in SI-Figs. 22,23. XRF maps prior to the iterative procedure with also map at low resolution acquired. XRF map of Ca after the iterative procedure was also acquired. The damage to samples was accessed to confirm the possibility to analyse the material. No formation of holes of dimension of the resolution used was found in the sample based on the XRF imaging after the first iterations. This was checked in two FIB-lamellae of different thickness. This confirms that what was seen between the carious and normal enamel was dependent on the sample itself. DPC was carried out in parallel to observe the structure of the locations.

The SAXS pattern were analysed using DAWN [44], Fiji, ImageJ [51,52] and Avizo. Extracted pattern were visualised and variation in the scattering pattern was noticed in different locations.

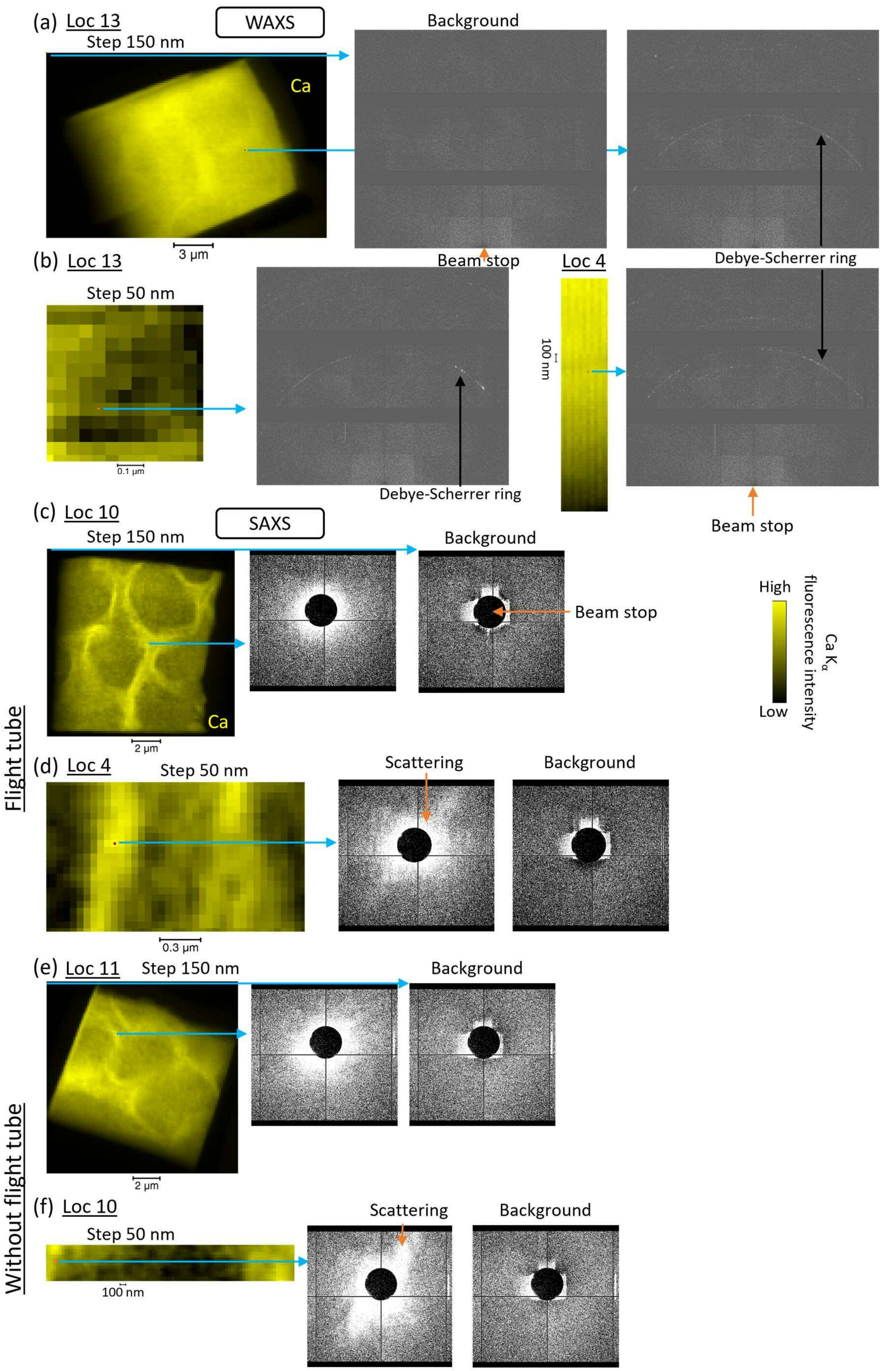
(a) Loc 13
Step 150 nm
WAXS
Background
Ca
3 µm
(b) Loc 13
Step 50 nm
0.1 µm
Beam stop
Loc 4
100 nm
Debye-Scherrer ring
Debye-Scherrer ring
Beam stop
(c) Loc 10
Step 150 nm
SAXS
Background
Beam stop
Ca
2 µm
High
Low
Ca K_{α} fluorescence intensity
Flight tube
(d) Loc 4
Step 50 nm
Scattering
Background
0.3 µm
(e) Loc 11
Step 150 nm
Background
2 µm
Without flight tube
(f) Loc 10
Step 50 nm
100 nm
Scattering
Background

63

SI2-Fig. S7. XRF, WAXS and SAXS analysis with the XRF map and extracted diffraction patterns showing the feasibility of the measurements. (a) XRF map of Ca with a step of 150 nm, exposure of 5 s and extracted WAXS pattern from one pixel in the sample and one outside, energy 18 keV. (b) High resolution XRF maps of Ca and extracted WAXS pattern from one pixel in the sample in each map acquired, step 50 nm, exposure 5 s, energy 18 keV. (c) XRF/SAXS low resolution map with Ca is intensity shown, and extracted SAXS patterns from the sample and the background (pixel outside the sample), step 150 nm, exposure of 5 s, with flight tube, energy 12 keV. (d) High resolution XRF/SAXS map with Ca intensity shown and extracted SAXS pattern from the sample, and from a pixel in the background, step 50 nm, exposure of 5 s, with a flight tube, energy 12 keV. (e,f) Similar illustration as (c,d) but the experiment was carried out without a flight tube. These analyses highlight the feasibility to obtain the XRF signal, WAXS signal with Debye Scherrer ring and SAXS signal with the observation of scattering with high and low resolution. The images of SAXS patterns illustrated were cropped from the full detector image acquired (Fiji/ImageJ dimension(741, 615, 576, 561)).

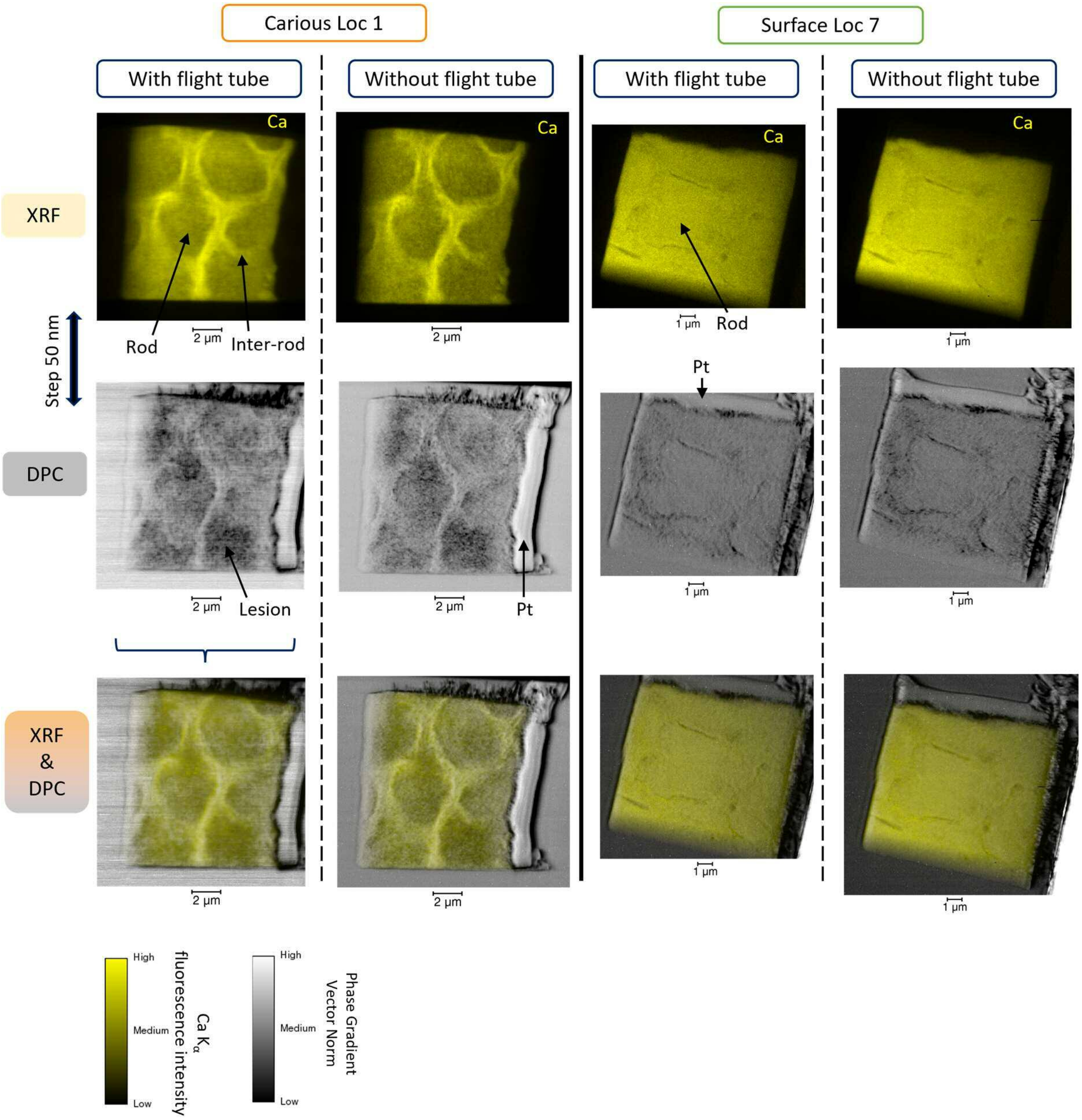

SI2-Fig. S8. Comparison of the XRF spectroscopy analysis, DPC and ptychography with and without flight tube in I14 (DLS). Loc 1 and Loc 7 were analysed with and without a flight tube, and a XRF map of Ca intensity, DPC and ptychography with the corresponding XRF map are detailed. In both setups, the intensity obtained was enough to carry out the different modes. For XRF and DPC, a step of 50 nm in high resolution, for XRF simultaneously as ptychography a step of 100 nm was used and the resolution for XRF was ~1 µm. Energy of 12 keV for Loc 1 and Loc 7 without flight tube, and 8 keV for Loc 1 with flight tube.

XRF/DPC

Various locations in the carious, non-carious and surface were characterised by X-ray differential phase contrast imaging method simultaneously as the XRF. DPC is based on the index of refraction[53] and there was an enhancement of the features from this variation. DPC was carried out in transmission geometry, using a distance of 3 m from detector Quad Merlin (1806 × 1548 pixel, 55 µm pixel size, area 113 × 100 mm, max count 1 Mcts/px, frame rate 100 Hz) to the sample. The step size was 50 nm (in x and y) and the exposure time per pixel of 15 ms. The beam size was 55 × 45 nm^2, and similar to SAXS experiment, the two sessions were with the same bean size. The reconstruction of the DPC data was carried out using I14 beamline in-house script with the incorporation of phase retrieval and determination of the gradient normalisation. From the benefit of the beamline, XRF was simultaneously acquired at each position leading to correlative analysis with a fast chemical and imaging map, around 20 min for full FIB-lamella at high resolution. For the analysis of the XRF data, see section XRF/WAXS. The energies used were 8 keV and 12 keV. DPC was previously used for dental in particular dentine with the observation of the tubules[54,55] and not yet reported in enamel at nano resolution. The setup of the different techniques is detailed in SI2-Fig. S2.

XRF/Ptychography

Based on the setup for DPC, 2D ptychography in transmission geometry was carried out by moving the sample in a defocus position of 0.8 mm (equivalent to a beam size of around 1 µm on the sample). Then the maps were acquired with a step size of 100 nm (in x and y), exposure of mainly 0.25 s (0.1 s was also used) per point and the acquisition of the diffraction patterns was carried out using the detector Quad Merlin similar to DPC. XRF was also acquired simultaneously but with a resolution of around 1 µm. The energies used were 8 keV and 12 keV. The 2D ptychography reconstruction was performed with a pixel size of ~15.96, 23.94 and 31.92 nm. The data were analysed with DAWN and Avizo.

Thus, we used the flexibility in the beamline to change from one setup to another one. Translation in z along the beam moved the sample in and out of focus to obtain demagnification by changing the distance from sample to detector. Translational stage in x to move from one detector to another. The data obtained in the different modes contain a vast amount of data which required to be recorded almost instantaneously at each position acquired. This was possible with the presence of high hardware to record the data, saved by keeping the motion of the samples. In terms of data, this could correspond to 80000 points in the case we had. The possibility to change between detectors and thus mode without removing the sample allowed us to carry out a correlative analysis. SAXS,

DPC and ptychography were carried out with and without a flight tube (an evacuated tube which is used to reduce the air scattering). The removal of the flight tube allows switching between the Merlin and Excalibur detectors in a faster way. The overall summary of the different experiments carried out is shown in SI2-Fig. S3.

Based on the analyses in a dry condition and the possibility to combine analytical methods, preliminary tests were done using a liquid cell in I14 beamline. The liquid cell was DENSsolutions (Netherland) sample environment cell and was adapted for liquid flow using the same nano chips and a syringe pump as a previous study from Parker J.E. et al. [56].

-Nanoprobe – Analysis in liquid

FIB-SEM preparation

For the liquid cell, a FIB-lamella taken from the non-carious region of the sample was used, Loc 14. The milling of the FIB-lamella was performed following the same method as the FIB-lamella characterised in the dry conditions attached to Cu grids. However, in this case, the FIB-lamella was not mounted on the Cu grid but on a DENS heating chip (DENSsolutions, Netherlands). FIB-SEM and BSi were used to prepare and image the FIB-lamella, before the lift-out step, using the same equipment as previously described. For the lift-out and placement of the FIB-lamella on the chip, FIB-SEM JEOL JIB-4700F (JEOL, Ltd., Japan) equipped with a nanomanipulator (Kleindiek, Germany) was used. SEM imaging was done at 15 keV and FIB imaging and milling at 30 keV. The nanomanipulator equipped with a needle was used to perform the lift out and then rotate (roll) the sample by 90 °. Then the FIB-lamella was attached to the chip using tungsten and then separated from the needle using the ion beam. The cutting was carried out with a current of 3000 pA and the deposition with 300 pA. The sample preparation details are described in SI2-Fig. S9. The remainder of the FIB-lamella after the synchrotron experiment was visualised with SEM Lyra 3 described previously.

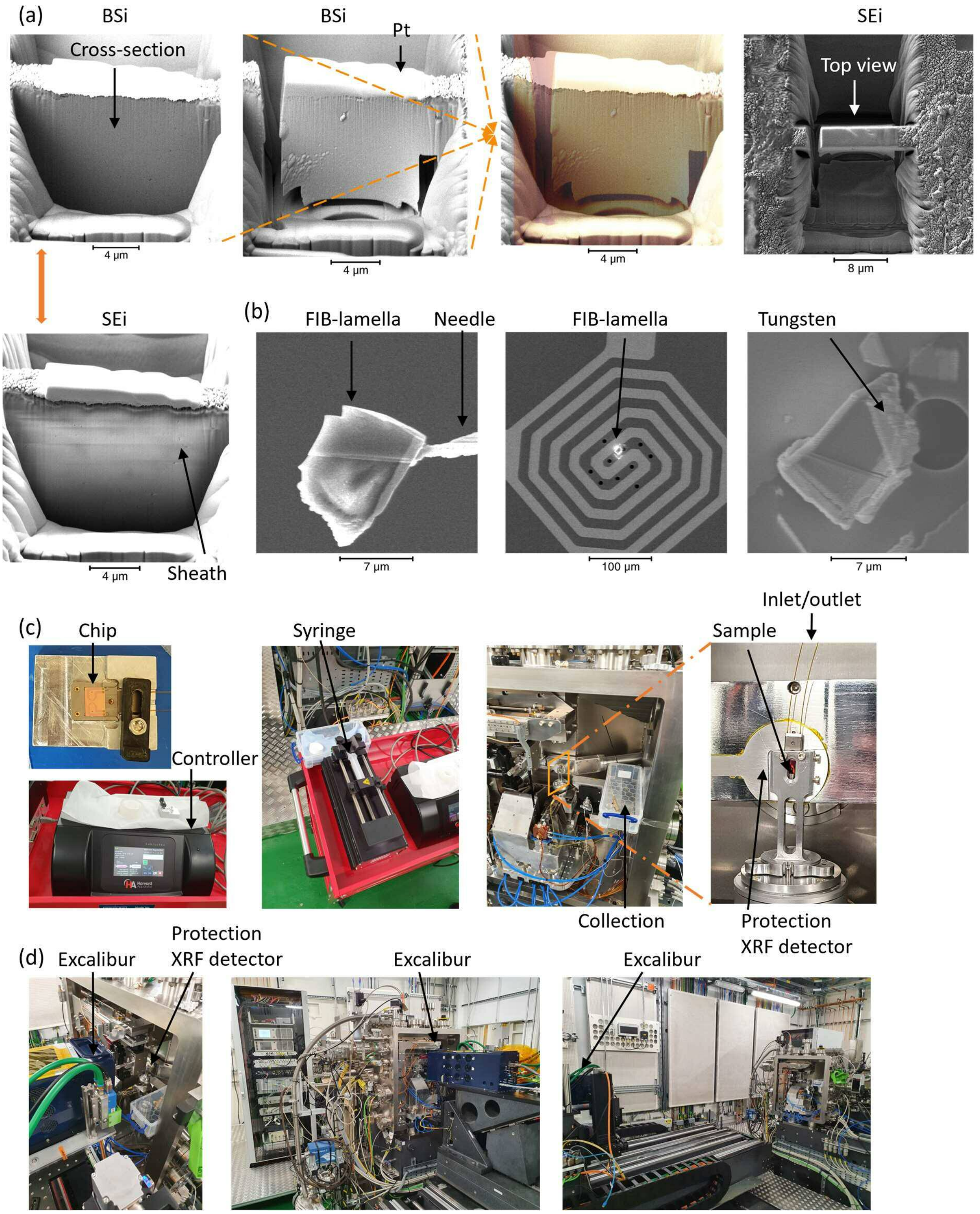

SI2-Fig. S9. SEM preparation and setup of the *in situ* experiment on a non-carious FIB-lamella in I14 beamline (DLS, U.K.). (a) SEM images of the FIB-lamella from the cross-section to the undercut step, with SEi and BSi, superimposition of the BSi of the undercut and cross-section. (b) SEi of the FIB-lamella attached to the needle and fixed on the chip with tungsten deposition. (c) Picture of the chip, syringe, controller, the position of the chip, the cover to protect the XRF detector in the I14 beamline (DLS, U.K.) and inlet and outlet position for the flow of liquid. (d) Pictures of the setup for WAXS and SAXS and the detector positions.

Nanoprobe

The chip was placed on DENS holder with tubes for the inlet and outlet connected to the syringe pump and container to collect solutions respectively. This assembly was positioned in the beamline, see SI2-Fig. S9 for the pictures of the setup. Initially, the analyses were carried out in a dry state. XRF and WAXS were carried out with a beam size of 55 × 45 nm^2, XRF was done at 0.015 ms and the combination of XRF/WAXS with exposures of 5 s and 10 s. High resolution maps in focus (50 nm step) were carried out on small regions and low resolution out of focus (step 150 nm) were performed to cover the large area of the FIB-lamella. Following the initial condition, deionised water was flowed from the syringe to the sample with a flow rate of 5 $\mu L.min^{-1}$. XRF with fast acquisition was carried out and WAXS on a small region, both in focus and out of focus were carried out with an acquisition time of 10 s. The energy used was 18 keV. The setup was then modified with SAXS, DPC and ptychography setup following previous experiments and with an energy of 12 keV, see SI2-Fig. S8 for the setup. The beam size was 55 x 45 nm^2. Deionised water was flowed for a few hours following which acetate buffer (2.2 mM calcium, 2.2 mM phosphate, 75 mM acetate at pH 4.3) was flowed[57,58] and finally artificial saliva (AS) (0.7 $mmol.L^{-1}$ $CaCl_2$, 0.2 $mmol.L^{-1}$ $MgCl_2$, 4.0 $mmol.L^{-1}$ KH_2PO_4, 30.0 $mmol.L^{-1}$ KCl, 20.0 $mmol.L^{-1}$ HEPES at pH 7) [59]. Time-lapse of XRF/DPC was carried out with in-house script. For the interpretation of the data from the different modes, see the previous sections. Two regions were extracted from a series of XRF maps and the intensity was plotted with time.

-I08-1 Soft X-ray ptychography

The soft X-ray ptychography experiment with X-ray coherent diffractive imaging (CDI) was performed in the I08-1 beamline (DLS). The same holders used for I14 (DLS) with samples mounted on Cu grid were used for X-ray 2D soft ptychography, coherent diffractive imaging method. The sample was positioned in the line using the same holder as I14 nanoprobe experiment which benefited from a direct localisation of the sample and ROI analysis). Four FIB-lamellae were tested, two in the normal region Loc 4,5 one in the transition region Loc 13 and one in the carious region Loc 10 which was preliminarily described in[33]. The beam was ~790 nm full-width-half-maximum in the sample plane during the collection of the scan of the carious sample. The samples were studied in transmission mode with a photon energy of 1 keV, at room temperature and under vacuum. Images were collected in scanning transmission X-ray microscope (STXM) mode[60], and images were made of the sum of intensities. Ptychography was done in the same region where the STXM overview has been done before. Ptychographic reconstructions were performed using the PtyPy framework[61] using 100 iterations of the difference map algorithm followed by 100 iterations of the maximum likelihood algorithm, from the diffraction patterns. The complex of the object was obtained and the transmission and phase were reconstructed. Ptychography as a method reconstructed the complex transmission function of a specimen, i.e. $A * e^{i\alpha}$ where A is an amplitude and α is a phase. From this one can calculate the transmission as $|A|^2$ (it was also done for the data from I14). The 2D ptychography reconstruction was performed with a pixel size of 8 nm. The spatial resolution of the reconstructed images was estimated to be around 20-30 nm (this was based on judging by eye how much reasonably high-q scattering there was in the raw diffraction images, but it was not a fact and not based on any proper analysis). The analysis of the dataset was done with DAWN and Avizo. Overall correlative imaging from the different modes was carried out with Avizo. Manual positioning was done and superimposition of the dataset was performed.

-I18 XRF/XANES

One slice of a carious tooth was analysed, sample 2. The sample studied had ethical approval obtained from the National Research Ethics Committee; NHS-REC reference 14/EM/1128/ Consortium Reference BCHCDent332.1531.TB. The sample was extracted at the School of Dentistry, University of Birmingham for non-caries related therapeutic reasons. The slice was polished and then placed on a glass slide and mounted on a rotating holder in I18 (DLS, U.K.).

Light microscopy

The sample was placed on a glass side and observed using a polarized light microscope, Primotech (Zeiss, Germany) prior to the synchrotron experiment.

X-ray absorption near edge structure (XANES) in reflection X-ray fluorescence (XRF) mode

The Ca K-edge (K 1s 4038.50 eV, electron binding energy [62]) of the tooth was analysed at I18 beamline in Diamond light source (Didcot, U.K.)[63]. XANES measurements were carried out using (X-ray fluorescence) XRF in reflection mode. Ca was chosen following previous studies from Hesse et al. and Stifler et al. on the human tooth and bone[64-67] which demonstrated the polarization effect of Ca K-edge.

The sample was initially stored in phosphate-buffered saline (PBS) and then placed on a glass slide and fixed on a rotation frame. The stage of the sample was set up to have an angle of 45° with the incoming X-ray beam and the detector. Beam size was 2 µm focus using Kirkpatrick–Baez (KB) mirrors. The measurements were done at room temperature. µ-XRF in reflection mode was carried out at several energies with finer steps in the region of interest. Two silicon drift detectors, four elements each, with Xspress 3 electronics were used to collect the secondary photons. Following preliminary acquisition on enamel, maps at several energies and angles were acquired on the carious sample. The incoming beam intensity I_0 was recorded for each acquisition. µ-XANES mapping in XRF mode was done by scanning an area of the slices, with the detector 40 mm from the sample, the setup is displayed SI2-Fig. S10.

To study the polarization effect, the sample was rotated and a new map was acquired with a similar setting for steps and energy. The angle covered were 0 ° for the initial position, and 45, 90, 135 and 180 °. The maps were acquired in raster mode, with a step of 10 µm in X and Y and an exposure time of 50 ms per pixel. Due to limited time, the last angle covered had a reduced area of 0.28 × 0.89 mm^2 instead of ~1.49 × 1.31 mm^2 for the other. For the energies, a finer step was used in the region of interest of the spectrum, (i) 3950 to 4000 eV with 50 eV step, (ii) 4035 to 4040 eV with 5 eV step, (iii) 4041.5 eV, (iv) to 4045 to 4054.5 eV with 0.5 step (4055 eV was added for the last rotation), (v) 4060 to 4068 eV with 8 eV step, (vi) 4069 to 4100 eV with 31 eV step, and (vii) 4200 eV. This led to overall 30 and 31 maps (see SI2-Fig. S10 for the first rotation).
A script was written to allow automating the acquisition of the maps with only rotation of the sample done manually. For each rotation, all the energies were acquired and then the sample was rotated and the procedure is repeated. The data was then analysed using DAWN[44], OriginPro and in house scripts developed in Matlab. XANES spectra from each pixel of Ca K-edge were reconstructed from the sum of the intensity of the counts recorded from each detector and then iterating for each energy. The same procedure is used in the different rotations, a summary of the process is detailed SI2-Fig. S10. In the XANES spectra, mainly Ca was considered, as seen from the energy, which was

recorded from XRF, it is the main constituent, SI2-Fig. S10. In the reflection mode, the absorption coefficient was correlated to the intensity from the relation (Eq.2) [68]:

$$\mu(E) \propto I_f / I_0 \qquad \text{(Eq.2)}$$

With I_0 incoming intensity and I_f X-ray fluorescence yield, μ absorption coefficient and E energy. In the experiment carried out I_0 and I_f were recorded.

Full XANES spectra and the ratio of two energies were analysed in point location and region of interest. The simulation of XANES spectra was done using FDMNES program[69]. This program has been used several times for the analysis of Ca [65,67]. The position of the atoms was taken from CIF file[47], and then two polarizations were done, SI2-Fig. S11.

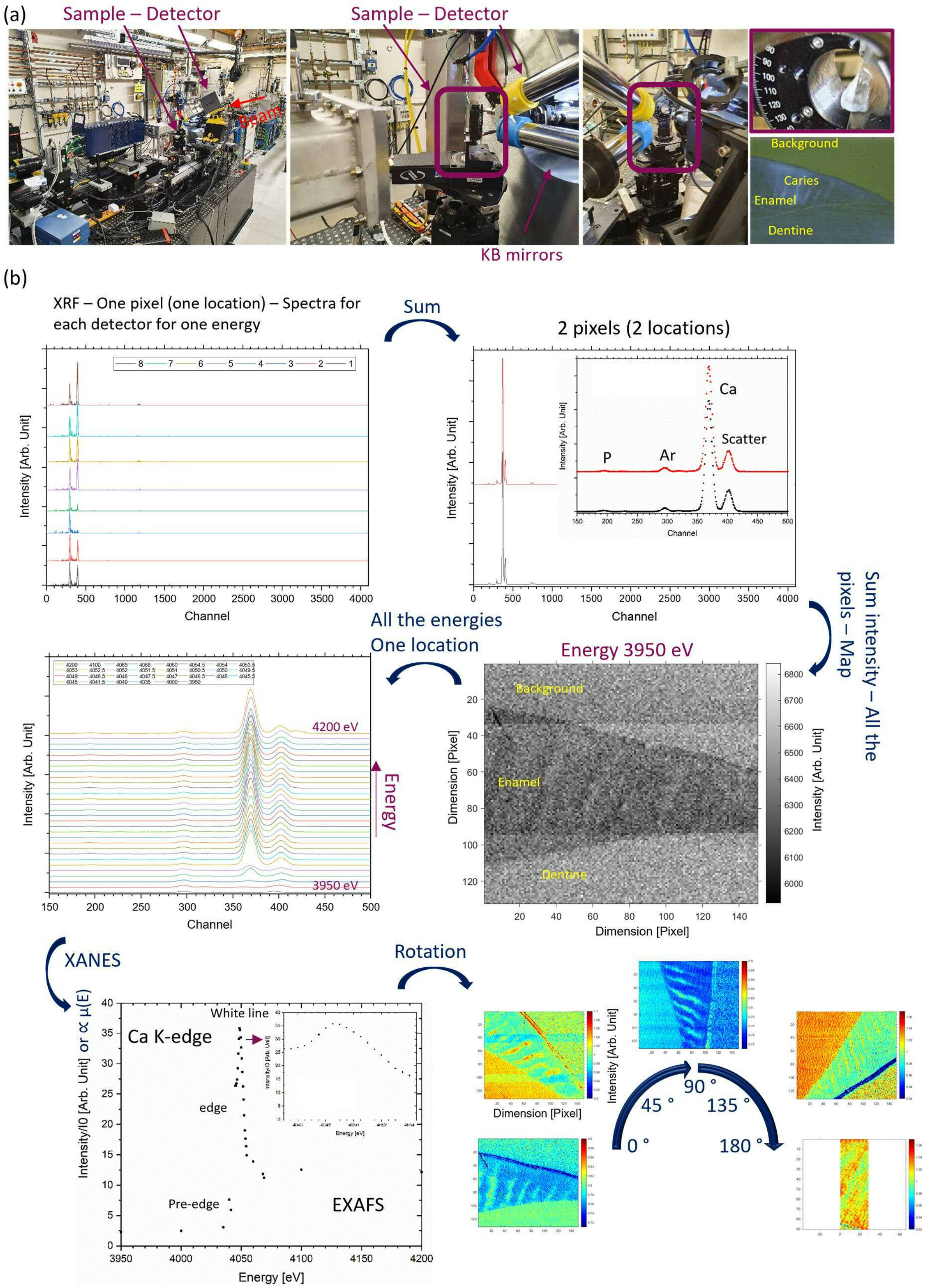

SI2-Fig. S10. Micro-XRF setup and processing of the data for the I18 experiment XRF/XANES. (a) Setup of the XANES in XRF reflection mode, (b) Step of the process of the data from spectra of individual detector to XANES spectrum and maps from the ratio of the dataset at energy 4051 eV and 4048.5 eV (both divided by I_0) at 5 rotation angles.

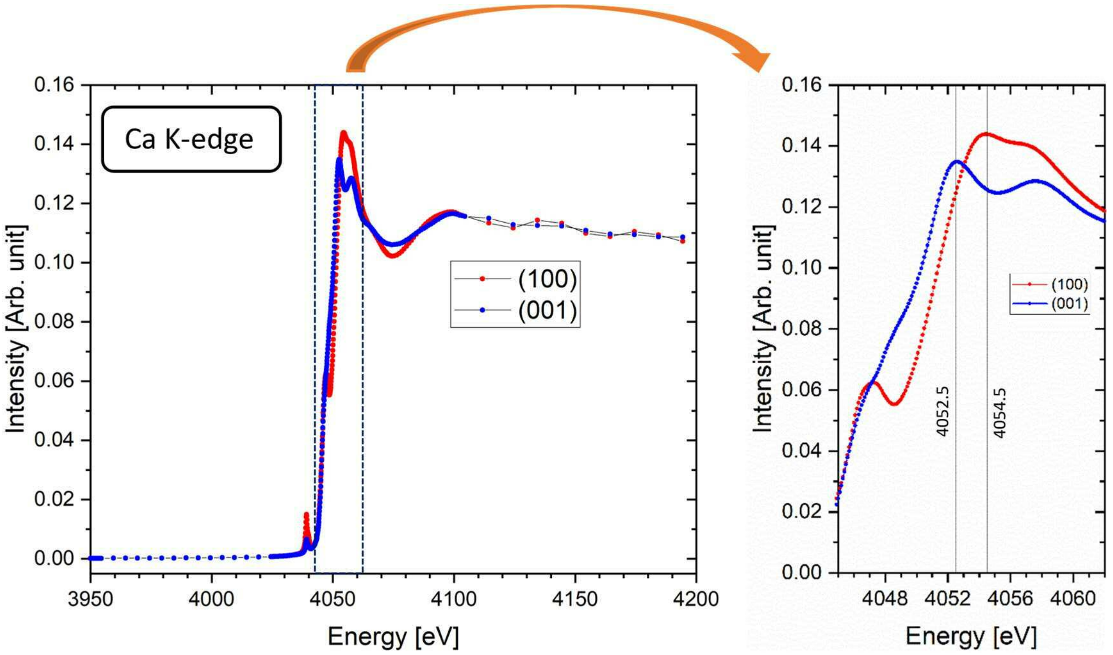

SI2-Fig. S11. XANES spectra of HAp. XANES spectra for the Ca K-edge with two polarizations using FDMNES.

-Statistical analysis

Statistical analysis in this study (e.g. XRF and tomography data) was carried out with two-sample t-test or one-way ANOVA with post hoc Tukey's test (OriginPro software) as specified in the figure captions. A p-value < 0.05 was considered statistically significant. * represents $p < 0.05$, ** represents $p \leq 0.01$, *** represents $p \leq 0.001$, and **** represents $p \leq 0.0001$.

References

1 Bolitho, E. M., Bridgewater, H. E., Needham, R. J., Coverdale, J. P. C., Quinn, P. D., Sanchez-Cano, C. & Sadler, P. J. Elemental Mapping of Half-Sandwich Azopyridine Osmium Arene Complexes in Cancer Cells. *Inorganic Chemistry Frontiers*, doi:https://doi.org/10.1039/D1QI00512J (2021).

2 Bolitho, E. M., Coverdale, J. P. C., Bridgewater, H. E., Clarkson, G. J., Quinn, P. D., Sanchez-Cano, C. & Sadler, P. J. Tracking Reactions of Asymmetric Organo-Osmium Transfer Hydrogenation Catalysts in Cancer Cells. *Angewandte Chemie International Edition* **60**, 6462-6472, doi:https://doi.org/10.1002/anie.202016456 (2021).

3 Gomez-Gonzalez, M. A., Koronfel, M. A., Pullin, H., Parker, J. E., Quinn, P. D., Inverno, M. D., Scott, T. B., Xie, F., Voulvoulis, N., Yallop, M. L., et al. Nanoscale Chemical Imaging of Nanoparticles under Real-World Wastewater Treatment Conditions. *Advanced Sustainable Systems* **n/a**, 2100023, doi:https://doi.org/10.1002/adsu.202100023 (2021).

4 Tennyson, E. M., Frohna, K., Drake, W. K., Sahli, F., Chien-Jen Yang, T., Fu, F., Werner, J., Chosy, C., Bowman, A. R., Doherty, T. A. S., et al. Multimodal Microscale Imaging of Textured Perovskite–Silicon Tandem Solar Cells. *ACS Energy Letters* **6**, 2293-2304, doi:https://doi.org/10.1021/acsenergylett.1c00568 (2021).

5 Mela, I., Poudel, C., Anaya, M., Delport, G., Frohna, K., Macpherson, S., Doherty, T. A. S., Scheeder, A., Stranks, S. D. & Kaminski, C. F. Revealing Nanomechanical Domains and Their Transient Behavior in Mixed-Halide Perovskite Films. *Advanced Functional Materials* **31**, 2100293, doi:https://doi.org/10.1002/adfm.202100293 (2021).

6 Lee, H. J., Brown, Z., Zhao, Y., Fawdon, J., Song, W., Lee, J. H., Ihli, J. & Pasta, M. Ordered $LiNi_{0.5}Mn_{1.5}O_4$ Cathode in Bis(Fluorosulfonyl)Imide-Based Ionic Liquid Electrolyte: Importance of the Cathode–Electrolyte Interphase. *Chemistry of Materials* **33**, 1238-1248, doi:https://doi.org/10.1021/acs.chemmater.0c04014 (2021).

7 Hicks, L. J., Bridges, J. C., Noguchi, T., Miyake, A., Piercy, J. D. & Baker, S. H. Fe-Redox Changes in Itokawa Space-Weathered Rims. *Meteoritics & Planetary Science* **55**, 2599-2618, doi:https://doi.org/10.1111/maps.13611 (2020).

8 Füllenbach, L. C., Perez, J. P. H., Freeman, H. M., Thomas, A. N., Mayanna, S., Parker, J. E., Göttlicher, J., Steininger, R., Radnik, J., Benning, L. G., et al. Nanoanalytical Identification of Siderite Dissolution-Coupled Pb Removal Mechanisms from Oxic and Anoxic Aqueous Solutions. *ACS Earth and Space Chemistry* **4**, 1966-1977, doi:https://doi.org/10.1021/acsearthspacechem.0c00180 (2020).

9 Brooks, J., Everett, J., Lermyte, F., Tjhin, V. T., Banerjee, S., O'Connor, P. B., Morris, C. M., Sadler, P. J., Telling, N. D. & Collingwood, J. F. Label-Free Nanoimaging of Neuromelanin in the Brain by Soft X-ray Spectromicroscopy. *Angewandte Chemie International Edition* **59**, 11984-11991, doi:https://doi.org/10.1002/anie.202000239 (2020).

10 Gomez-Gonzalez, M. A., Koronfel, M. A., Goode, A. E., Al-Ejji, M., Voulvoulis, N., Parker, J. E., Quinn, P. D., Scott, T. B., Xie, F., Yallop, M. L., et al. Spatially Resolved Dissolution and Speciation Changes of ZnO Nanorods During Short-Term *in Situ* Incubation in a Simulated Wastewater Environment. *ACS Nano* **13**, 11049-11061, doi:https://doi.org/10.1021/acsnano.9b02866 (2019).

11 McCulloch, A., Bennie, L., Coulter, J. A., McCarthy, H. O., Dromey, B., Grimes, D. R., Quinn, P., Villagomez-Bernabe, B. & Currell, F. Nuclear Uptake of Gold Nanoparticles Deduced Using Dual-Angle X-ray Fluorescence Mapping. *Particle & Particle Systems Characterization* **36**, 1900140, doi:https://doi.org/10.1002/ppsc.201900140 (2019).

12 Bower, W. R., Morris, K., Livens, F. R., Mosselmans, J. F. W., Fallon, C. M., Fuller, A. J., Natrajan, L., Boothman, C., Lloyd, J. R., Utsunomiya, S., et al. Metaschoepite Dissolution in Sediment Column Systems—Implications for Uranium Speciation and Transport.

Environmental Science & Technology **53**, 9915-9925, doi:https://doi.org/10.1021/acs.est.9b02292 (2019).

13 Morrell, A. P., Floyd, H., W. Mosselmans, J. F., Grover, L. M., Castillo-Michel, H., Davis, E. T., Parker, J. E., Martin, R. A. & Addison, O. Improving Our Understanding of Metal Implant Failures: Multiscale Chemical Imaging of Exogenous Metals in *Ex-Vivo* Biological Tissues. *Acta Biomaterialia* **98**, 284-293, doi:https://doi.org/10.1016/j.actbio.2019.05.071 (2019).

14 Carmona, A., Cloetens, P., Devès, G., Bohic, S. & Ortega, R. Nano-Imaging of Trace Metals by Synchrotron X-ray Fluorescence into Dopaminergic Single Cells and Neurite-Like Processes. *Journal of Analytical Atomic Spectrometry* **23**, 1083-1088, doi:https://doi.org/10.1039/B802242A (2008).

15 Troian, A., Otnes, G., Zeng, X., Chayanun, L., Dagytė, V., Hammarberg, S., Salomon, D., Timm, R., Mikkelsen, A., Borgström, M. T., et al. Nanobeam X-ray Fluorescence Dopant Mapping Reveals Dynamics of in Situ Zn-Doping in Nanowires. *Nano Letters* **18**, 6461-6468, doi:https://doi.org/10.1021/acs.nanolett.8b02957 (2018).

16 Victor, T. W., Easthon, L. M., Ge, M., O'Toole, K. H., Smith, R. J., Huang, X., Yan, H., Allen, K. N., Chu, Y. S. & Miller, L. M. X-ray Fluorescence Nanotomography of Single Bacteria with a Sub-15 nm Beam. *Scientific Reports* **8**, 13415, doi:https://doi.org/10.1038/s41598-018-31461-y (2018).

17 Castillo-Michel, H. A., Larue, C., Pradas del Real, A. E., Cotte, M. & Sarret, G. Practical Review on the Use of Synchrotron Based Micro- and Nano- X-ray Fluorescence Mapping and X-ray Absorption Spectroscopy to Investigate the Interactions between Plants and Engineered Nanomaterials. *Plant Physiology and Biochemistry* **110**, 13-32, doi:https://doi.org/10.1016/j.plaphy.2016.07.018 (2017).

18 Gandarias, L., Jefremovas, E. M., Gandia, D., Marcano, L., Martínez-Martínez, V., Ramos-Cabrer, P., Chevrier, D. M., Valencia, S., Fernández Barquín, L., Fdez-Gubieda, M. L., et al. Incorporation of Tb and Gd Improves the Diagnostic Functionality of Magnetotactic Bacteria. *Materials Today Bio* **20**, 100680, doi:https://doi.org/10.1016/j.mtbio.2023.100680 (2023).

19 van de Kerkhof, G. T., Walker, J. M., Agrawal, S., Clarke, S. M., Sk, M. H., Craske, D. J., Lindsay, R., Dowhyj, M., Osundare, A., Schuster, M. E., et al. An *in Situ* Liquid Environment for Synchrotron Hard X-ray Nanoprobe Microscopy. *Materials at High Temperatures*, 1-5, doi:https://doi.org/10.1080/09603409.2023.2213579 (2023).

20 Byrnes, I., Rossbach, L. M., Jaroszewicz, J., Grolimund, D., Ferreira Sanchez, D., Gomez-Gonzalez, M. A., Nuyts, G., Reinoso-Maset, E., Janssens, K., Salbu, B., et al. Synchrotron XRF and Histological Analyses Identify Damage to Digestive Tract of Uranium NP-Exposed *Daphnia Magna*. *Environmental Science & Technology* **57**, 1071-1079, doi:https://doi.org/10.1021/acs.est.2c07174 (2023).

21 Gomez-Gonzalez, M. A., Da Silva-Ferreira, T., Clark, N., Clough, R., Quinn, P. D. & Parker, J. E. Toward Understanding the Environmental Risks of Combined Microplastics/Nanomaterials Exposures: Unveiling ZnO Transformations after Adsorption onto Polystyrene Microplastics in Environmental Solutions. *Global Challenges*, 2300036, doi:https://doi.org/10.1002/gch2.202300036 (2023).

22 Chiang, Y.-H., Frohna, K., Salway, H., Abfalterer, A., Pan, L., Roose, B., Anaya, M. & Stranks, S. D. Vacuum-Deposited Wide-Bandgap Perovskite for All-Perovskite Tandem Solar Cells. *ACS Energy Letters* **8**, 2728-2737, doi:https://doi.org/10.1021/acsenergylett.3c00564 (2023).

23 Pattammattel, A., Tappero, R., Ge, M., Chu, Y. S., Huang, X., Gao, Y. & Yan, H. High-Sensitivity Nanoscale Chemical Imaging with Hard X-ray Nano-XANES. *Science Advances* **6**, eabb3615, doi:10.1126/sciadv.abb3615 (2020).

24 Streli, C., Rauwolf, M., Turyanskaya, A., Ingerle, D. & Wobrauschek, P. Elemental Imaging of Trace Elements in Bone Samples Using Micro and Nano-X-ray Fluorescence Spectrometry. *Applied Radiation and Isotopes* **149**, 200-205, doi:https://doi.org/10.1016/j.apradiso.2019.04.033 (2019).

25 Turyanskaya, A., Rauwolf, M., Pichler, V., Simon, R., Burghammer, M., Fox, O. J. L., Sawhney, K., Hofstaetter, J. G., Roschger, A., Roschger, P., et al. Detection and Imaging of Gadolinium Accumulation in Human Bone Tissue by Micro- and Submicro-XRF. *Scientific Reports* **10**, 6301, doi:https://doi.org/10.1038/s41598-020-63325-9 (2020).

26 Nazaretski, E., Yan, H., Lauer, K., Bouet, N., Huang, X., Xu, W., Zhou, J., Shu, D., Hwu, Y. & Chu, Y. S. Design and Performance of an X-Ray Scanning Microscope at the Hard X-ray Nanoprobe Beamline of NSLS-II. *Journal of Synchrotron Radiation* **24**, 1113-1119, doi:https://doi.org/10.1107/S1600577517011183 (2017).

27 Heenan, T. M. M., Wade, A., Tan, C., Parker, J. E., Matras, D., Leach, A. S., Robinson, J. B., Llewellyn, A., Dimitrijevic, A., Jervis, R., et al. Identifying the Origins of Microstructural Defects Such as Cracking within Ni-Rich NMC811 Cathode Particles for Lithium-Ion Batteries. *Advanced Energy Materials* **10**, 2002655, doi:https://doi.org/10.1002/aenm.202002655 (2020).

28 Wittig, N. K., Palle, J., Østergaard, M., Frølich, S., Birkbak, M. E., Spiers, K. M., Garrevoet, J. & Birkedal, H. Bone Biomineral Properties Vary across Human Osteonal Bone. *ACS Nano* **13**, 12949-12956, doi:https://doi.org/10.1021/acsnano.9b05535 (2019).

29 Naji, S., Stock, S. R., Rendu, W., Gourichon, L., Colard, T. & Cai, Z. Recent Advances on Acellular Cementum Increments Composition Using Synchrotron X-Radiation. in *Dental Cementum in Anthropology* (eds Lionel Gourichon, Stephan Naji, & William Rendu) 110-137 (Cambridge University Press, 2022), doi:https://doi.org/10.1017/9781108569507.008.

30 Silva Barreto, I., Le Cann, S., Ahmed, S., Sotiriou, V., Turunen, M. J., Johansson, U., Rodriguez-Fernandez, A., Grünewald, T. A., Liebi, M., Nowlan, N. C., et al. Multiscale Characterization of Embryonic Long Bone Mineralization in Mice. *Advanced Science* **7**, 2002524, doi:https://doi.org/10.1002/advs.202002524 (2020).

31 Palle, J., Wittig, N. K., Kubec, A., Niese, S., Rosenthal, M., Burghammer, M., Grünewald, T. A. & Birkedal, H. Nanobeam X-ray Fluorescence and Diffraction Computed Tomography on Human Bone with a Resolution Better Than 120 nm. *Journal of Structural Biology* **212**, 107631, doi:https://doi.org/10.1016/j.jsb.2020.107631 (2020).

32 Besnard, C., Marie, A., Buček, P., Sasidharan, S., Harper, R. A., Marathe, S., Wanelik, K., Landini, G., Shelton, R. M. & Korsunsky, A. M. Hierarchical 2D to 3D Micro/Nano-Histology of Human Dental Caries Lesions Using Light, X-ray and Electron Microscopy. *Materials & Design* **220**, 110829, doi:https://doi.org/10.1016/j.matdes.2022.110829 (2022).

33 Besnard, C., Marie, A., Sasidharan, S., Buček, P., Walker, J., Parker, J. E., Moxham, T. E. J., Daurer, B., Kaulich, B., Kazemian, M., et al. Nanoscale Correlative X-ray Spectroscopy and Ptychography of Carious Dental Enamel. *Materials & Design* **224**, 111272, doi:https://doi.org/10.1016/j.matdes.2022.111272 (2022).

34 Besnard, C., Harper, R. A., Moxham, T. E. J., James, J. D., Storm, M., Salvati, E., Landini, G., Shelton, R. M. & Korsunsky, A. M. 3D Analysis of Enamel Demineralisation in Human Dental Caries Using High-Resolution, Large Field of View Synchrotron X-ray Micro-Computed Tomography. *Materials Today Communications* **27**, 102418, doi:https://doi.org/10.1016/j.mtcomm.2021.102418 (2021).

35 Ronneberger, O., Fischer, P. & Brox, T. U-Net: Convolutional Networks for Biomedical Image Segmentation. in *Medical Image Computing and Computer-Assisted Intervention – MICCAI 2015 - 18th International Conference* (eds Navab, N., Hornegger, J., Wells, W., Frangi, A.). Lecture Notes in Computer Science **9351**, 234-241 (Springer International Publishing, Cham, Switzerland, 2015), doi:https://doi.org/10.1007/978-3-319-24574-4_28.

36 Ronneberger, O., Fischer, P. & Brox, T. U-Net: Convolutional Networks for Biomedical Image Segmentation. *arXiv:1505.04597*, 1-8, doi:https://doi.org/10.48550/arXiv.1505.04597 (2015).

37 Rau, C. Imaging with Coherent Synchrotron Radiation: X-ray Imaging and Coherence Beamline (I13) at Diamond Light Source. *Synchrotron Radiation News* **30**, 19-25, doi:https://doi.org/10.1080/08940886.2017.1364530 (2017).

38 Deyhle, H., Weitkamp, T., Lang, S., Schulz, G., Rack, A., Zanette, I. & Müller, B. Comparison of Propagation-Based Phase-Contrast Tomography Approaches for the Evaluation of Dentin Microstructure. Proc. SPIE 8506, Developments in X-Ray Tomography VIII, 85060N, doi:https://doi.org/10.1117/12.929951 (2012).

39 Zabler, S., Riesemeier, H., Fratzl, P. & Zaslansky, P. Fresnel-Propagated Imaging for the Study of Human Tooth Dentin by Partially Coherent X-ray Tomography. *Optics Express* **14**, 8584-8597, doi:https://doi.org/10.1364/OE.14.008584 (2006).

40 Quinn, P. D., Alianelli, L., Gomez-Gonzalez, M., Mahoney, D., Cacho-Nerin, F., Peach, A. & Parker, J. E. The Hard X-ray Nanoprobe Beamline at Diamond Light Source. *Journal of Synchrotron Radiation* **28**, 1006-1013, doi:https://doi.org/10.1107/S1600577521002502 (2021).

41 Kirkpatrick, P. & Baez, A. V. Formation of Optical Images by X-rays. *Journal of the Optical Society of America* **38**, 766-774, doi:10.1364/JOSA.38.000766 (1948).

42 Quinn, P., Parker, J., Cacho-Nerin, F., Walker, M. & Howes, P. The Hard X-ray Nanoprobe Beamline at Diamond - Current Status. *Microscopy and Microanalysis* **24**, 242-243, doi:https://doi.org/10.1017/S1431927618013569 (2018).

43 Walker, M., Howes, P., McNutt, P., Harvey, D., Dong, H., Cacho-Nerin, F. & Quinn, P. Characterisation of Cold Sprayed Ni Alloy 718 Coatings. in *ITSC 2018—Proceedings of the International Thermal Spray Conference* (ed ASM International) 248-255 (Orlando, Florida, USA, 2018).

44 Basham, M., Filik, J., Wharmby, M. T., Chang, P. C. Y., El Kassaby, B., Gerring, M., Aishima, J., Levik, K., Pulford, B. C. A., Sikharulidze, I., et al. Data Analysis Workbench (DAWN). *Journal of Synchrotron Radiation* **22**, 853-858, doi:https://doi.org/10.1107/S1600577515002283 (2015).

45 Solé, V. A., Papillon, E., Cotte, M., Walter, P. & Susini, J. A Multiplatform Code for the Analysis of Energy-Dispersive X-Ray Fluorescence Spectra. *Spectrochimica Acta Part B: Atomic Spectroscopy* **62**, 63-68, doi:https://doi.org/10.1016/j.sab.2006.12.002 (2007).

46 Besnard, C., Harper, R. A., Salvati, E., Moxham, T. E. J., Romano Brandt, L., Landini, G., Shelton, R. M. & Korsunsky, A. M. Analysis of *in Vitro* Demineralised Human Enamel Using Multi-Scale Correlative Optical and Scanning Electron Microscopy, and High-Resolution Synchrotron Wide-Angle X-ray Scattering. *Materials & Design* **206**, 109739, doi:https://doi.org/10.1016/j.matdes.2021.109739 (2021).

47 Hughes, J. M., Cameron, M. & Kevin, D. C. Structural Variations in Natural F, OH, and Cl Apatites. *American Mineralogist* **74**, 870-876 (1989).

48 Zagorac, D., Muller, H., Ruehl, S., Zagorac, J. & Rehme, S. Recent Developments in the Inorganic Crystal Structure Database: Theoretical Crystal Structure Data and Related Features. *Journal of Applied Crystallography* **52**, 918-925, doi:https://doi.org/10.1107/S160057671900997X (2019).

49 Huang, T. C., Toraya, H., Blanton, T. N. & Wu, Y. X-Ray Powder Diffraction Analysis of Silver Behenate, a Possible Low-Angle Diffraction Standard. *Journal of Applied Crystallography* **26**, 180-184, doi:https://doi.org/10.1107/S0021889892009762 (1993).

50 Sui, T., Sandholzer, M. A., Le Bourhis, E., Baimpas, N., Landini, G. & Korsunsky, A. M. Structure-Mechanical Function Relations at Nano-Scale in Heat-Affected Human Dental Tissue. *Journal of the Mechanical Behavior of Biomedical Materials* **32**, 113-124, doi:https://doi.org/10.1016/j.jmbbm.2013.12.014 (2014).

51 Schindelin, J., Arganda-Carreras, I., Frise, E., Kaynig, V., Longair, M., Pietzsch, T., Preibisch, S., Rueden, C., Saalfeld, S., Schmid, B., et al. Fiji: An Open-Source Platform for Biological-Image Analysis. *Nature Methods* **9**, 676, doi:https://doi.org/10.1038/nmeth.2019 (2012).

52 Rasband, W. S. ImageJ, U. S. National Institutes of Health, Bethesda, Maryland, USA, Https://Imagej.Nih.Gov/Ij/. (1997-2018).

53 Vogt, S., Feser, M., Legnini, D., Kirz, J. & Maser, J. Fast Differential Phase‐Contrast Imaging and Total Fluorescence Yield Mapping in a Hard X‐ray Fluorescence Microprobe. *AIP Conference Proceedings* **705**, 1348-1351, doi:https://doi.org/10.1063/1.1758051 (2004).

54 Harris, H. H., Vogt, S., Eastgate, H. & Lay, P. A. A Link between Copper and Dental Caries in Human Teeth Identified by X-ray Fluorescence Elemental Mapping. *JBIC Journal of Biological Inorganic Chemistry* **13**, 303-306, doi:https://doi.org/10.1007/s00775-007-0321-z (2008).

55 Harris, H. H., Vogt, S., Eastgate, H., Legnini, D. G., Hornberger, B., Cai, Z., Lai, B. & Lay, P. A. Migration of Mercury from Dental Amalgam through Human Teeth. *Journal of Synchrotron Radiation* **15**, 123-128, doi:https://doi.org/10.1107/S0909049507061468 (2008).

56 Parker, J. E., Gomez-Gonzalez, M., Van Lishout, Y., Islam, H., Duran Martin, D., Ozkaya, D., Quinn, P. D. & Schuster, M. E. A Cell Design for Correlative Hard X-ray Nanoprobe and Electron Microscopy Studies of Catalysts under *in Situ* Conditions. *Journal of Synchrotron Radiation* **29**, 431-438, doi:https://doi.org/10.1107/S1600577521013576 (2022).

57 Featherstone, J. D. B., O'Reilly, M. M., Shariati, M. & Brugler, S. Enhancement of Remineralisation *in Vitro* and *in Vivo*. in *Factors Relating to Demineralisation and Remineralisation of the Teeth: Proceedings of a Workshop October 5-10, 1985 Antalya, Turkey* (ed S. A. Leach) 23-34 (IRL, 1986).

58 Featherstone, J. D. B., Shariati, M., Brugler, S., Fu, J. & White, D. J. Effect of an Anticalculus Dentifrice on Lesion Progression under pH Cycling Conditions in Vitro. *Caries Research* **22**, 337-341, doi:https://doi.org/10.1159/000261133 (1988).

59 Eisenburger, M., Addy, M., Hughes, J. A. & Shellis, R. P. Effect of Time on the Remineralisation of Enamel by Synthetic Saliva after Citric Acid Erosion. *Caries Research* **35**, 211-215, doi:https://doi.org/10.1159/000047458 (2001).

60 Andrianov, K., Lühl, L., Nisius, T., Haidl, A., Gnewkow, R., Lötgering, L., Dierks, H., Kanngießer, B. & Wilhein, T. Scanning Transmission X-Ray Microscopy with X-ray Fluorescence Detection at the XUV Beamline P04, Petra III, DESY. *Journal of Physics: Conference Series* **849**, 012007, doi:10.1088/1742-6596/849/1/012007 (2017).

61 Enders, B. & Thibault, P. A Computational Framework for Ptychographic Reconstructions. *Proceedings of the Royal Society A: Mathematical, Physical and Engineering Sciences* **472**, 20160640, doi:https://doi.org/10.1098/rspa.2016.0640 (2016).

62 Thompson, A., Attwood, D., Gullikson, E., Howells, M., Kim, K.-J., Kirz, J., Kortright, J., Lindau, I., Liu, Y., Pianetta, P., et al. *X-ray Data Booklet*. (2009).

63 Mosselmans, J. F. W., Quinn, P. D., Dent, A. J., Cavill, S. A., Moreno, S. D., Peach, A., Leicester, P. J., Keylock, S. J., Gregory, S. R., Atkinson, K. D., et al. I18 - the Microfocus Spectroscopy Beamline at the Diamond Light Source. *Journal of Synchrotron Radiation* **16**, 818-824, doi:https://doi.org/10.1107/S0909049509032282 (2009).

64 Hesse, B., Stier, D., Cotte, M., Forien, J.-B. & Zaslansky, P. Polarization Induced Contrast X-ray Fluorescence at Submicrometer Resolution Reveals Nanometer Apatite Crystal Orientations across Entire Tooth Sections. *Biomedical Optics Express* **10**, 18-28, doi:10.1364/BOE.10.000018 (2019).

65 Hesse, B., Salome, M., Castillo-Michel, H., Cotte, M., Fayard, B., Sahle, C. J., De Nolf, W., Hradilova, J., Masic, A., Kanngießer, B., et al. Full-Field Calcium K-Edge X-ray Absorption near-Edge Structure Spectroscopy on Cortical Bone at the Micron-Scale: Polarization Effects Reveal Mineral Orientation. *Analytical Chemistry* **88**, 3826-3835, doi:https://doi.org/10.1021/acs.analchem.5b04898 (2016).

66 Hesse, B., Zaslansky, P., Salome, M., Castillo, H. & Cotte, M. Angular-Dependent Absorption Spectroscopy Reveals Apatite Crystal Orientation in Human Teeth. in *XRM2016: 13th International Conference on X-Ray Microscopy.* (Oxford, United Kingdom, 2016).

67 Stifler, C. A., Wittig, N. K., Sassi, M., Sun, C.-Y., Marcus, M. A., Birkedal, H., Beniash, E., Rosso, K. M. & Gilbert, P. U. P. A. X-ray Linear Dichroism in Apatite. *Journal of the American Chemical Society* **140**, 11698-11704, doi:https://doi.org/10.1021/jacs.8b05547 (2018).
68 Porcaro, F., Roudeau, S., Carmona, A. & Ortega, R. Advances in Element Speciation Analysis of Biomedical Samples Using Synchrotron-Based Techniques. *TrAC Trends in Analytical Chemistry* **104**, 22-41, doi:https://doi.org/10.1016/j.trac.2017.09.016 (2018).
69 Bunău, O. & Joly, Y. Self-Consistent Aspects of X-ray Absorption Calculations. *Journal of Physics: Condensed Matter* **21**, 345501, doi:10.1088/0953-8984/21/34/345501 (2009).
